# Supplementary material for: Patterns of Intron Gain and Loss in Fungi
Source: PLoS Biol. 2004 Nov 30;2(12):e422. doi: 10.1371/journal.pbio.0020422 (PMC532390; doi:10.1371/journal.pbio.0020422)
Supplement: Table S1 — Also available at http://genes.mit.edu/NielsenEtAl/. (4.3 MB ZIP). [file pbio.0020422.st001.zip › NielsenEtAl/index.html]

# Amino Acid Alignments with Inserted Introns

- Totals- Classifications of intron positions

  

For each ortholog set several different statistics are shown. "Totally
Conserved Introns" is the number of introns in totally conserved positions
(i.e. in positions with introns in all four species). "Gained Introns" is the
number of raw gains. "Lost introns" is the number of raw losses. "Other
Introns" is the number of other passing introns. "Fail/Homology Positions" is
the number of **positions** that failed because of low conservation or adjacency
to a gap. "Fail/Nearby Intron" is the number of positions that failed because
of a nearby intron.

Clicking on any alignment opens it up. Each intron position is color-coded and
can be clicked to view an explanation of either the reason it failed the filter
(light gray) or, if it passed, the evolutionary classification it was assigned:
raw gain (blue), raw loss (red), conserved (green), or other (dark gray).

|  |  |  |  |  |  |  |
| --- | --- | --- | --- | --- | --- | --- |
| Ortholog | Totally Conserved Introns | Gained Introns | Lost Introns | Other Introns | Fail/Homology Positions | Fail/Nearby Intron Positions |
| 1. AN0034.1.NCU03779.1.MG04014.1.FG07017.1 | 0 | 0 | 0 | 1 | 3 | 0 |
| 2. AN0037.1.NCU00273.1.MG08911.1.FG05518.1 | 0 | 0 | 0 | 2 | 12 | 0 |
| 3. AN0044.1.NCU09516.1.MG05032.1.FG02724.1 | 0 | 1 | 0 | 3 | 1 | 0 |
| 4. AN0050.1.NCU06417.1.MG06488.1.FG07302.1 | 0 | 0 | 0 | 1 | 0 | 0 |
| 5. AN0051.1.NCU06416.1.MG06487.1.FG07303.1 | 0 | 1 | 0 | 3 | 2 | 0 |
| 6. AN0057.1.NCU07755.1.MG02449.1.FG07436.1 | 0 | 3 | 1 | 9 | 2 | 0 |
| 7. AN0066.1.NCU07884.1.MG06405.1.FG05414.1 | 4 | 0 | 0 | 2 | 4 | 0 |
| 8. AN0067.1.NCU07887.1.MG06408.1.FG05409.1 | 0 | 0 | 2 | 2 | 3 | 0 |
| 9. AN0070.1.NCU05692.1.MG10973.1.FG02707.1 | 0 | 0 | 0 | 1 | 1 | 0 |
| 10. AN0072.1.NCU04153.1.MG08626.1.FG07326.1 | 4 | 0 | 0 | 0 | 0 | 0 |
| 11. AN0075.1.NCU03739.1.MG08164.1.FG07180.1 | 4 | 0 | 0 | 0 | 0 | 0 |
| 12. AN0076.1.NCU03662.1.MG08303.1.FG07011.1 | 4 | 0 | 0 | 0 | 14 | 0 |
| 13. AN0077.1.NCU01364.1.MG08311.1.FG07012.1 | 4 | 0 | 1 | 3 | 5 | 0 |
| 14. AN0081.1.NCU00440.1.MG05201.1.FG04104.1 | 8 | 0 | 0 | 0 | 2 | 0 |
| 15. AN0086.1.NCU05680.1.MG01140.1.FG02714.1 | 16 | 0 | 0 | 0 | 0 | 0 |
| 16. AN0089.1.NCU03711.1.MG08144.1.FG05141.1 | 12 | 0 | 2 | 9 | 0 | 0 |
| 17. AN0090.1.NCU06267.1.MG05255.1.FG02566.1 | 0 | 0 | 1 | 3 | 1 | 0 |
| 18. AN0091.1.NCU06266.1.MG05254.1.FG02567.1 | 4 | 0 | 0 | 0 | 0 | 0 |
| 19. AN0093.1.NCU06271.1.MG05259.1.FG02562.1 | 0 | 1 | 2 | 6 | 8 | 0 |
| 20. AN0111.1.NCU08036.1.MG07087.1.FG04284.1 | 4 | 0 | 0 | 1 | 1 | 0 |
| 21. AN0116.1.NCU04336.1.MG06071.1.FG06204.1 | 4 | 0 | 0 | 0 | 0 | 0 |
| 22. AN0117.1.NCU04337.1.MG06072.1.FG05072.1 | 4 | 0 | 0 | 0 | 8 | 0 |
| 23. AN0120.1.NCU08268.1.MG01540.1.FG09800.1 | 0 | 0 | 0 | 1 | 1 | 0 |
| 24. AN0122.1.NCU08303.1.MG07123.1.FG09725.1 | 0 | 0 | 0 | 3 | 3 | 0 |
| 25. AN0124.1.NCU07722.1.MG07119.1.FG09784.1 | 0 | 0 | 1 | 4 | 0 | 0 |
| Ortholog | Totally Conserved Introns | Gained Introns | Lost Introns | Other Introns | Fail/Homology Positions | Fail/Nearby Intron Positions |
| 26. AN0127.1.NCU03441.1.MG03139.1.FG04297.1 | 0 | 0 | 1 | 3 | 3 | 0 |
| 27. AN0129.1.NCU03426.1.MG03130.1.FG04296.1 | 4 | 0 | 0 | 2 | 1 | 0 |
| 28. AN0131.1.NCU03423.1.MG03943.1.FG01466.1 | 0 | 2 | 1 | 3 | 0 | 0 |
| 29. AN0133.1.NCU01612.1.MG03893.1.FG10757.1 | 0 | 0 | 1 | 4 | 1 | 0 |
| 30. AN0134.1.NCU01613.1.MG03894.1.FG10756.1 | 4 | 0 | 0 | 0 | 4 | 0 |
| 31. AN0137.1.NCU01747.1.MG06704.1.FG01473.1 | 4 | 0 | 0 | 4 | 5 | 0 |
| 32. AN0140.1.NCU01756.1.MG03879.1.FG10856.1 | 0 | 0 | 0 | 6 | 5 | 0 |
| 33. AN0144.1.NCU01797.1.MG07012.1.FG04382.1 | 8 | 0 | 0 | 1 | 4 | 0 |
| 34. AN0154.1.NCU08729.1.MG09574.1.FG01623.1 | 4 | 0 | 0 | 0 | 0 | 0 |
| 35. AN0158.1.NCU04443.1.MG06586.1.FG05690.1 | 0 | 2 | 0 | 0 | 1 | 0 |
| 36. AN0164.1.NCU06563.1.MG01690.1.FG05281.1 | 4 | 0 | 0 | 3 | 0 | 0 |
| 37. AN0174.1.NCU06558.1.MG08011.1.FG01730.1 | 4 | 0 | 0 | 0 | 3 | 0 |
| 38. AN0183.1.NCU08824.1.MG06153.1.FG09776.1 | 0 | 0 | 1 | 5 | 8 | 0 |
| 39. AN0200.1.NCU06311.1.MG08802.1.FG05007.1 | 0 | 0 | 0 | 1 | 1 | 0 |
| 40. AN0210.1.NCU08027.1.MG07077.1.FG04270.1 | 0 | 0 | 0 | 1 | 2 | 0 |
| 41. AN0225.1.NCU03591.1.MG04855.1.FG09017.1 | 0 | 3 | 0 | 0 | 2 | 0 |
| 42. AN0228.1.NCU05194.1.MG04129.1.FG02470.1 | 0 | 0 | 0 | 1 | 5 | 0 |
| 43. AN0229.1.NCU06986.1.MG01463.1.FG05653.1 | 0 | 0 | 1 | 3 | 7 | 0 |
| 44. AN0232.1.NCU01511.1.MG01086.1.FG04402.1 | 0 | 0 | 0 | 1 | 2 | 0 |
| 45. AN0233.1.NCU05853.1.MG08446.1.FG05720.1 | 0 | 0 | 3 | 9 | 6 | 0 |
| 46. AN0239.1.NCU00671.1.MG02823.1.FG00191.1 | 4 | 0 | 0 | 0 | 9 | 0 |
| 47. AN0240.1.NCU02136.1.MG02624.1.FG08723.1 | 4 | 1 | 2 | 9 | 0 | 0 |
| 48. AN0241.1.NCU02133.1.MG02625.1.FG08721.1 | 0 | 0 | 1 | 3 | 2 | 0 |
| 49. AN0243.1.NCU07320.1.MG04947.1.FG04985.1 | 4 | 1 | 0 | 1 | 3 | 0 |
| 50. AN0244.1.NCU02554.1.MG01238.1.FG00881.1 | 8 | 0 | 0 | 0 | 3 | 0 |
| Ortholog | Totally Conserved Introns | Gained Introns | Lost Introns | Other Introns | Fail/Homology Positions | Fail/Nearby Intron Positions |
| 51. AN0247.1.NCU02284.1.MG11039.1.FG00732.1 | 0 | 4 | 0 | 0 | 4 | 0 |
| 52. AN0250.1.NCU06138.1.MG05946.1.FG09052.1 | 0 | 3 | 1 | 20 | 5 | 0 |
| 53. AN0252.1.NCU09119.1.MG09916.1.FG06875.1 | 4 | 0 | 0 | 2 | 3 | 0 |
| 54. AN0254.1.NCU07468.1.MG09901.1.FG01227.1 | 4 | 0 | 0 | 4 | 0 | 0 |
| 55. AN0257.1.NCU00316.1.MG06332.1.FG05379.1 | 12 | 0 | 0 | 0 | 0 | 0 |
| 56. AN0260.1.NCU04646.1.MG06763.1.FG01839.1 | 0 | 0 | 2 | 9 | 0 | 0 |
| 57. AN0261.1.NCU01318.1.MG06910.1.FG01917.1 | 16 | 0 | 1 | 3 | 1 | 0 |
| 58. AN0275.1.NCU01250.1.MG07068.1.FG07129.1 | 0 | 0 | 0 | 1 | 5 | 0 |
| 59. AN0290.1.NCU07471.1.MG09902.1.FG01226.1 | 4 | 0 | 0 | 2 | 2 | 0 |
| 60. AN0293.1.NCU00954.1.MG01649.1.FG00587.1 | 4 | 0 | 0 | 1 | 2 | 0 |
| 61. AN0297.1.NCU02948.1.MG01569.1.FG01403.1 | 4 | 1 | 0 | 4 | 2 | 0 |
| 62. AN0305.1.NCU03051.1.MG04746.1.FG07055.1 | 4 | 0 | 0 | 1 | 0 | 0 |
| 63. AN0306.1.NCU03050.1.MG04745.1.FG07054.1 | 4 | 0 | 2 | 6 | 3 | 0 |
| 64. AN0312.1.NCU09247.1.MG01654.1.FG07086.1 | 0 | 1 | 0 | 0 | 0 | 0 |
| 65. AN0314.1.NCU07082.1.MG04681.1.FG01976.1 | 0 | 1 | 0 | 0 | 6 | 0 |
| 66. AN0316.1.NCU09468.1.MG06650.1.FG00639.1 | 12 | 0 | 0 | 3 | 2 | 0 |
| 67. AN0321.1.NCU01772.1.MG06984.1.FG00843.1 | 0 | 0 | 2 | 5 | 5 | 0 |
| 68. AN0327.1.NCU06854.1.MG03173.1.FG06260.1 | 0 | 0 | 1 | 3 | 2 | 0 |
| 69. AN0329.1.NCU06856.1.MG03175.1.FG06255.1 | 0 | 0 | 0 | 2 | 4 | 0 |
| 70. AN0346.1.NCU01916.1.MG02974.1.FG08931.1 | 4 | 0 | 0 | 2 | 0 | 0 |
| 71. AN0347.1.NCU01523.1.MG01079.1.FG04327.1 | 4 | 0 | 0 | 0 | 3 | 0 |
| 72. AN0349.1.NCU05427.1.MG03187.1.FG04314.1 | 0 | 0 | 0 | 3 | 3 | 0 |
| 73. AN0351.1.NCU00742.1.MG00067.1.FG05023.1 | 0 | 0 | 0 | 2 | 2 | 0 |
| 74. AN0352.1.NCU00743.1.MG00063.1.FG09704.1 | 4 | 0 | 0 | 0 | 7 | 0 |
| 75. AN0354.1.NCU09817.1.MG05734.1.FG00478.1 | 4 | 0 | 0 | 0 | 6 | 0 |
| Ortholog | Totally Conserved Introns | Gained Introns | Lost Introns | Other Introns | Fail/Homology Positions | Fail/Nearby Intron Positions |
| 76. AN0359.1.NCU02208.1.MG01978.1.FG05777.1 | 4 | 1 | 0 | 1 | 1 | 0 |
| 77. AN0380.1.NCU09819.1.MG05731.1.FG00480.1 | 0 | 0 | 0 | 4 | 4 | 0 |
| 78. AN0381.1.NCU02207.1.MG01979.1.FG05778.1 | 8 | 0 | 0 | 0 | 3 | 0 |
| 79. AN0385.1.NCU00792.1.MG02125.1.FG05696.1 | 4 | 0 | 0 | 2 | 0 | 0 |
| 80. AN0387.1.NCU08626.1.MG06836.1.FG00797.1 | 0 | 2 | 0 | 0 | 4 | 0 |
| 81. AN0403.1.NCU06061.1.MG08290.1.FG04335.1 | 8 | 0 | 2 | 2 | 3 | 0 |
| 82. AN0406.1.NCU00311.1.MG06334.1.FG05377.1 | 0 | 0 | 0 | 1 | 3 | 0 |
| 83. AN0407.1.NCU00310.1.MG06314.1.FG05376.1 | 0 | 1 | 0 | 2 | 7 | 0 |
| 84. AN0409.1.NCU00046.1.MG10199.1.FG07231.1 | 4 | 0 | 0 | 0 | 0 | 0 |
| 85. AN0410.1.NCU00043.1.MG10195.1.FG07233.1 | 8 | 0 | 0 | 0 | 2 | 0 |
| 86. AN0416.1.NCU00899.1.MG04519.1.FG00714.1 | 4 | 0 | 0 | 1 | 4 | 0 |
| 87. AN0423.1.NCU08384.1.MG03648.1.FG01523.1 | 4 | 0 | 0 | 0 | 1 | 0 |
| 88. AN0428.1.NCU00931.1.MG00161.1.FG00875.1 | 0 | 0 | 0 | 1 | 6 | 0 |
| 89. AN0431.1.NCU03127.1.MG01324.1.FG00740.1 | 0 | 0 | 1 | 9 | 7 | 0 |
| 90. AN0432.1.NCU03112.1.MG03708.1.FG00926.1 | 0 | 1 | 1 | 4 | 2 | 0 |
| 91. AN0433.1.NCU08389.1.MG03653.1.FG01516.1 | 8 | 0 | 0 | 0 | 1 | 0 |
| 92. AN0435.1.NCU08387.1.MG03651.1.FG01520.1 | 0 | 0 | 0 | 5 | 5 | 0 |
| 93. AN0436.1.NCU00898.1.MG04518.1.FG00715.1 | 0 | 0 | 0 | 1 | 4 | 0 |
| 94. AN0442.1.NCU02559.1.MG03612.1.FG09305.1 | 4 | 0 | 0 | 0 | 2 | 0 |
| 95. AN0444.1.NCU06756.1.MG01115.1.FG06272.1 | 4 | 0 | 0 | 0 | 4 | 0 |
| 96. AN0447.1.NCU01779.1.MG06993.1.FG00654.1 | 0 | 0 | 0 | 1 | 1 | 0 |
| 97. AN0451.1.NCU04156.1.MG08624.1.FG07315.1 | 0 | 0 | 2 | 2 | 2 | 0 |
| 98. AN0465.1.NCU08500.1.MG03251.1.FG00631.1 | 0 | 2 | 0 | 4 | 0 | 0 |
| 99. AN0472.1.NCU07076.1.MG01001.1.FG08757.1 | 0 | 1 | 2 | 5 | 10 | 0 |
| 100. AN0473.1.NCU06305.1.MG06644.1.FG04827.1 | 0 | 6 | 2 | 7 | 1 | 0 |
| Ortholog | Totally Conserved Introns | Gained Introns | Lost Introns | Other Introns | Fail/Homology Positions | Fail/Nearby Intron Positions |
| 101. AN0498.1.NCU09444.1.MG04897.1.FG07509.1 | 0 | 0 | 0 | 1 | 2 | 0 |
| 102. AN0504.1.NCU03436.1.MG03911.1.FG01464.1 | 8 | 0 | 0 | 1 | 1 | 0 |
| 103. AN0554.1.NCU03415.1.MG03900.1.FG00979.1 | 4 | 2 | 1 | 3 | 1 | 0 |
| 104. AN0558.1.NCU06781.1.MG06722.1.FG06037.1 | 0 | 1 | 1 | 4 | 6 | 0 |
| 105. AN0559.1.NCU06632.1.MG06097.1.FG09817.1 | 0 | 0 | 0 | 3 | 2 | 0 |
| 106. AN0560.1.NCU06631.1.MG06098.1.FG09816.1 | 0 | 1 | 2 | 2 | 5 | 0 |
| 107. AN0561.1.NCU05226.1.MG04146.1.FG09732.1 | 0 | 1 | 0 | 1 | 2 | 0 |
| 108. AN0568.1.NCU00289.1.MG08917.1.FG04683.1 | 0 | 0 | 0 | 2 | 2 | 0 |
| 109. AN0570.1.NCU03565.1.MG03965.1.FG10845.1 | 4 | 0 | 0 | 0 | 1 | 0 |
| 110. AN0575.1.NCU03364.1.MG02398.1.FG05179.1 | 0 | 1 | 0 | 1 | 3 | 0 |
| 111. AN0576.1.NCU06626.1.MG06100.1.FG05306.1 | 0 | 0 | 0 | 1 | 1 | 0 |
| 112. AN0577.1.NCU06601.1.MG06118.1.FG05259.1 | 4 | 0 | 0 | 2 | 13 | 0 |
| 113. AN0579.1.NCU07719.1.MG07125.1.FG09722.1 | 8 | 0 | 0 | 0 | 0 | 0 |
| 114. AN0583.1.NCU07712.1.MG04179.1.FG09766.1 | 0 | 0 | 0 | 3 | 1 | 0 |
| 115. AN0595.1.NCU09741.1.MG07117.1.FG09786.1 | 4 | 0 | 0 | 0 | 1 | 0 |
| 116. AN0608.1.NCU06062.1.MG08289.1.FG04334.1 | 4 | 0 | 0 | 0 | 0 | 0 |
| 117. AN0609.1.NCU06063.1.MG08288.1.FG04333.1 | 0 | 0 | 1 | 3 | 4 | 0 |
| 118. AN0630.1.NCU00160.1.MG05679.1.FG02535.1 | 4 | 0 | 0 | 0 | 4 | 0 |
| 119. AN0636.1.NCU09757.1.MG03933.1.FG00960.1 | 0 | 0 | 1 | 5 | 1 | 0 |
| 120. AN0640.1.NCU06465.1.MG06250.1.FG02522.1 | 4 | 0 | 0 | 0 | 1 | 0 |
| 121. AN0641.1.NCU06464.1.MG06249.1.FG02523.1 | 4 | 1 | 0 | 0 | 3 | 2 |
| 122. AN0646.1.NCU04242.1.MG00976.1.FG09930.1 | 0 | 0 | 1 | 3 | 0 | 0 |
| 123. AN0647.1.NCU04243.1.MG00977.1.FG09929.1 | 0 | 1 | 0 | 0 | 1 | 0 |
| 124. AN0651.1.NCU06493.1.MG00365.1.FG05535.1 | 12 | 0 | 0 | 0 | 0 | 0 |
| 125. AN0653.1.NCU00303.1.MG00364.1.FG05533.1 | 0 | 0 | 0 | 1 | 4 | 0 |
| Ortholog | Totally Conserved Introns | Gained Introns | Lost Introns | Other Introns | Fail/Homology Positions | Fail/Nearby Intron Positions |
| 126. AN0655.1.NCU06636.1.MG00757.1.FG10100.1 | 0 | 0 | 0 | 3 | 1 | 0 |
| 127. AN0665.1.NCU01002.1.MG07035.1.FG10180.1 | 4 | 0 | 0 | 1 | 3 | 0 |
| 128. AN0667.1.NCU07165.1.MG10677.1.FG04481.1 | 0 | 0 | 1 | 6 | 0 | 0 |
| 129. AN0673.1.NCU07171.1.MG10690.1.FG04485.1 | 4 | 0 | 0 | 2 | 1 | 0 |
| 130. AN0676.1.NCU03954.1.MG00961.1.FG09993.1 | 4 | 0 | 2 | 11 | 2 | 0 |
| 131. AN0686.1.NCU08946.1.MG06004.1.FG10306.1 | 4 | 0 | 0 | 0 | 0 | 0 |
| 132. AN0687.1.NCU06727.1.MG06197.1.FG10249.1 | 4 | 0 | 3 | 5 | 1 | 0 |
| 133. AN0688.1.NCU01328.1.MG02471.1.FG09998.1 | 8 | 0 | 0 | 4 | 5 | 0 |
| 134. AN0692.1.NCU06659.1.MG04106.1.FG09924.1 | 4 | 1 | 0 | 6 | 6 | 0 |
| 135. AN0697.1.NCU04427.1.MG04906.1.FG04946.1 | 8 | 0 | 0 | 0 | 12 | 0 |
| 136. AN0705.1.NCU03575.1.MG03220.1.FG10722.1 | 0 | 1 | 1 | 5 | 2 | 0 |
| 137. AN0720.1.NCU07266.1.MG10496.1.FG09628.1 | 4 | 0 | 0 | 0 | 1 | 0 |
| 138. AN0722.1.NCU07989.1.MG01762.1.FG05093.1 | 4 | 0 | 1 | 5 | 0 | 0 |
| 139. AN0727.1.NCU01637.1.MG01157.1.FG04292.1 | 0 | 1 | 0 | 0 | 0 | 0 |
| 140. AN0733.1.NCU01635.1.MG01159.1.FG04290.1 | 0 | 0 | 0 | 1 | 4 | 0 |
| 141. AN0734.1.NCU01634.1.MG06293.1.FG05491.1 | 0 | 0 | 0 | 4 | 2 | 0 |
| 142. AN0736.1.NCU04122.1.MG09188.1.FG01872.1 | 0 | 0 | 0 | 1 | 3 | 0 |
| 143. AN0747.1.NCU02549.1.MG03600.1.FG00863.1 | 16 | 0 | 0 | 0 | 0 | 0 |
| 144. AN0752.1.NCU05305.1.MG06083.1.FG09748.1 | 4 | 0 | 0 | 0 | 3 | 0 |
| 145. AN0753.1.NCU05304.1.MG06082.1.FG09747.1 | 4 | 0 | 0 | 0 | 2 | 0 |
| 146. AN0762.1.NCU04124.1.MG06735.1.FG01873.1 | 4 | 0 | 0 | 1 | 2 | 0 |
| 147. AN0764.1.NCU01979.1.MG02619.1.FG11347.1 | 0 | 0 | 1 | 3 | 1 | 0 |
| 148. AN0767.1.NCU01930.1.MG02938.1.FG08929.1 | 4 | 0 | 0 | 0 | 2 | 0 |
| 149. AN0770.1.NCU01928.1.MG02940.1.FG08927.1 | 4 | 0 | 0 | 0 | 0 | 0 |
| 150. AN0776.1.NCU03703.1.MG09194.1.FG01871.1 | 8 | 0 | 0 | 0 | 1 | 0 |
| Ortholog | Totally Conserved Introns | Gained Introns | Lost Introns | Other Introns | Fail/Homology Positions | Fail/Nearby Intron Positions |
| 151. AN0784.1.NCU03693.1.MG09218.1.FG01857.1 | 0 | 1 | 1 | 3 | 0 | 0 |
| 152. AN0785.1.NCU01213.1.MG07697.1.FG02051.1 | 0 | 0 | 1 | 6 | 0 | 0 |
| 153. AN0797.1.NCU03139.1.MG01309.1.FG00865.1 | 4 | 0 | 0 | 0 | 1 | 0 |
| 154. AN0802.1.NCU03137.1.MG01311.1.FG00866.1 | 0 | 0 | 0 | 3 | 3 | 0 |
| 155. AN0804.1.NCU02105.1.MG04650.1.FG00914.1 | 0 | 0 | 0 | 2 | 7 | 0 |
| 156. AN0808.1.NCU02104.1.MG04651.1.FG00915.1 | 4 | 0 | 0 | 3 | 0 | 0 |
| 157. AN0809.1.NCU02103.1.MG04652.1.FG00916.1 | 0 | 1 | 0 | 2 | 4 | 0 |
| 158. AN0813.1.NCU02617.1.MG01237.1.FG00661.1 | 4 | 2 | 0 | 1 | 0 | 0 |
| 159. AN0814.1.NCU02616.1.MG01236.1.FG00660.1 | 0 | 0 | 1 | 2 | 0 | 0 |
| 160. AN0817.1.NCU02621.1.MG01209.1.FG00662.1 | 0 | 0 | 0 | 7 | 2 | 0 |
| 161. AN0818.1.NCU02619.1.MG01205.1.FG00665.1 | 4 | 0 | 0 | 0 | 1 | 0 |
| 162. AN0819.1.NCU02599.1.MG01208.1.FG00898.1 | 4 | 0 | 1 | 3 | 1 | 0 |
| 163. AN0823.1.NCU06989.1.MG01456.1.FG05645.1 | 4 | 0 | 0 | 0 | 3 | 0 |
| 164. AN0824.1.NCU06543.1.MG08690.1.FG09661.1 | 0 | 1 | 2 | 2 | 4 | 0 |
| 165. AN0828.1.NCU04092.1.MG11078.1.FG03177.1 | 0 | 0 | 0 | 2 | 3 | 0 |
| 166. AN0831.1.NCU03678.1.MG09210.1.FG01852.1 | 8 | 0 | 0 | 0 | 1 | 0 |
| 167. AN0834.1.NCU00169.1.MG05320.1.FG06644.1 | 4 | 0 | 0 | 2 | 3 | 0 |
| 168. AN0843.1.NCU09475.1.MG06658.1.FG06019.1 | 4 | 0 | 0 | 0 | 2 | 0 |
| 169. AN0850.1.NCU00167.1.MG11100.1.FG06643.1 | 4 | 0 | 0 | 3 | 2 | 0 |
| 170. AN0862.1.NCU03401.1.MG06730.1.FG10822.1 | 0 | 0 | 2 | 6 | 2 | 0 |
| 171. AN0868.1.NCU00580.1.MG02999.1.FG08585.1 | 0 | 0 | 1 | 3 | 2 | 0 |
| 172. AN0870.1.NCU05390.1.MG02370.1.FG01591.1 | 0 | 3 | 0 | 1 | 3 | 0 |
| 173. AN0871.1.NCU07259.1.MG10490.1.FG09635.1 | 0 | 0 | 0 | 3 | 0 | 0 |
| 174. AN0874.1.NCU08983.1.MG06313.1.FG05389.1 | 0 | 0 | 0 | 1 | 9 | 0 |
| 175. AN0893.1.NCU09789.1.MG10691.1.FG05187.1 | 0 | 8 | 1 | 8 | 7 | 2 |
| Ortholog | Totally Conserved Introns | Gained Introns | Lost Introns | Other Introns | Fail/Homology Positions | Fail/Nearby Intron Positions |
| 176. AN0896.1.NCU03031.1.MG00666.1.FG00743.1 | 0 | 0 | 0 | 2 | 4 | 0 |
| 177. AN0906.1.NCU02011.1.MG03668.1.FG09454.1 | 8 | 1 | 0 | 5 | 3 | 0 |
| 178. AN0907.1.NCU06431.1.MG06479.1.FG07291.1 | 4 | 1 | 0 | 0 | 0 | 0 |
| 179. AN0910.1.NCU03695.1.MG09213.1.FG01865.1 | 0 | 0 | 0 | 3 | 2 | 0 |
| 180. AN0913.1.NCU09192.1.MG08368.1.FG05154.1 | 8 | 0 | 0 | 2 | 0 | 0 |
| 181. AN0918.1.NCU03492.1.MG03920.1.FG05164.1 | 0 | 0 | 0 | 1 | 2 | 0 |
| 182. AN0922.1.NCU00493.1.MG05676.1.FG05436.1 | 8 | 0 | 1 | 3 | 1 | 0 |
| 183. AN0924.1.NCU00490.1.MG05674.1.FG05434.1 | 8 | 0 | 0 | 1 | 4 | 0 |
| 184. AN0929.1.NCU03176.1.MG03007.1.FG09539.1 | 0 | 0 | 0 | 1 | 6 | 0 |
| 185. AN0930.1.NCU00589.1.MG10269.1.FG08690.1 | 0 | 0 | 4 | 8 | 6 | 0 |
| 186. AN0942.1.NCU00643.1.MG01231.1.FG00655.1 | 4 | 0 | 0 | 0 | 0 | 0 |
| 187. AN0943.1.NCU00644.1.MG01232.1.FG00656.1 | 4 | 0 | 0 | 0 | 1 | 0 |
| 188. AN0956.1.NCU00065.1.MG08825.1.FG07217.1 | 4 | 0 | 0 | 0 | 0 | 0 |
| 189. AN0981.1.NCU08976.1.MG08904.1.FG06896.1 | 4 | 0 | 0 | 0 | 0 | 0 |
| 190. AN0990.1.NCU03479.1.MG01804.1.FG00819.1 | 4 | 0 | 2 | 6 | 2 | 0 |
| 191. AN0993.1.NCU09678.1.MG10255.1.FG04668.1 | 0 | 0 | 3 | 11 | 4 | 0 |
| 192. AN0999.1.NCU08008.1.MG01722.1.FG01923.1 | 4 | 0 | 0 | 0 | 3 | 0 |
| 193. AN1002.1.NCU07698.1.MG07150.1.FG09579.1 | 0 | 0 | 1 | 2 | 3 | 0 |
| 194. AN1003.1.NCU07697.1.MG01712.1.FG09580.1 | 8 | 0 | 0 | 1 | 3 | 0 |
| 195. AN1006.1.NCU05298.1.MG06062.1.FG01947.1 | 4 | 0 | 0 | 5 | 1 | 0 |
| 196. AN1013.1.NCU04331.1.MG07048.1.FG10010.1 | 4 | 0 | 0 | 0 | 2 | 0 |
| 197. AN1014.1.NCU01751.1.MG06707.1.FG06012.1 | 0 | 0 | 0 | 8 | 2 | 0 |
| 198. AN1015.1.NCU07027.1.MG01819.1.FG09613.1 | 0 | 2 | 2 | 7 | 7 | 0 |
| 199. AN1016.1.NCU05206.1.MG01818.1.FG09614.1 | 20 | 0 | 0 | 0 | 0 | 0 |
| 200. AN1017.1.NCU07024.1.MG01822.1.FG09612.1 | 12 | 0 | 1 | 3 | 5 | 0 |
| Ortholog | Totally Conserved Introns | Gained Introns | Lost Introns | Other Introns | Fail/Homology Positions | Fail/Nearby Intron Positions |
| 201. AN1018.1.NCU07023.1.MG01823.1.FG09615.1 | 0 | 0 | 0 | 6 | 4 | 0 |
| 202. AN1019.1.NCU05204.1.MG07145.1.FG09616.1 | 4 | 0 | 0 | 0 | 5 | 0 |
| 203. AN1023.1.NCU06347.1.MG06180.1.FG09721.1 | 8 | 0 | 0 | 0 | 2 | 0 |
| 204. AN1037.1.NCU02209.1.MG01985.1.FG05784.1 | 0 | 0 | 1 | 2 | 2 | 0 |
| 205. AN1050.1.NCU09646.1.MG10700.1.FG04243.1 | 0 | 0 | 1 | 2 | 1 | 0 |
| 206. AN1056.1.NCU08485.1.MG06968.1.FG10870.1 | 0 | 0 | 0 | 1 | 2 | 0 |
| 207. AN1057.1.NCU09980.1.MG03077.1.FG09417.1 | 0 | 0 | 0 | 1 | 6 | 0 |
| 208. AN1059.1.NCU01611.1.MG06981.1.FG00840.1 | 8 | 0 | 0 | 0 | 0 | 0 |
| 209. AN1063.1.NCU02472.1.MG03073.1.FG09547.1 | 4 | 0 | 1 | 3 | 1 | 0 |
| 210. AN1065.1.NCU09203.1.MG09004.1.FG02797.1 | 0 | 0 | 0 | 1 | 4 | 0 |
| 211. AN1066.1.NCU02623.1.MG01210.1.FG00663.1 | 0 | 0 | 0 | 4 | 1 | 0 |
| 212. AN1069.1.NCU05720.1.MG03122.1.FG02729.1 | 0 | 0 | 2 | 6 | 0 | 0 |
| 213. AN1082.1.NCU03713.1.MG08146.1.FG05143.1 | 4 | 0 | 0 | 0 | 3 | 0 |
| 214. AN1084.1.NCU03737.1.MG08162.1.FG07182.1 | 0 | 0 | 3 | 6 | 0 | 0 |
| 215. AN1085.1.NCU09610.1.MG06979.1.FG00833.1 | 8 | 0 | 0 | 0 | 1 | 0 |
| 216. AN1094.1.NCU08980.1.MG06276.1.FG04130.1 | 8 | 0 | 0 | 1 | 0 | 0 |
| 217. AN1096.1.NCU05726.1.MG04929.1.FG02730.1 | 0 | 0 | 2 | 6 | 0 | 0 |
| 218. AN1103.1.NCU00446.1.MG05196.1.FG04103.1 | 0 | 2 | 1 | 2 | 1 | 0 |
| 219. AN1111.1.NCU00784.1.MG08730.1.FG09695.1 | 4 | 0 | 0 | 0 | 8 | 0 |
| 220. AN1115.1.NCU03084.1.MG04540.1.FG00465.1 | 8 | 0 | 1 | 4 | 3 | 0 |
| 221. AN1117.1.NCU03319.1.MG09309.1.FG00538.1 | 12 | 0 | 0 | 1 | 0 | 0 |
| 222. AN1126.1.NCU08340.1.MG04438.1.FG01014.1 | 12 | 0 | 0 | 0 | 0 | 0 |
| 223. AN1133.1.NCU02463.1.MG03089.1.FG09422.1 | 0 | 0 | 1 | 4 | 1 | 0 |
| 224. AN1136.1.NCU06022.1.MG07783.1.FG05701.1 | 4 | 0 | 1 | 3 | 5 | 0 |
| 225. AN1137.1.NCU06025.1.MG07781.1.FG05704.1 | 0 | 1 | 0 | 0 | 1 | 0 |
| Ortholog | Totally Conserved Introns | Gained Introns | Lost Introns | Other Introns | Fail/Homology Positions | Fail/Nearby Intron Positions |
| 226. AN1150.1.NCU05410.1.MG02375.1.FG01573.1 | 0 | 0 | 1 | 3 | 4 | 0 |
| 227. AN1151.1.NCU04071.1.MG01335.1.FG09485.1 | 8 | 0 | 0 | 0 | 1 | 0 |
| 228. AN1154.1.NCU08339.1.MG04439.1.FG01013.1 | 0 | 0 | 0 | 2 | 2 | 0 |
| 229. AN1162.1.NCU06035.1.MG04436.1.FG01008.1 | 8 | 0 | 0 | 1 | 1 | 0 |
| 230. AN1167.1.NCU02634.1.MG04443.1.FG01009.1 | 0 | 0 | 0 | 3 | 3 | 0 |
| 231. AN1168.1.NCU02762.1.MG05643.1.FG01364.1 | 0 | 0 | 1 | 7 | 1 | 0 |
| 232. AN1177.1.NCU04404.1.MG06860.1.FG02780.1 | 8 | 0 | 1 | 4 | 0 | 0 |
| 233. AN1182.1.NCU04054.1.MG00604.2.FG09530.1 | 12 | 0 | 2 | 8 | 0 | 2 |
| 234. AN1188.1.NCU02633.1.MG04442.1.FG01010.1 | 0 | 0 | 0 | 3 | 10 | 0 |
| 235. AN1189.1.NCU04736.1.MG02487.1.FG09515.1 | 4 | 0 | 0 | 1 | 2 | 2 |
| 236. AN1191.1.NCU09813.1.MG05737.1.FG01322.1 | 4 | 0 | 0 | 0 | 0 | 0 |
| 237. AN1194.1.NCU09896.1.MG06348.1.FG01329.1 | 4 | 1 | 0 | 0 | 0 | 0 |
| 238. AN1199.1.NCU08880.1.MG06782.1.FG10375.1 | 0 | 0 | 3 | 15 | 3 | 0 |
| 239. AN1207.1.NCU02965.1.MG05615.1.FG01336.1 | 0 | 0 | 1 | 2 | 4 | 0 |
| 240. AN1208.1.NCU02966.1.MG05616.1.FG01337.1 | 0 | 1 | 1 | 2 | 0 | 0 |
| 241. AN1209.1.NCU02967.1.MG05617.1.FG01338.1 | 0 | 0 | 2 | 7 | 6 | 0 |
| 242. AN1211.1.NCU02746.1.MG04827.1.FG01152.1 | 4 | 0 | 0 | 0 | 1 | 0 |
| 243. AN1216.1.NCU03068.1.MG00187.1.FG07096.1 | 4 | 1 | 0 | 0 | 2 | 1 |
| 244. AN1222.1.NCU02657.1.MG00383.1.FG00421.1 | 0 | 1 | 0 | 4 | 2 | 0 |
| 245. AN1229.1.NCU02706.1.MG04454.1.FG01020.1 | 4 | 0 | 0 | 0 | 3 | 0 |
| 246. AN1237.1.NCU02741.1.MG04832.1.FG01157.1 | 8 | 0 | 0 | 1 | 0 | 0 |
| 247. AN1246.1.NCU07914.1.MG05063.1.FG03992.1 | 8 | 0 | 0 | 0 | 0 | 0 |
| 248. AN1247.1.NCU07915.1.MG05062.1.FG03994.1 | 8 | 0 | 0 | 5 | 0 | 0 |
| 249. AN1254.1.NCU03206.1.MG01249.1.FG00477.1 | 4 | 0 | 0 | 0 | 0 | 0 |
| 250. AN1257.1.NCU00244.1.MG04092.1.FG10282.1 | 0 | 1 | 2 | 2 | 1 | 0 |
| Ortholog | Totally Conserved Introns | Gained Introns | Lost Introns | Other Introns | Fail/Homology Positions | Fail/Nearby Intron Positions |
| 251. AN1261.1.NCU07928.1.MG05157.1.FG05613.1 | 0 | 0 | 0 | 3 | 1 | 0 |
| 252. AN1263.1.NCU07930.1.MG05155.1.FG05615.1 | 0 | 0 | 0 | 4 | 1 | 0 |
| 253. AN1270.1.NCU07929.1.MG05156.1.FG05614.1 | 8 | 0 | 0 | 0 | 3 | 0 |
| 254. AN1284.1.NCU02044.1.MG10957.1.FG02764.1 | 8 | 0 | 0 | 0 | 5 | 0 |
| 255. AN1286.1.NCU04035.1.MG00590.1.FG05344.1 | 0 | 0 | 0 | 1 | 6 | 0 |
| 256. AN1287.1.NCU05633.1.MG07912.1.FG09294.1 | 4 | 0 | 0 | 0 | 2 | 0 |
| 257. AN1290.1.NCU07068.1.MG07661.1.FG02820.1 | 0 | 3 | 1 | 5 | 1 | 0 |
| 258. AN1296.1.NCU04410.1.MG05169.1.FG11594.1 | 0 | 1 | 0 | 0 | 0 | 0 |
| 259. AN1319.1.NCU01331.1.MG02467.1.FG10001.1 | 0 | 7 | 0 | 1 | 1 | 0 |
| 260. AN1334.1.NCU03883.1.MG01006.1.FG09952.1 | 4 | 0 | 0 | 0 | 0 | 0 |
| 261. AN1339.1.NCU03947.1.MG07255.1.FG10272.1 | 12 | 0 | 0 | 3 | 6 | 0 |
| 262. AN1341.1.NCU01822.1.MG02524.1.FG01552.1 | 4 | 0 | 0 | 1 | 1 | 0 |
| 263. AN1342.1.NCU01821.1.MG02525.1.FG10893.1 | 0 | 0 | 0 | 3 | 3 | 0 |
| 264. AN1354.1.NCU01022.1.MG10652.1.FG10076.1 | 0 | 0 | 0 | 4 | 5 | 0 |
| 265. AN1357.1.NCU03880.1.MG01003.1.FG10005.1 | 8 | 0 | 0 | 0 | 1 | 0 |
| 266. AN1358.1.NCU04600.1.MG01351.1.FG10239.1 | 12 | 0 | 0 | 0 | 3 | 0 |
| 267. AN1363.1.NCU06735.1.MG02518.1.FG01545.1 | 0 | 0 | 0 | 1 | 2 | 0 |
| 268. AN1364.1.NCU06751.1.MG08563.1.FG01540.1 | 0 | 0 | 1 | 3 | 1 | 0 |
| 269. AN1367.1.NCU03321.1.MG09313.1.FG11603.1 | 0 | 4 | 0 | 0 | 2 | 0 |
| 270. AN1370.1.NCU07460.1.MG09882.1.FG00518.1 | 0 | 0 | 1 | 3 | 3 | 0 |
| 271. AN1376.1.NCU04699.1.MG01295.1.FG08613.1 | 0 | 0 | 0 | 1 | 5 | 0 |
| 272. AN1377.1.NCU03937.1.MG00990.1.FG09283.1 | 0 | 0 | 0 | 1 | 6 | 0 |
| 273. AN1378.1.NCU05498.1.MG00432.1.FG08359.1 | 8 | 0 | 0 | 0 | 1 | 0 |
| 274. AN1379.1.NCU04288.1.MG00946.1.FG10280.1 | 0 | 0 | 0 | 3 | 1 | 0 |
| 275. AN1380.1.NCU07451.1.MG01046.1.FG08614.1 | 0 | 4 | 0 | 8 | 0 | 0 |
| Ortholog | Totally Conserved Introns | Gained Introns | Lost Introns | Other Introns | Fail/Homology Positions | Fail/Nearby Intron Positions |
| 276. AN1383.1.NCU03860.1.MG07265.1.FG09990.1 | 0 | 0 | 0 | 1 | 1 | 0 |
| 277. AN1384.1.NCU04316.1.MG00775.1.FG10145.1 | 8 | 0 | 0 | 0 | 5 | 0 |
| 278. AN1387.1.NCU03517.1.MG06728.1.FG06033.1 | 4 | 0 | 0 | 2 | 3 | 0 |
| 279. AN1394.1.NCU03515.1.MG06726.1.FG06035.1 | 8 | 0 | 0 | 3 | 0 | 0 |
| 280. AN1396.1.NCU05454.1.MG03147.1.FG03249.1 | 0 | 1 | 0 | 2 | 3 | 0 |
| 281. AN1403.1.NCU01013.1.MG10655.1.FG10080.1 | 4 | 0 | 0 | 0 | 0 | 0 |
| 282. AN1408.1.NCU02572.1.MG03616.1.FG09320.1 | 4 | 0 | 1 | 2 | 3 | 0 |
| 283. AN1413.1.NCU02082.1.MG00739.1.FG04907.1 | 8 | 0 | 2 | 3 | 8 | 0 |
| 284. AN1418.1.NCU04727.1.MG00625.1.FG08398.1 | 0 | 0 | 3 | 5 | 3 | 0 |
| 285. AN1433.1.NCU00761.1.MG10414.1.FG03601.1 | 0 | 1 | 0 | 1 | 2 | 0 |
| 286. AN1442.1.NCU02681.1.MG04412.1.FG01215.1 | 8 | 0 | 0 | 0 | 0 | 0 |
| 287. AN1446.1.NCU09230.1.MG10380.1.FG01177.1 | 0 | 0 | 0 | 2 | 1 | 0 |
| 288. AN1454.1.NCU09142.1.MG04771.1.FG00428.1 | 0 | 0 | 0 | 1 | 2 | 0 |
| 289. AN1455.1.NCU02708.1.MG04773.1.FG00430.1 | 4 | 0 | 0 | 1 | 8 | 0 |
| 290. AN1460.1.NCU03369.1.MG02400.1.FG05168.1 | 0 | 2 | 0 | 0 | 2 | 0 |
| 291. AN1484.1.NCU01965.1.MG04396.1.FG06850.1 | 0 | 2 | 0 | 1 | 10 | 0 |
| 292. AN1485.1.NCU03124.1.MG03696.1.FG00677.1 | 4 | 0 | 1 | 2 | 5 | 0 |
| 293. AN1489.1.NCU00226.1.MG05309.1.FG06669.1 | 0 | 0 | 0 | 4 | 1 | 0 |
| 294. AN1491.1.NCU00157.1.MG05682.1.FG02532.1 | 4 | 0 | 0 | 0 | 4 | 0 |
| 295. AN1494.1.NCU02052.1.MG03637.1.FG09406.1 | 0 | 0 | 0 | 1 | 2 | 0 |
| 296. AN1495.1.NCU09880.1.MG10740.1.FG00757.1 | 8 | 0 | 1 | 4 | 0 | 0 |
| 297. AN1518.1.NCU09804.1.MG06355.1.FG01318.1 | 0 | 0 | 0 | 5 | 11 | 0 |
| 298. AN1524.1.NCU00963.1.MG10834.1.FG00417.1 | 8 | 0 | 0 | 1 | 3 | 0 |
| 299. AN1526.1.NCU00966.1.MG10831.1.FG05601.1 | 12 | 0 | 1 | 3 | 0 | 0 |
| 300. AN1534.1.NCU00502.1.MG04752.1.FG00373.1 | 8 | 0 | 0 | 0 | 2 | 0 |
| Ortholog | Totally Conserved Introns | Gained Introns | Lost Introns | Other Introns | Fail/Homology Positions | Fail/Nearby Intron Positions |
| 301. AN1535.1.NCU03054.1.MG04759.1.FG00514.1 | 0 | 0 | 2 | 9 | 7 | 0 |
| 302. AN1536.1.NCU00499.1.MG04758.1.FG00515.1 | 0 | 0 | 1 | 3 | 0 | 0 |
| 303. AN1537.1.NCU03048.1.MG04760.1.FG00512.1 | 0 | 0 | 1 | 3 | 6 | 0 |
| 304. AN1543.1.NCU02580.1.MG03619.1.FG09341.1 | 8 | 0 | 1 | 4 | 0 | 0 |
| 305. AN1545.1.NCU09377.1.MG05637.1.FG01369.1 | 4 | 0 | 0 | 0 | 4 | 0 |
| 306. AN1547.1.NCU09770.1.MG06609.1.FG08266.1 | 8 | 0 | 1 | 2 | 0 | 0 |
| 307. AN1555.1.NCU09324.1.MG09962.1.FG01272.1 | 0 | 0 | 0 | 4 | 1 | 0 |
| 308. AN1563.1.NCU04857.1.MG05506.1.FG02203.1 | 0 | 0 | 0 | 2 | 8 | 0 |
| 309. AN1616.1.NCU07723.1.MG01713.1.FG06217.1 | 0 | 3 | 0 | 3 | 6 | 0 |
| 310. AN1625.1.NCU02321.1.MG00268.1.FG10498.1 | 0 | 0 | 3 | 5 | 8 | 0 |
| 311. AN1632.1.NCU00188.1.MG06393.1.FG05547.1 | 4 | 0 | 0 | 0 | 3 | 0 |
| 312. AN1634.1.NCU02803.1.MG01583.1.FG06849.1 | 0 | 0 | 0 | 1 | 2 | 0 |
| 313. AN1638.1.NCU09228.1.MG10606.1.FG08340.1 | 0 | 0 | 0 | 1 | 8 | 0 |
| 314. AN1662.1.NCU09442.1.MG04898.1.FG07515.1 | 8 | 0 | 0 | 0 | 3 | 0 |
| 315. AN1673.1.NCU02785.1.MG08596.1.FG01486.1 | 0 | 0 | 0 | 4 | 1 | 0 |
| 316. AN1676.1.NCU04260.1.MG00953.1.FG10392.1 | 0 | 0 | 0 | 1 | 1 | 0 |
| 317. AN1689.1.NCU00378.1.MG05008.1.FG02273.1 | 0 | 1 | 0 | 1 | 1 | 0 |
| 318. AN1695.1.NCU06039.1.MG08065.1.FG07525.1 | 0 | 0 | 1 | 4 | 3 | 0 |
| 319. AN1699.1.NCU02287.1.MG03418.1.FG10790.1 | 4 | 0 | 0 | 1 | 1 | 1 |
| 320. AN1700.1.NCU09450.1.MG05991.1.FG07499.1 | 0 | 0 | 1 | 4 | 2 | 0 |
| 321. AN1726.1.NCU09864.1.MG03840.1.FG09240.1 | 4 | 0 | 0 | 1 | 2 | 0 |
| 322. AN1731.1.NCU02936.1.MG04244.1.FG04886.1 | 0 | 0 | 2 | 2 | 4 | 0 |
| 323. AN1733.1.NCU03076.1.MG00189.1.FG01141.1 | 0 | 0 | 1 | 4 | 1 | 0 |
| 324. AN1745.1.NCU04019.1.MG01290.1.FG09476.1 | 0 | 8 | 0 | 1 | 4 | 2 |
| 325. AN1757.1.NCU05942.1.MG08343.1.FG05365.1 | 8 | 0 | 1 | 3 | 0 | 0 |
| Ortholog | Totally Conserved Introns | Gained Introns | Lost Introns | Other Introns | Fail/Homology Positions | Fail/Nearby Intron Positions |
| 326. AN1761.1.NCU10046.1.MG00600.1.FG09313.1 | 4 | 0 | 0 | 3 | 6 | 1 |
| 327. AN1766.1.NCU09551.1.MG07444.1.FG05127.1 | 0 | 2 | 0 | 1 | 3 | 0 |
| 328. AN1775.1.NCU05644.1.MG03759.1.FG02191.1 | 0 | 0 | 1 | 3 | 2 | 0 |
| 329. AN1776.1.NCU05645.1.MG03758.1.FG02192.1 | 0 | 0 | 1 | 3 | 3 | 0 |
| 330. AN1778.1.NCU10048.1.MG00599.1.FG09312.1 | 4 | 0 | 0 | 0 | 4 | 0 |
| 331. AN1779.1.NCU05972.1.MG00597.1.FG09310.1 | 4 | 0 | 0 | 0 | 1 | 0 |
| 332. AN1780.1.NCU05971.1.MG00596.1.FG09309.1 | 0 | 0 | 2 | 6 | 8 | 0 |
| 333. AN1797.1.NCU04963.1.MG06203.1.FG02641.1 | 4 | 3 | 1 | 2 | 3 | 0 |
| 334. AN1800.1.NCU04615.1.MG07312.1.FG10042.1 | 0 | 1 | 0 | 3 | 3 | 0 |
| 335. AN1810.1.NCU00194.1.MG06392.1.FG05546.1 | 0 | 0 | 1 | 3 | 2 | 0 |
| 336. AN1851.1.NCU07567.1.MG00135.1.FG06761.1 | 4 | 0 | 0 | 7 | 0 | 0 |
| 337. AN1855.1.NCU00473.1.MG04991.1.FG06730.1 | 0 | 0 | 0 | 2 | 0 | 0 |
| 338. AN1857.1.NCU09183.1.MG10969.1.FG04829.1 | 0 | 0 | 2 | 3 | 4 | 0 |
| 339. AN1859.1.NCU00359.1.MG05178.1.FG06737.1 | 0 | 0 | 0 | 2 | 2 | 0 |
| 340. AN1860.1.NCU00360.1.MG05179.1.FG06738.1 | 0 | 0 | 0 | 1 | 3 | 0 |
| 341. AN1861.1.NCU00361.1.MG05180.1.FG06739.1 | 0 | 0 | 0 | 2 | 2 | 0 |
| 342. AN1883.1.NCU02639.1.MG07169.1.FG06098.1 | 0 | 4 | 0 | 8 | 9 | 1 |
| 343. AN1892.1.NCU05623.1.MG04228.1.FG06415.1 | 4 | 1 | 0 | 1 | 1 | 0 |
| 344. AN1900.1.NCU04713.1.MG02501.1.FG09462.1 | 0 | 0 | 0 | 4 | 5 | 0 |
| 345. AN1913.1.NCU04020.1.MG04966.1.FG08761.1 | 4 | 4 | 0 | 1 | 2 | 0 |
| 346. AN1915.1.NCU04817.1.MG04969.1.FG08760.1 | 4 | 0 | 0 | 0 | 0 | 0 |
| 347. AN1918.1.NCU09873.1.MG00450.1.FG08601.1 | 8 | 4 | 0 | 3 | 1 | 0 |
| 348. AN1921.1.NCU04802.1.MG08896.1.FG09281.1 | 4 | 0 | 0 | 1 | 0 | 0 |
| 349. AN1949.1.NCU09349.1.MG06522.1.FG04350.1 | 0 | 1 | 0 | 2 | 2 | 0 |
| 350. AN1953.1.NCU04247.1.MG09547.1.FG10385.1 | 8 | 0 | 0 | 0 | 2 | 0 |
| Ortholog | Totally Conserved Introns | Gained Introns | Lost Introns | Other Introns | Fail/Homology Positions | Fail/Nearby Intron Positions |
| 351. AN1964.1.NCU08502.1.MG03236.1.FG00634.1 | 4 | 1 | 0 | 9 | 1 | 0 |
| 352. AN1965.1.NCU06970.1.MG07148.1.FG09299.1 | 0 | 22 | 3 | 5 | 4 | 1 |
| 353. AN1969.1.NCU06762.1.MG07163.1.FG04412.1 | 4 | 0 | 0 | 0 | 3 | 0 |
| 354. AN1971.1.NCU03482.1.MG03958.1.FG05150.1 | 4 | 0 | 1 | 6 | 2 | 0 |
| 355. AN1980.1.NCU08499.1.MG03253.1.FG00632.1 | 0 | 0 | 1 | 3 | 6 | 0 |
| 356. AN1989.1.NCU06761.1.MG07162.1.FG04413.1 | 4 | 0 | 1 | 4 | 0 | 0 |
| 357. AN1993.1.NCU08411.1.MG06530.1.FG10746.1 | 0 | 0 | 0 | 11 | 0 | 2 |
| 358. AN1996.1.NCU08370.1.MG03735.1.FG01498.1 | 4 | 0 | 0 | 1 | 1 | 0 |
| 359. AN2000.1.NCU05995.1.MG01282.2.FG08768.1 | 0 | 1 | 0 | 0 | 3 | 0 |
| 360. AN2002.1.NCU05999.1.MG01287.1.FG08771.1 | 12 | 0 | 0 | 1 | 3 | 0 |
| 361. AN2014.1.NCU01455.1.MG06904.1.FG01900.1 | 0 | 0 | 0 | 2 | 6 | 0 |
| 362. AN2048.1.NCU04119.1.MG06883.1.FG01890.1 | 0 | 0 | 1 | 3 | 4 | 0 |
| 363. AN2049.1.NCU04116.1.MG06877.1.FG01886.1 | 4 | 0 | 0 | 1 | 3 | 0 |
| 364. AN2052.1.NCU04117.1.MG06878.1.FG01885.1 | 0 | 0 | 0 | 1 | 1 | 0 |
| 365. AN2056.1.NCU06200.1.MG05188.1.FG06749.1 | 0 | 0 | 1 | 3 | 1 | 0 |
| 366. AN2057.1.NCU06469.1.MG06378.1.FG07257.1 | 4 | 0 | 0 | 0 | 0 | 0 |
| 367. AN2062.1.NCU03982.1.MG02503.1.FG09471.1 | 0 | 4 | 3 | 9 | 1 | 0 |
| 368. AN2063.1.NCU03981.1.MG02504.1.FG09466.1 | 0 | 0 | 0 | 2 | 8 | 0 |
| 369. AN2076.1.NCU04840.1.MG00454.1.FG08491.1 | 4 | 0 | 0 | 0 | 1 | 0 |
| 370. AN2077.1.NCU03961.1.MG06500.1.FG08439.1 | 0 | 1 | 0 | 1 | 3 | 0 |
| 371. AN2081.1.NCU04789.1.MG00448.1.FG08604.1 | 4 | 0 | 0 | 0 | 5 | 0 |
| 372. AN2085.1.NCU08605.1.MG04529.1.FG00367.1 | 4 | 0 | 0 | 1 | 0 | 0 |
| 373. AN2090.1.NCU09227.1.MG10605.1.FG07094.1 | 0 | 0 | 0 | 4 | 2 | 0 |
| 374. AN2093.1.NCU09220.1.MG05755.1.FG07083.1 | 0 | 0 | 0 | 1 | 3 | 0 |
| 375. AN2105.1.NCU00505.1.MG04741.1.FG00358.1 | 4 | 1 | 1 | 3 | 0 | 0 |
| Ortholog | Totally Conserved Introns | Gained Introns | Lost Introns | Other Introns | Fail/Homology Positions | Fail/Nearby Intron Positions |
| 376. AN2110.1.NCU01057.1.MG10254.1.FG02691.1 | 0 | 0 | 0 | 7 | 0 | 0 |
| 377. AN2113.1.NCU02739.1.MG04821.1.FG00445.1 | 4 | 1 | 0 | 0 | 3 | 0 |
| 378. AN2119.1.NCU04090.1.MG00819.1.FG09254.1 | 0 | 0 | 0 | 1 | 2 | 0 |
| 379. AN2120.1.NCU05650.1.MG05869.1.FG02233.1 | 0 | 1 | 0 | 0 | 3 | 0 |
| 380. AN2128.1.NCU06277.1.MG05272.1.FG02553.1 | 4 | 0 | 0 | 0 | 2 | 0 |
| 381. AN2130.1.NCU06500.1.MG00371.1.FG05398.1 | 4 | 0 | 1 | 3 | 4 | 0 |
| 382. AN2140.1.NCU01146.1.MG07209.1.FG07412.1 | 4 | 0 | 0 | 0 | 3 | 0 |
| 383. AN2142.1.NCU01249.1.MG07060.1.FG07141.1 | 8 | 0 | 0 | 0 | 0 | 0 |
| 384. AN2149.1.NCU04448.1.MG10358.1.FG05250.1 | 4 | 0 | 0 | 2 | 2 | 0 |
| 385. AN2150.1.NCU04449.1.MG10357.1.FG05249.1 | 0 | 0 | 1 | 4 | 2 | 0 |
| 386. AN2153.1.NCU08026.1.MG07084.1.FG04277.1 | 4 | 0 | 0 | 0 | 0 | 0 |
| 387. AN2154.1.NCU08045.1.MG07110.1.FG05066.1 | 0 | 1 | 1 | 4 | 2 | 0 |
| 388. AN2155.1.NCU08825.1.MG06151.1.FG09774.1 | 0 | 0 | 0 | 1 | 0 | 0 |
| 389. AN2174.1.NCU04370.1.MG01409.1.FG09689.1 | 0 | 0 | 0 | 5 | 9 | 0 |
| 390. AN2181.1.NCU09748.1.MG06107.1.FG05301.1 | 4 | 0 | 0 | 0 | 1 | 0 |
| 391. AN2208.1.NCU08882.1.MG05346.1.FG02075.1 | 0 | 0 | 0 | 6 | 1 | 0 |
| 392. AN2212.1.NCU01225.1.MG05478.1.FG02029.1 | 4 | 0 | 0 | 0 | 1 | 0 |
| 393. AN2216.1.NCU07000.1.MG01436.1.FG09604.1 | 0 | 0 | 1 | 3 | 8 | 0 |
| 394. AN2229.1.NCU07001.1.MG01469.1.FG05658.1 | 0 | 0 | 1 | 3 | 3 | 0 |
| 395. AN2237.1.NCU05980.1.MG02309.1.FG08454.1 | 0 | 2 | 0 | 0 | 2 | 0 |
| 396. AN2243.1.NCU07732.1.MG01743.1.FG09554.1 | 0 | 1 | 0 | 3 | 2 | 0 |
| 397. AN2244.1.NCU01183.1.MG08640.1.FG05139.1 | 0 | 0 | 1 | 6 | 4 | 2 |
| 398. AN2246.1.NCU01187.1.MG08643.1.FG05135.1 | 0 | 0 | 1 | 3 | 10 | 0 |
| 399. AN2248.1.NCU08998.1.MG01662.1.FG05554.1 | 4 | 0 | 0 | 2 | 0 | 0 |
| 400. AN2251.1.NCU09677.1.MG05525.1.FG06575.1 | 0 | 2 | 2 | 3 | 5 | 0 |
| Ortholog | Totally Conserved Introns | Gained Introns | Lost Introns | Other Introns | Fail/Homology Positions | Fail/Nearby Intron Positions |
| 401. AN2256.1.NCU07975.1.MG06094.1.FG09828.1 | 0 | 2 | 3 | 7 | 7 | 0 |
| 402. AN2260.1.NCU01142.1.MG07542.1.FG09005.1 | 0 | 1 | 2 | 3 | 5 | 0 |
| 403. AN2266.1.NCU06183.1.MG06325.1.FG05469.1 | 4 | 0 | 0 | 0 | 2 | 0 |
| 404. AN2272.1.NCU00414.1.MG06270.1.FG06932.1 | 4 | 0 | 1 | 5 | 3 | 0 |
| 405. AN2275.1.NCU00413.1.MG06269.1.FG06931.1 | 4 | 0 | 0 | 4 | 0 | 0 |
| 406. AN2282.1.NCU00195.1.MG04927.1.FG04217.1 | 0 | 1 | 0 | 6 | 4 | 4 |
| 407. AN2289.1.NCU00450.1.MG06438.1.FG06890.1 | 0 | 0 | 0 | 2 | 9 | 0 |
| 408. AN2294.1.NCU01229.1.MG05481.1.FG02040.1 | 0 | 0 | 0 | 1 | 0 | 0 |
| 409. AN2295.1.NCU01227.1.MG05480.1.FG02030.1 | 16 | 0 | 0 | 2 | 0 | 0 |
| 410. AN2298.1.NCU09006.1.MG01669.1.FG05561.1 | 0 | 0 | 0 | 1 | 1 | 0 |
| 411. AN2299.1.NCU07739.1.MG05336.1.FG02008.1 | 8 | 0 | 0 | 0 | 2 | 0 |
| 412. AN2302.1.NCU08991.1.MG04978.1.FG06922.1 | 8 | 0 | 0 | 2 | 0 | 0 |
| 413. AN2306.1.NCU06606.1.MG05093.1.FG05251.1 | 8 | 0 | 0 | 0 | 1 | 0 |
| 414. AN2308.1.NCU06624.1.MG06104.1.FG05303.1 | 0 | 0 | 0 | 6 | 5 | 0 |
| 415. AN2312.1.NCU06317.1.MG08808.1.FG05001.1 | 4 | 1 | 0 | 0 | 0 | 0 |
| 416. AN2314.1.NCU05429.1.MG03186.1.FG04313.1 | 8 | 0 | 0 | 0 | 7 | 1 |
| 417. AN2315.1.NCU05430.1.MG03185.1.FG04312.1 | 4 | 4 | 3 | 9 | 3 | 0 |
| 418. AN2316.1.NCU05457.1.MG03188.1.FG04315.1 | 4 | 0 | 0 | 0 | 1 | 0 |
| 419. AN2317.1.NCU01587.1.MG03164.1.FG06245.1 | 0 | 0 | 1 | 3 | 5 | 0 |
| 420. AN2321.1.NCU05884.1.MG02204.1.FG03636.1 | 0 | 1 | 2 | 2 | 6 | 0 |
| 421. AN2327.1.NCU00736.1.MG05569.1.FG05028.1 | 4 | 0 | 0 | 2 | 0 | 0 |
| 422. AN2332.1.NCU00959.1.MG00167.1.FG05610.1 | 4 | 0 | 0 | 0 | 3 | 0 |
| 423. AN2368.1.NCU05735.1.MG05435.1.FG04426.1 | 0 | 1 | 0 | 0 | 6 | 0 |
| 424. AN2414.1.NCU02534.1.MG09285.1.FG00376.1 | 4 | 0 | 0 | 1 | 1 | 0 |
| 425. AN2416.1.NCU02532.1.MG09283.1.FG00364.1 | 0 | 0 | 0 | 1 | 5 | 0 |
| Ortholog | Totally Conserved Introns | Gained Introns | Lost Introns | Other Introns | Fail/Homology Positions | Fail/Nearby Intron Positions |
| 426. AN2417.1.NCU02531.1.MG09282.1.FG00363.1 | 0 | 0 | 0 | 1 | 4 | 0 |
| 427. AN2435.1.NCU06783.1.MG06720.1.FG06039.1 | 0 | 1 | 0 | 0 | 2 | 0 |
| 428. AN2438.1.NCU09745.1.MG06110.1.FG05298.1 | 4 | 0 | 0 | 1 | 5 | 0 |
| 429. AN2439.1.NCU09744.1.MG06111.1.FG06219.1 | 0 | 0 | 0 | 3 | 2 | 0 |
| 430. AN2441.1.NCU08040.1.MG01832.1.FG04268.1 | 0 | 0 | 0 | 2 | 4 | 0 |
| 431. AN2447.1.NCU08511.1.MG03241.1.FG01481.1 | 4 | 0 | 0 | 0 | 2 | 0 |
| 432. AN2450.1.NCU03526.1.MG06733.1.FG06030.1 | 0 | 0 | 0 | 9 | 6 | 0 |
| 433. AN2464.1.NCU00008.1.MG05189.1.FG05525.1 | 0 | 0 | 1 | 3 | 4 | 0 |
| 434. AN2468.1.NCU04435.1.MG05107.1.FG02767.1 | 0 | 1 | 0 | 2 | 10 | 0 |
| 435. AN2475.1.NCU00821.1.MG09852.1.FG05839.1 | 0 | 1 | 0 | 1 | 2 | 0 |
| 436. AN2477.1.NCU08273.1.MG01769.1.FG09753.1 | 0 | 2 | 0 | 3 | 9 | 0 |
| 437. AN2487.1.NCU06394.1.MG06130.1.FG06388.1 | 4 | 0 | 0 | 0 | 4 | 0 |
| 438. AN2491.1.NCU04327.1.MG08122.1.FG02622.1 | 4 | 0 | 0 | 1 | 2 | 0 |
| 439. AN2493.1.NCU01376.1.MG04900.1.FG07375.1 | 0 | 7 | 1 | 4 | 2 | 0 |
| 440. AN2494.1.NCU04642.1.MG00770.1.FG10205.1 | 0 | 1 | 0 | 0 | 1 | 0 |
| 441. AN2495.1.NCU00569.1.MG03071.1.FG08552.1 | 0 | 0 | 1 | 2 | 0 | 0 |
| 442. AN2496.1.NCU00675.1.MG02781.1.FG08819.1 | 4 | 0 | 0 | 0 | 1 | 0 |
| 443. AN2497.1.NCU06530.1.MG04920.1.FG04980.1 | 0 | 0 | 0 | 2 | 0 | 0 |
| 444. AN2508.1.NCU04636.1.MG00767.1.FG10049.1 | 4 | 0 | 0 | 0 | 1 | 0 |
| 445. AN2511.1.NCU05781.1.MG08269.1.FG02639.1 | 0 | 0 | 1 | 3 | 5 | 0 |
| 446. AN2514.1.NCU04596.1.MG01345.1.FG10036.1 | 4 | 0 | 0 | 0 | 0 | 0 |
| 447. AN2518.1.NCU04252.1.MG09552.1.FG10345.1 | 4 | 1 | 1 | 6 | 2 | 0 |
| 448. AN2523.1.NCU03611.1.MG01802.1.FG10116.1 | 0 | 0 | 0 | 3 | 5 | 0 |
| 449. AN2525.1.NCU03607.1.MG09501.1.FG10119.1 | 0 | 1 | 0 | 0 | 2 | 0 |
| 450. AN2526.1.NCU03608.1.MG01808.1.FG10118.1 | 0 | 1 | 0 | 8 | 0 | 0 |
| Ortholog | Totally Conserved Introns | Gained Introns | Lost Introns | Other Introns | Fail/Homology Positions | Fail/Nearby Intron Positions |
| 451. AN2528.1.NCU09976.1.MG07677.1.FG04848.1 | 0 | 1 | 0 | 0 | 1 | 0 |
| 452. AN2529.1.NCU06647.1.MG07309.1.FG09979.1 | 0 | 0 | 2 | 6 | 0 | 0 |
| 453. AN2555.1.NCU06720.1.MG09538.1.FG05797.1 | 0 | 1 | 0 | 3 | 6 | 0 |
| 454. AN2562.1.NCU05886.1.MG05553.1.FG11567.1 | 0 | 0 | 1 | 3 | 7 | 0 |
| 455. AN2577.1.NCU09138.1.MG04839.1.FG04688.1 | 4 | 0 | 1 | 5 | 1 | 0 |
| 456. AN2717.1.NCU08435.1.MG06205.1.FG01582.1 | 0 | 0 | 0 | 1 | 7 | 0 |
| 457. AN2733.1.NCU02771.1.MG01622.1.FG01361.1 | 0 | 0 | 0 | 3 | 4 | 0 |
| 458. AN2734.1.NCU07408.1.MG04467.1.FG06827.1 | 4 | 0 | 1 | 3 | 0 | 0 |
| 459. AN2738.1.NCU08607.1.MG01245.1.FG00369.1 | 0 | 0 | 2 | 5 | 2 | 0 |
| 460. AN2745.1.NCU01276.1.MG07515.1.FG01906.1 | 4 | 0 | 0 | 0 | 1 | 0 |
| 461. AN2751.1.NCU06540.1.MG08693.1.FG09663.1 | 0 | 0 | 0 | 1 | 2 | 0 |
| 462. AN2759.1.NCU02428.1.MG03587.1.FG09853.1 | 4 | 0 | 0 | 0 | 1 | 0 |
| 463. AN2761.1.NCU02289.1.MG06562.1.FG10805.1 | 4 | 0 | 1 | 3 | 2 | 0 |
| 464. AN2762.1.NCU02291.1.MG06561.1.FG10804.1 | 4 | 0 | 0 | 1 | 5 | 0 |
| 465. AN2847.1.NCU06664.1.MG05995.1.FG10344.1 | 0 | 0 | 0 | 5 | 5 | 0 |
| 466. AN2858.1.NCU09533.1.MG07463.1.FG05130.1 | 4 | 0 | 0 | 2 | 2 | 0 |
| 467. AN2860.1.NCU00260.1.MG00220.1.FG06619.1 | 0 | 0 | 0 | 7 | 2 | 0 |
| 468. AN2862.1.NCU00243.1.MG05427.1.FG06627.1 | 8 | 0 | 0 | 2 | 1 | 0 |
| 469. AN2867.1.NCU10058.1.MG04495.1.FG00387.1 | 8 | 0 | 0 | 6 | 0 | 0 |
| 470. AN2868.1.NCU04761.1.MG02478.1.FG08476.1 | 8 | 0 | 0 | 0 | 2 | 0 |
| 471. AN2875.1.NCU07807.1.MG00223.1.FG02770.1 | 0 | 8 | 0 | 3 | 4 | 0 |
| 472. AN2877.1.NCU08578.1.MG05718.1.FG02771.1 | 4 | 0 | 1 | 3 | 2 | 0 |
| 473. AN2885.1.NCU09995.1.MG04489.1.FG00385.1 | 4 | 0 | 0 | 1 | 1 | 0 |
| 474. AN2890.1.NCU03903.1.MG01029.1.FG09264.1 | 0 | 0 | 1 | 3 | 0 | 0 |
| 475. AN2896.1.NCU09058.1.MG03335.1.FG06084.1 | 4 | 0 | 0 | 0 | 0 | 0 |
| Ortholog | Totally Conserved Introns | Gained Introns | Lost Introns | Other Introns | Fail/Homology Positions | Fail/Nearby Intron Positions |
| 476. AN2900.1.NCU09025.1.MG03390.1.FG06078.1 | 0 | 0 | 0 | 2 | 1 | 0 |
| 477. AN2901.1.NCU02333.1.MG10854.1.FG10967.1 | 0 | 0 | 1 | 3 | 2 | 0 |
| 478. AN2903.1.NCU02273.1.MG00922.1.FG10782.1 | 4 | 0 | 0 | 1 | 1 | 0 |
| 479. AN2904.1.NCU02260.1.MG00908.1.FG10769.1 | 8 | 0 | 0 | 3 | 1 | 0 |
| 480. AN2907.1.NCU05889.1.MG03317.1.FG06073.1 | 0 | 0 | 0 | 4 | 0 | 0 |
| 481. AN2909.1.NCU06717.1.MG09535.1.FG09891.1 | 4 | 0 | 0 | 1 | 0 | 0 |
| 482. AN2914.1.NCU08162.1.MG09712.1.FG03694.1 | 0 | 2 | 1 | 3 | 3 | 2 |
| 483. AN2917.1.NCU02840.1.MG01581.1.FG00559.1 | 8 | 0 | 0 | 4 | 0 | 0 |
| 484. AN2918.1.NCU02839.1.MG01582.1.FG00558.1 | 8 | 0 | 0 | 1 | 2 | 0 |
| 485. AN2925.1.NCU08373.1.MG00529.1.FG05596.1 | 0 | 0 | 0 | 4 | 5 | 0 |
| 486. AN2926.1.NCU00981.1.MG10835.1.FG01135.1 | 4 | 7 | 0 | 3 | 1 | 2 |
| 487. AN2927.1.NCU00978.1.MG10826.1.FG01137.1 | 0 | 0 | 0 | 3 | 0 | 0 |
| 488. AN2930.1.NCU03283.1.MG00550.1.FG07057.1 | 4 | 0 | 0 | 0 | 2 | 0 |
| 489. AN2934.1.NCU03281.1.MG00548.1.FG07059.1 | 4 | 0 | 0 | 0 | 3 | 0 |
| 490. AN2936.1.NCU07404.1.MG04464.1.FG06831.1 | 0 | 0 | 0 | 5 | 4 | 0 |
| 491. AN2938.1.NCU03280.1.MG00547.1.FG07060.1 | 0 | 0 | 0 | 4 | 6 | 0 |
| 492. AN2939.1.NCU03217.1.MG03684.1.FG09158.1 | 0 | 0 | 0 | 1 | 3 | 0 |
| 493. AN2940.1.NCU01324.1.MG06893.1.FG01909.1 | 0 | 0 | 0 | 1 | 0 | 0 |
| 494. AN2946.1.NCU02515.1.MG07745.1.FG05621.1 | 0 | 0 | 0 | 1 | 1 | 0 |
| 495. AN2963.1.NCU01323.1.MG06894.1.FG01910.1 | 8 | 0 | 0 | 1 | 3 | 0 |
| 496. AN2965.1.NCU01269.1.MG06892.1.FG01908.1 | 4 | 0 | 0 | 1 | 2 | 0 |
| 497. AN2967.1.NCU00950.1.MG01648.1.FG06863.1 | 0 | 0 | 0 | 3 | 5 | 0 |
| 498. AN2968.1.NCU00951.1.MG01598.1.FG00496.1 | 8 | 0 | 1 | 3 | 4 | 0 |
| 499. AN2970.1.NCU00088.1.MG10149.1.FG06718.1 | 4 | 0 | 0 | 0 | 4 | 0 |
| 500. AN2977.1.NCU07465.1.MG09906.1.FG01230.1 | 4 | 0 | 0 | 2 | 1 | 2 |
| Ortholog | Totally Conserved Introns | Gained Introns | Lost Introns | Other Introns | Fail/Homology Positions | Fail/Nearby Intron Positions |
| 501. AN2980.1.NCU09109.1.MG09927.1.FG01278.1 | 4 | 0 | 0 | 0 | 5 | 0 |
| 502. AN2981.1.NCU09111.1.MG09926.2.FG01279.1 | 8 | 0 | 0 | 4 | 5 | 0 |
| 503. AN2982.1.NCU09321.1.MG04371.1.FG01043.1 | 0 | 2 | 0 | 1 | 11 | 0 |
| 504. AN2985.1.NCU00711.1.MG10469.1.FG03726.1 | 0 | 1 | 0 | 1 | 5 | 0 |
| 505. AN2997.1.NCU03876.1.MG01013.1.FG10268.1 | 0 | 0 | 1 | 7 | 2 | 0 |
| 506. AN2998.1.NCU03877.1.MG01014.1.FG10267.1 | 0 | 0 | 1 | 3 | 8 | 0 |
| 507. AN2999.1.NCU03857.1.MG07268.1.FG10347.1 | 8 | 0 | 0 | 4 | 0 | 0 |
| 508. AN3005.1.NCU09711.1.MG03859.1.FG06052.1 | 0 | 0 | 0 | 1 | 2 | 0 |
| 509. AN3011.1.NCU09299.1.MG03486.1.FG07851.1 | 4 | 0 | 1 | 3 | 2 | 0 |
| 510. AN3013.1.NCU02233.1.MG00882.1.FG06325.1 | 0 | 0 | 1 | 4 | 3 | 0 |
| 511. AN3017.1.NCU08783.1.MG03387.1.FG01765.1 | 0 | 0 | 0 | 2 | 6 | 0 |
| 512. AN3019.1.NCU10067.1.MG03512.1.FG07938.1 | 4 | 0 | 0 | 0 | 1 | 0 |
| 513. AN3026.1.NCU10066.1.MG03511.1.FG07939.1 | 8 | 3 | 2 | 8 | 2 | 0 |
| 514. AN3029.1.NCU09721.1.MG03320.1.FG06317.1 | 4 | 0 | 1 | 9 | 1 | 0 |
| 515. AN3031.1.NCU03425.1.MG08923.1.FG05713.1 | 0 | 1 | 2 | 4 | 8 | 0 |
| 516. AN3033.1.NCU02232.1.MG00881.1.FG06339.1 | 4 | 0 | 1 | 2 | 4 | 0 |
| 517. AN3034.1.NCU06086.1.MG00843.1.FG07041.1 | 4 | 0 | 0 | 1 | 2 | 0 |
| 518. AN3035.1.NCU06089.1.MG00841.1.FG07043.1 | 0 | 0 | 0 | 2 | 1 | 0 |
| 519. AN3055.1.NCU09707.1.MG03855.1.FG06315.1 | 4 | 0 | 0 | 0 | 0 | 0 |
| 520. AN3057.1.NCU09710.1.MG03858.1.FG06053.1 | 4 | 0 | 0 | 0 | 5 | 0 |
| 521. AN3058.1.NCU02274.1.MG00923.1.FG06290.1 | 0 | 0 | 1 | 3 | 3 | 0 |
| 522. AN3059.1.NCU02252.1.MG00901.1.FG06055.1 | 0 | 0 | 1 | 6 | 1 | 0 |
| 523. AN3061.1.NCU06942.1.MG07916.1.FG10964.1 | 4 | 0 | 0 | 0 | 2 | 0 |
| 524. AN3063.1.NCU02401.1.MG03574.1.FG09837.1 | 0 | 0 | 0 | 1 | 1 | 0 |
| 525. AN3065.1.NCU02283.1.MG00925.1.FG00337.1 | 4 | 0 | 0 | 2 | 5 | 0 |
| Ortholog | Totally Conserved Introns | Gained Introns | Lost Introns | Other Introns | Fail/Homology Positions | Fail/Nearby Intron Positions |
| 526. AN3070.1.NCU09709.1.MG03857.1.FG06313.1 | 16 | 0 | 0 | 5 | 0 | 0 |
| 527. AN3082.1.NCU06869.1.MG06570.1.FG10795.1 | 0 | 0 | 1 | 3 | 0 | 0 |
| 528. AN3084.1.NCU06655.1.MG06006.1.FG10308.1 | 4 | 0 | 0 | 0 | 1 | 0 |
| 529. AN3085.1.NCU05818.1.MG08326.1.FG09881.1 | 4 | 0 | 0 | 0 | 0 | 0 |
| 530. AN3088.1.NCU01332.1.MG02466.1.FG10002.1 | 8 | 0 | 0 | 1 | 2 | 0 |
| 531. AN3095.1.NCU03576.1.MG03219.1.FG10723.1 | 0 | 0 | 0 | 1 | 8 | 0 |
| 532. AN3098.1.NCU03387.1.MG02418.1.FG10903.1 | 0 | 1 | 0 | 1 | 3 | 0 |
| 533. AN3102.1.NCU01823.1.MG06696.1.FG05996.1 | 0 | 0 | 0 | 1 | 11 | 0 |
| 534. AN3112.1.NCU01824.1.MG06695.1.FG05997.1 | 8 | 0 | 1 | 4 | 3 | 0 |
| 535. AN3113.1.NCU01826.1.MG06694.1.FG05998.1 | 4 | 0 | 1 | 3 | 3 | 0 |
| 536. AN3122.1.NCU04511.1.MG00106.1.FG04031.1 | 4 | 0 | 0 | 0 | 7 | 0 |
| 537. AN3123.1.NCU04512.1.MG00105.1.FG04030.1 | 0 | 0 | 1 | 3 | 7 | 0 |
| 538. AN3134.1.NCU01843.1.MG06639.1.FG05985.1 | 12 | 0 | 1 | 3 | 2 | 0 |
| 539. AN3144.1.NCU07584.1.MG09837.1.FG04221.1 | 0 | 0 | 0 | 1 | 5 | 0 |
| 540. AN3147.1.NCU07947.1.MG09396.1.FG01974.1 | 4 | 0 | 0 | 0 | 3 | 1 |
| 541. AN3150.1.NCU01157.1.MG07317.1.FG09991.1 | 4 | 0 | 0 | 1 | 8 | 0 |
| 542. AN3157.1.NCU09642.1.MG09838.1.FG04242.1 | 4 | 0 | 1 | 3 | 1 | 0 |
| 543. AN3163.1.NCU03388.1.MG02410.1.FG10909.1 | 0 | 1 | 0 | 1 | 2 | 0 |
| 544. AN3168.1.NCU03395.1.MG07007.1.FG10907.1 | 8 | 0 | 1 | 3 | 0 | 0 |
| 545. AN3169.1.NCU01786.1.MG06678.1.FG10840.1 | 0 | 2 | 0 | 1 | 2 | 0 |
| 546. AN3176.1.NCU03380.1.MG02417.1.FG10896.1 | 0 | 1 | 1 | 4 | 1 | 2 |
| 547. AN3190.1.NCU01879.1.MG10578.1.FG02794.1 | 4 | 0 | 0 | 0 | 1 | 0 |
| 548. AN3199.1.NCU00809.1.MG05889.1.FG03272.1 | 0 | 3 | 2 | 6 | 1 | 0 |
| 549. AN3220.1.NCU05585.1.MG05600.1.FG10483.1 | 0 | 2 | 0 | 1 | 3 | 2 |
| 550. AN3221.1.NCU05519.1.MG07843.1.FG11084.1 | 4 | 2 | 0 | 1 | 2 | 2 |
| Ortholog | Totally Conserved Introns | Gained Introns | Lost Introns | Other Introns | Fail/Homology Positions | Fail/Nearby Intron Positions |
| 551. AN3222.1.NCU01945.1.MG02955.1.FG11629.1 | 0 | 0 | 0 | 4 | 7 | 0 |
| 552. AN3305.1.NCU01107.1.MG07443.1.FG07570.1 | 0 | 0 | 1 | 3 | 3 | 0 |
| 553. AN3363.1.NCU00927.1.MG01175.1.FG00697.1 | 0 | 0 | 0 | 1 | 3 | 0 |
| 554. AN3370.1.NCU06057.1.MG09243.1.FG09385.1 | 0 | 1 | 1 | 3 | 1 | 0 |
| 555. AN3371.1.NCU06056.1.MG09244.1.FG09384.1 | 0 | 0 | 0 | 4 | 2 | 0 |
| 556. AN3390.1.NCU10045.1.MG00618.1.FG03406.1 | 0 | 0 | 0 | 4 | 3 | 0 |
| 557. AN3411.1.NCU02076.1.MG04647.1.FG00286.1 | 4 | 0 | 0 | 0 | 1 | 0 |
| 558. AN3413.1.NCU06047.1.MG09222.1.FG01509.1 | 8 | 1 | 0 | 1 | 0 | 0 |
| 559. AN3417.1.NCU02107.1.MG04646.1.FG00287.1 | 4 | 0 | 0 | 0 | 3 | 0 |
| 560. AN3421.1.NCU04933.1.MG09333.1.FG08465.1 | 0 | 0 | 1 | 3 | 3 | 0 |
| 561. AN3422.1.NCU04612.1.MG00800.1.FG09903.1 | 12 | 0 | 0 | 0 | 0 | 0 |
| 562. AN3432.1.NCU09705.1.MG03853.1.FG06059.1 | 0 | 0 | 1 | 3 | 0 | 0 |
| 563. AN3451.1.NCU09297.1.MG03487.1.FG07854.1 | 0 | 0 | 3 | 10 | 1 | 0 |
| 564. AN3456.1.NCU02430.1.MG03583.1.FG09855.1 | 0 | 0 | 0 | 2 | 3 | 0 |
| 565. AN3459.1.NCU07153.1.MG00256.1.FG09893.1 | 0 | 0 | 1 | 6 | 0 | 0 |
| 566. AN3460.1.NCU02402.1.MG03573.1.FG09838.1 | 0 | 0 | 0 | 1 | 21 | 0 |
| 567. AN3461.1.NCU05075.1.MG03479.1.FG07875.1 | 4 | 0 | 0 | 2 | 6 | 0 |
| 568. AN3466.1.NCU02438.1.MG03149.1.FG07970.1 | 12 | 0 | 0 | 6 | 2 | 0 |
| 569. AN3468.1.NCU02437.1.MG03577.1.FG11627.1 | 8 | 0 | 0 | 1 | 0 | 0 |
| 570. AN3469.1.NCU02435.1.MG03578.1.FG11626.1 | 8 | 0 | 2 | 2 | 0 | 0 |
| 571. AN3508.1.NCU05649.1.MG02290.1.FG02084.1 | 8 | 0 | 0 | 0 | 0 | 0 |
| 572. AN3515.1.NCU06522.1.MG07546.1.FG10661.1 | 0 | 1 | 0 | 0 | 1 | 0 |
| 573. AN3577.1.NCU02734.1.MG04805.1.FG00440.1 | 0 | 1 | 0 | 0 | 3 | 0 |
| 574. AN3578.1.NCU07418.1.MG04405.1.FG06839.1 | 4 | 0 | 0 | 0 | 3 | 0 |
| 575. AN3581.1.NCU08352.1.MG01284.1.FG00871.1 | 0 | 0 | 1 | 4 | 6 | 0 |
| Ortholog | Totally Conserved Introns | Gained Introns | Lost Introns | Other Introns | Fail/Homology Positions | Fail/Nearby Intron Positions |
| 576. AN3583.1.NCU03219.1.MG03742.1.FG09156.1 | 0 | 0 | 2 | 5 | 1 | 0 |
| 577. AN3584.1.NCU03218.1.MG03638.1.FG09157.1 | 4 | 0 | 0 | 0 | 0 | 0 |
| 578. AN3591.1.NCU09266.1.MG01606.1.FG00490.1 | 0 | 0 | 1 | 3 | 0 | 0 |
| 579. AN3592.1.NCU09265.1.MG01607.1.FG00491.1 | 0 | 0 | 2 | 3 | 3 | 0 |
| 580. AN3593.1.NCU09264.1.MG01608.1.FG00493.1 | 0 | 0 | 1 | 4 | 1 | 0 |
| 581. AN3594.1.NCU04137.1.MG06743.1.FG02011.1 | 4 | 0 | 1 | 3 | 3 | 0 |
| 582. AN3604.1.NCU04111.1.MG09209.1.FG01846.1 | 0 | 0 | 0 | 3 | 6 | 0 |
| 583. AN3607.1.NCU00902.1.MG04521.1.FG00710.1 | 0 | 0 | 0 | 1 | 5 | 0 |
| 584. AN3620.1.NCU00942.1.MG03676.1.FG00281.1 | 0 | 0 | 1 | 2 | 3 | 0 |
| 585. AN3622.1.NCU08343.1.MG03726.1.FG01503.1 | 4 | 0 | 0 | 0 | 1 | 0 |
| 586. AN3626.1.NCU03194.1.MG01256.1.FG10669.1 | 0 | 4 | 1 | 3 | 6 | 0 |
| 587. AN3627.1.NCU06265.1.MG05250.1.FG02585.1 | 0 | 1 | 2 | 8 | 3 | 0 |
| 588. AN3634.1.NCU04411.1.MG05168.1.FG00598.1 | 0 | 0 | 1 | 3 | 0 | 0 |
| 589. AN3641.1.NCU02702.1.MG04446.1.FG01018.1 | 4 | 0 | 0 | 0 | 0 | 0 |
| 590. AN3642.1.NCU02743.1.MG04830.1.FG01155.1 | 4 | 0 | 1 | 6 | 0 | 0 |
| 591. AN3648.1.NCU02758.1.MG05646.1.FG01291.1 | 8 | 0 | 0 | 0 | 2 | 0 |
| 592. AN3649.1.NCU02757.1.MG05647.1.FG01290.1 | 0 | 0 | 0 | 3 | 2 | 0 |
| 593. AN3655.1.NCU04753.1.MG02490.1.FG09511.1 | 4 | 0 | 0 | 0 | 0 | 0 |
| 594. AN3658.1.NCU09811.1.MG05739.1.FG09376.1 | 0 | 0 | 0 | 2 | 12 | 0 |
| 595. AN3660.1.NCU03924.1.MG01032.1.FG08400.1 | 0 | 0 | 1 | 2 | 6 | 0 |
| 596. AN3661.1.NCU03925.1.MG01033.1.FG08751.1 | 0 | 0 | 0 | 1 | 2 | 0 |
| 597. AN3665.1.NCU02632.1.MG04433.1.FG01006.1 | 0 | 0 | 1 | 7 | 7 | 0 |
| 598. AN3666.1.NCU02631.1.MG04434.1.FG01007.1 | 8 | 0 | 0 | 0 | 0 | 0 |
| 599. AN3669.1.NCU07071.1.MG00632.1.FG09275.1 | 12 | 0 | 0 | 0 | 0 | 0 |
| 600. AN3673.1.NCU02970.1.MG05621.1.FG01377.1 | 4 | 0 | 0 | 0 | 2 | 0 |
| Ortholog | Totally Conserved Introns | Gained Introns | Lost Introns | Other Introns | Fail/Homology Positions | Fail/Nearby Intron Positions |
| 601. AN3684.1.NCU08652.1.MG10529.1.FG00573.1 | 0 | 0 | 1 | 2 | 7 | 0 |
| 602. AN3686.1.NCU06107.1.MG11028.1.FG06318.1 | 4 | 0 | 0 | 0 | 0 | 0 |
| 603. AN3689.1.NCU06882.1.MG00859.1.FG07044.1 | 0 | 0 | 0 | 1 | 11 | 0 |
| 604. AN3696.1.NCU04783.1.MG04616.1.FG08485.1 | 8 | 0 | 0 | 0 | 2 | 0 |
| 605. AN3702.1.NCU09463.1.MG02432.1.FG06009.1 | 0 | 4 | 0 | 1 | 2 | 0 |
| 606. AN3708.1.NCU06876.1.MG00872.1.FG07949.1 | 4 | 0 | 0 | 0 | 6 | 0 |
| 607. AN3709.1.NCU06877.1.MG00871.1.FG07950.1 | 4 | 0 | 0 | 0 | 4 | 0 |
| 608. AN3714.1.NCU02378.1.MG03529.1.FG07960.1 | 8 | 2 | 0 | 0 | 1 | 0 |
| 609. AN3716.1.NCU02374.1.MG03524.1.FG07956.1 | 4 | 0 | 0 | 0 | 1 | 0 |
| 610. AN3719.1.NCU02393.1.MG09565.1.FG06385.1 | 12 | 0 | 0 | 0 | 0 | 0 |
| 611. AN3728.1.NCU06870.1.MG00864.1.FG07945.1 | 0 | 0 | 3 | 8 | 1 | 0 |
| 612. AN3730.1.NCU08909.1.MG08370.1.FG02022.1 | 0 | 0 | 1 | 4 | 2 | 0 |
| 613. AN3740.1.NCU03605.1.MG08206.1.FG10131.1 | 4 | 0 | 0 | 0 | 0 | 0 |
| 614. AN3745.1.NCU08121.1.MG09302.1.FG07107.1 | 0 | 1 | 0 | 0 | 1 | 0 |
| 615. AN3749.1.NCU08115.1.MG09306.1.FG07112.1 | 0 | 0 | 0 | 2 | 1 | 0 |
| 616. AN3751.1.NCU00161.1.MG05695.1.FG05442.1 | 0 | 1 | 0 | 0 | 0 | 0 |
| 617. AN3752.1.NCU02211.1.MG01976.1.FG05780.1 | 4 | 0 | 0 | 0 | 0 | 0 |
| 618. AN3756.1.NCU09290.1.MG02086.1.FG05631.1 | 12 | 0 | 0 | 3 | 3 | 0 |
| 619. AN3757.1.NCU03276.1.MG00142.1.FG07077.1 | 4 | 0 | 0 | 0 | 2 | 0 |
| 620. AN3758.1.NCU03296.1.MG04544.1.FG05598.1 | 0 | 0 | 0 | 1 | 0 | 0 |
| 621. AN3771.1.NCU06750.1.MG02510.1.FG06571.1 | 0 | 1 | 0 | 0 | 6 | 0 |
| 622. AN3778.1.NCU09746.1.MG06109.1.FG05299.1 | 0 | 0 | 2 | 6 | 0 | 0 |
| 623. AN3785.1.NCU08357.1.MG03737.1.FG00667.1 | 4 | 0 | 0 | 2 | 4 | 0 |
| 624. AN3787.1.NCU00651.1.MG03598.1.FG00271.1 | 4 | 0 | 0 | 1 | 0 | 0 |
| 625. AN3789.1.NCU02459.1.MG04091.1.FG00949.1 | 4 | 0 | 0 | 1 | 2 | 0 |
| Ortholog | Totally Conserved Introns | Gained Introns | Lost Introns | Other Introns | Fail/Homology Positions | Fail/Nearby Intron Positions |
| 626. AN3793.1.NCU07489.1.MG00149.1.FG00852.1 | 4 | 0 | 0 | 0 | 1 | 0 |
| 627. AN3795.1.NCU02114.1.MG03595.1.FG00941.1 | 4 | 0 | 0 | 0 | 0 | 0 |
| 628. AN3797.1.NCU04733.1.MG00630.1.FG08405.1 | 0 | 1 | 0 | 0 | 5 | 0 |
| 629. AN3811.1.NCU04229.1.MG06945.1.FG10221.1 | 0 | 2 | 0 | 0 | 2 | 0 |
| 630. AN3817.1.NCU00712.1.MG08975.1.FG09197.1 | 4 | 0 | 0 | 0 | 5 | 0 |
| 631. AN3823.1.NCU08627.1.MG06837.1.FG00798.1 | 4 | 0 | 0 | 0 | 0 | 0 |
| 632. AN3824.1.NCU05095.1.MG04425.1.FG01104.1 | 0 | 2 | 0 | 1 | 1 | 0 |
| 633. AN3826.1.NCU00924.1.MG01168.1.FG00694.1 | 0 | 0 | 0 | 1 | 3 | 0 |
| 634. AN3829.1.NCU00936.1.MG01230.1.FG06752.1 | 0 | 2 | 0 | 1 | 3 | 0 |
| 635. AN3830.1.NCU02450.1.MG07224.1.FG00296.1 | 0 | 0 | 0 | 1 | 3 | 0 |
| 636. AN3841.1.NCU00896.1.MG01184.1.FG00688.1 | 8 | 0 | 0 | 0 | 5 | 0 |
| 637. AN3846.1.NCU01271.1.MG02441.1.FG05903.1 | 0 | 0 | 0 | 3 | 4 | 0 |
| 638. AN3853.1.NCU01272.1.MG02440.1.FG05902.1 | 4 | 0 | 0 | 0 | 1 | 0 |
| 639. AN3855.1.NCU01275.1.MG02439.1.FG05919.1 | 0 | 1 | 0 | 0 | 3 | 0 |
| 640. AN3857.1.NCU03763.1.MG02459.1.FG07164.1 | 4 | 0 | 0 | 0 | 4 | 0 |
| 641. AN3865.1.NCU03759.1.MG06949.1.FG05909.1 | 0 | 0 | 0 | 1 | 8 | 0 |
| 642. AN3867.1.NCU03632.1.MG06947.1.FG05911.1 | 0 | 0 | 0 | 3 | 6 | 0 |
| 643. AN3869.1.NCU03633.1.MG06946.1.FG05912.1 | 0 | 0 | 0 | 3 | 5 | 0 |
| 644. AN3874.1.NCU02550.1.MG03601.1.FG00862.1 | 0 | 0 | 0 | 3 | 10 | 0 |
| 645. AN3889.1.NCU01904.1.MG02914.1.FG08943.1 | 0 | 2 | 0 | 0 | 3 | 0 |
| 646. AN3894.1.NCU04280.1.MG08584.1.FG10198.1 | 0 | 0 | 1 | 2 | 1 | 0 |
| 647. AN3895.1.NCU00052.1.MG03774.1.FG03666.1 | 0 | 0 | 1 | 3 | 4 | 0 |
| 648. AN3901.1.NCU08272.1.MG01723.1.FG05328.1 | 4 | 2 | 0 | 2 | 1 | 0 |
| 649. AN3916.1.NCU06005.1.MG01281.1.FG03247.1 | 0 | 0 | 1 | 7 | 1 | 0 |
| 650. AN3917.1.NCU09115.1.MG09920.1.FG01284.1 | 0 | 0 | 0 | 3 | 3 | 0 |
| Ortholog | Totally Conserved Introns | Gained Introns | Lost Introns | Other Introns | Fail/Homology Positions | Fail/Nearby Intron Positions |
| 651. AN3928.1.NCU06110.1.MG03098.1.FG02469.1 | 0 | 1 | 0 | 4 | 0 | 0 |
| 652. AN3932.1.NCU09309.1.MG00170.1.FG06886.1 | 4 | 0 | 0 | 4 | 0 | 0 |
| 653. AN3933.1.NCU08616.1.MG01572.1.FG05627.1 | 4 | 1 | 0 | 0 | 4 | 0 |
| 654. AN3934.1.NCU08618.1.MG01574.1.FG05625.1 | 8 | 0 | 0 | 1 | 1 | 0 |
| 655. AN3939.1.NCU02498.1.MG07731.1.FG00359.1 | 0 | 0 | 1 | 3 | 5 | 0 |
| 656. AN3940.1.NCU09261.1.MG01611.1.FG00482.1 | 0 | 0 | 1 | 6 | 3 | 0 |
| 657. AN3947.1.NCU10044.1.MG10609.1.FG01348.1 | 4 | 1 | 0 | 1 | 2 | 0 |
| 658. AN3954.1.NCU03100.1.MG00175.1.FG01111.1 | 4 | 0 | 1 | 7 | 3 | 0 |
| 659. AN3972.1.NCU04569.1.MG00771.1.FG10203.1 | 0 | 3 | 0 | 1 | 1 | 0 |
| 660. AN3973.1.NCU06031.1.MG08256.1.FG07536.1 | 0 | 0 | 0 | 1 | 3 | 0 |
| 661. AN4016.1.NCU05275.1.MG06044.1.FG01956.1 | 4 | 2 | 1 | 3 | 0 | 0 |
| 662. AN4017.1.NCU05276.1.MG06045.1.FG01957.1 | 4 | 0 | 0 | 3 | 2 | 0 |
| 663. AN4018.1.NCU03108.1.MG03702.1.FG00925.1 | 0 | 1 | 2 | 4 | 9 | 0 |
| 664. AN4019.1.NCU03107.1.MG03701.1.FG00924.1 | 0 | 1 | 2 | 5 | 6 | 0 |
| 665. AN4033.1.NCU09249.1.MG01652.1.FG07088.1 | 8 | 0 | 0 | 0 | 3 | 0 |
| 666. AN4042.1.NCU05278.1.MG06047.1.FG01959.1 | 0 | 0 | 1 | 2 | 4 | 0 |
| 667. AN4050.1.NCU02056.1.MG00652.1.FG00718.1 | 0 | 0 | 1 | 5 | 0 | 0 |
| 668. AN4051.1.NCU07352.1.MG03446.1.FG07925.1 | 0 | 0 | 2 | 6 | 0 | 0 |
| 669. AN4057.1.NCU08995.1.MG05298.1.FG04151.1 | 0 | 1 | 0 | 0 | 3 | 0 |
| 670. AN4060.1.NCU07826.1.MG05026.1.FG02588.1 | 8 | 0 | 0 | 2 | 0 | 0 |
| 671. AN4064.1.NCU09477.1.MG06656.1.FG06021.1 | 0 | 0 | 4 | 6 | 1 | 0 |
| 672. AN4073.1.NCU06432.1.MG06480.1.FG07292.1 | 0 | 1 | 0 | 0 | 3 | 0 |
| 673. AN4080.1.NCU00389.1.MG05730.1.FG07241.1 | 0 | 2 | 0 | 8 | 4 | 0 |
| 674. AN4087.1.NCU00489.1.MG05673.1.FG05433.1 | 0 | 0 | 0 | 1 | 2 | 0 |
| 675. AN4096.1.NCU06407.1.MG06243.1.FG05503.1 | 0 | 0 | 0 | 3 | 1 | 0 |
| Ortholog | Totally Conserved Introns | Gained Introns | Lost Introns | Other Introns | Fail/Homology Positions | Fail/Nearby Intron Positions |
| 676. AN4099.1.NCU01089.1.MG08846.1.FG09059.1 | 0 | 2 | 2 | 2 | 8 | 1 |
| 677. AN4101.1.NCU01852.1.MG03399.1.FG06579.1 | 0 | 0 | 0 | 2 | 3 | 0 |
| 678. AN4102.1.NCU08755.1.MG09272.1.FG06605.1 | 0 | 4 | 1 | 3 | 5 | 0 |
| 679. AN4109.1.NCU07820.1.MG02319.1.FG11475.1 | 0 | 1 | 0 | 8 | 5 | 0 |
| 680. AN4148.1.NCU01132.1.MG04780.1.FG01484.1 | 0 | 2 | 0 | 2 | 3 | 0 |
| 681. AN4159.1.NCU06724.1.MG06888.1.FG10264.1 | 0 | 5 | 0 | 0 | 3 | 0 |
| 682. AN4163.1.NCU05810.1.MG04719.1.FG09870.1 | 12 | 0 | 0 | 3 | 0 | 0 |
| 683. AN4168.1.NCU03888.1.MG02506.1.FG09497.1 | 4 | 0 | 0 | 0 | 3 | 0 |
| 684. AN4170.1.NCU03887.1.MG01045.1.FG08448.1 | 0 | 0 | 1 | 4 | 2 | 0 |
| 685. AN4171.1.NCU03886.1.MG01047.1.FG08449.1 | 0 | 0 | 1 | 3 | 2 | 0 |
| 686. AN4178.1.NCU03859.1.MG07266.1.FG09989.1 | 0 | 0 | 0 | 1 | 0 | 0 |
| 687. AN4182.1.NCU09778.1.MG01362.1.FG08468.1 | 8 | 0 | 0 | 1 | 0 | 0 |
| 688. AN4186.1.NCU09035.1.MG08318.1.FG09882.1 | 0 | 0 | 0 | 1 | 3 | 0 |
| 689. AN4189.1.NCU06419.1.MG06482.1.FG07295.1 | 4 | 0 | 0 | 1 | 4 | 0 |
| 690. AN4192.1.NCU00170.1.MG05319.1.FG06678.1 | 8 | 0 | 0 | 0 | 1 | 0 |
| 691. AN4201.1.NCU00129.1.MG10188.1.FG07277.1 | 0 | 3 | 2 | 7 | 2 | 0 |
| 692. AN4202.1.NCU01221.1.MG05449.1.FG02099.1 | 12 | 0 | 1 | 2 | 0 | 0 |
| 693. AN4208.1.NCU04144.1.MG06765.1.FG02016.1 | 0 | 0 | 0 | 2 | 6 | 0 |
| 694. AN4209.1.NCU00025.1.MG06336.1.FG05384.1 | 4 | 0 | 0 | 1 | 3 | 0 |
| 695. AN4215.1.NCU01219.1.MG05447.1.FG02097.1 | 0 | 0 | 1 | 3 | 1 | 0 |
| 696. AN4217.1.NCU03761.1.MG06950.1.FG07162.1 | 0 | 0 | 1 | 3 | 4 | 0 |
| 697. AN4218.1.NCU02003.1.MG03641.1.FG08811.1 | 4 | 3 | 0 | 3 | 2 | 0 |
| 698. AN4222.1.NCU01827.1.MG06693.1.FG05999.1 | 0 | 0 | 0 | 1 | 4 | 0 |
| 699. AN4226.1.NCU01595.1.MG03210.1.FG01446.1 | 0 | 6 | 0 | 1 | 2 | 0 |
| 700. AN4231.1.NCU07456.1.MG04586.1.FG01131.1 | 0 | 0 | 0 | 1 | 1 | 0 |
| Ortholog | Totally Conserved Introns | Gained Introns | Lost Introns | Other Introns | Fail/Homology Positions | Fail/Nearby Intron Positions |
| 701. AN4232.1.NCU04503.1.MG00087.1.FG04027.1 | 0 | 0 | 0 | 4 | 0 | 0 |
| 702. AN4235.1.NCU08609.1.MG04527.1.FG01138.1 | 8 | 0 | 0 | 0 | 2 | 0 |
| 703. AN4239.1.NCU07290.1.MG05378.1.FG01193.1 | 4 | 0 | 0 | 0 | 1 | 0 |
| 704. AN4245.1.NCU04721.1.MG07051.1.FG04738.1 | 0 | 0 | 0 | 2 | 4 | 0 |
| 705. AN4255.1.NCU04728.1.MG00623.1.FG08399.1 | 0 | 0 | 0 | 3 | 2 | 0 |
| 706. AN4259.1.NCU02813.1.MG01595.1.FG07122.1 | 0 | 0 | 0 | 1 | 1 | 0 |
| 707. AN4264.1.NCU02811.1.MG01593.1.FG06842.1 | 0 | 0 | 1 | 3 | 3 | 0 |
| 708. AN4268.1.NCU03949.1.MG07261.1.FG10065.1 | 0 | 0 | 0 | 3 | 5 | 0 |
| 709. AN4279.1.NCU02814.1.MG01596.1.FG07121.1 | 0 | 0 | 1 | 8 | 4 | 0 |
| 710. AN4280.1.NCU03556.1.MG03247.1.FG00308.1 | 8 | 0 | 0 | 0 | 1 | 0 |
| 711. AN4281.1.NCU08477.1.MG06962.1.FG10873.1 | 12 | 0 | 0 | 0 | 1 | 0 |
| 712. AN4282.1.NCU02319.1.MG09190.1.FG01604.1 | 0 | 1 | 0 | 9 | 6 | 2 |
| 713. AN4284.1.NCU01766.1.MG03153.1.FG05204.1 | 0 | 0 | 0 | 3 | 2 | 0 |
| 714. AN4295.1.NCU04307.1.MG00965.1.FG10319.1 | 0 | 0 | 0 | 1 | 0 | 0 |
| 715. AN4299.1.NCU08949.1.MG06000.1.FG10303.1 | 0 | 0 | 1 | 3 | 3 | 0 |
| 716. AN4304.1.NCU04098.1.MG09542.1.FG04567.1 | 0 | 0 | 2 | 2 | 3 | 0 |
| 717. AN4310.1.NCU01847.1.MG02514.1.FG01544.1 | 4 | 0 | 0 | 1 | 17 | 0 |
| 718. AN4313.1.NCU06847.1.MG03205.1.FG04370.1 | 0 | 1 | 0 | 1 | 3 | 0 |
| 719. AN4317.1.NCU04063.1.MG00641.1.FG09271.1 | 4 | 0 | 0 | 1 | 7 | 0 |
| 720. AN4323.1.NCU04754.1.MG02489.1.FG09512.1 | 0 | 0 | 0 | 1 | 3 | 0 |
| 721. AN4332.1.NCU02468.1.MG03090.1.FG09423.1 | 8 | 0 | 0 | 0 | 1 | 0 |
| 722. AN4342.1.NCU10034.1.MG07181.1.FG04395.1 | 4 | 0 | 0 | 0 | 1 | 0 |
| 723. AN4349.1.NCU01659.1.MG01106.1.FG06284.1 | 4 | 0 | 0 | 0 | 3 | 0 |
| 724. AN4353.1.NCU03372.1.MG02409.1.FG10853.1 | 0 | 0 | 1 | 3 | 0 | 0 |
| 725. AN4376.1.NCU01195.1.MG08074.1.FG07174.1 | 4 | 2 | 0 | 0 | 2 | 0 |
| Ortholog | Totally Conserved Introns | Gained Introns | Lost Introns | Other Introns | Fail/Homology Positions | Fail/Nearby Intron Positions |
| 726. AN4378.1.NCU04310.1.MG01811.1.FG10028.1 | 0 | 2 | 0 | 1 | 2 | 0 |
| 727. AN4382.1.NCU04309.1.MG02303.1.FG10322.1 | 0 | 1 | 0 | 0 | 3 | 0 |
| 728. AN4385.1.NCU01335.1.MG04100.1.FG10381.1 | 4 | 0 | 2 | 6 | 15 | 0 |
| 729. AN4386.1.NCU04259.1.MG00957.1.FG09938.1 | 0 | 0 | 0 | 2 | 7 | 0 |
| 730. AN4388.1.NCU08940.1.MG06192.1.FG10293.1 | 4 | 0 | 0 | 0 | 2 | 0 |
| 731. AN4395.1.NCU08939.1.MG06191.1.FG10091.1 | 8 | 0 | 1 | 5 | 3 | 0 |
| 732. AN4397.1.NCU08935.1.MG06199.1.FG10362.1 | 0 | 0 | 3 | 8 | 3 | 0 |
| 733. AN4399.1.NCU04302.1.MG00970.1.FG09935.1 | 4 | 0 | 0 | 0 | 3 | 0 |
| 734. AN4401.1.NCU04303.1.MG00969.1.FG09934.1 | 20 | 1 | 2 | 7 | 0 | 0 |
| 735. AN4404.1.NCU04306.1.MG00966.1.FG10320.1 | 0 | 0 | 0 | 1 | 7 | 0 |
| 736. AN4409.1.NCU01667.1.MG01102.1.FG06281.1 | 0 | 0 | 0 | 6 | 1 | 0 |
| 737. AN4412.1.NCU03363.1.MG03931.1.FG01656.1 | 4 | 0 | 0 | 2 | 1 | 0 |
| 738. AN4413.1.NCU01486.1.MG07174.1.FG04361.1 | 0 | 0 | 1 | 7 | 8 | 0 |
| 739. AN4417.1.NCU06776.1.MG03886.1.FG10865.1 | 0 | 0 | 0 | 3 | 3 | 0 |
| 740. AN4419.1.NCU08158.1.MG09700.1.FG10516.1 | 0 | 0 | 0 | 1 | 3 | 0 |
| 741. AN4428.1.NCU01481.1.MG07178.1.FG04398.1 | 8 | 0 | 0 | 2 | 2 | 0 |
| 742. AN4430.1.NCU01666.1.MG01104.1.FG06282.1 | 4 | 0 | 0 | 0 | 0 | 0 |
| 743. AN4435.1.NCU03360.1.MG03232.1.FG00627.1 | 4 | 0 | 0 | 0 | 2 | 0 |
| 744. AN4441.1.NCU03335.1.MG03907.1.FG01620.1 | 0 | 0 | 0 | 1 | 2 | 0 |
| 745. AN4443.1.NCU06512.1.MG06712.1.FG10825.1 | 0 | 0 | 1 | 5 | 1 | 0 |
| 746. AN4446.1.NCU04003.1.MG08096.1.FG10067.1 | 4 | 0 | 0 | 4 | 0 | 0 |
| 747. AN4449.1.NCU03304.1.MG04551.1.FG01236.1 | 4 | 0 | 0 | 0 | 3 | 0 |
| 748. AN4456.1.NCU01367.1.MG07030.1.FG06802.1 | 0 | 0 | 0 | 1 | 0 | 0 |
| 749. AN4460.1.NCU03628.1.MG08178.1.FG06788.1 | 0 | 0 | 2 | 6 | 0 | 0 |
| 750. AN4463.1.NCU02510.1.MG07768.1.FG05619.1 | 8 | 0 | 1 | 3 | 2 | 0 |
| Ortholog | Totally Conserved Introns | Gained Introns | Lost Introns | Other Introns | Fail/Homology Positions | Fail/Nearby Intron Positions |
| 751. AN4464.1.NCU02629.1.MG04435.1.FG00969.1 | 4 | 0 | 0 | 0 | 2 | 0 |
| 752. AN4468.1.NCU09539.1.MG09529.1.FG07438.1 | 4 | 0 | 0 | 0 | 5 | 0 |
| 753. AN4469.1.NCU03764.1.MG02460.1.FG06821.1 | 0 | 0 | 1 | 3 | 1 | 0 |
| 754. AN4470.1.NCU02810.1.MG01592.1.FG06843.1 | 4 | 0 | 0 | 0 | 1 | 0 |
| 755. AN4475.1.NCU02509.1.MG07753.1.FG01081.1 | 4 | 1 | 0 | 0 | 5 | 0 |
| 756. AN4483.1.NCU09212.1.MG08547.1.FG00786.1 | 4 | 0 | 0 | 0 | 6 | 0 |
| 757. AN4486.1.NCU02094.1.MG00672.1.FG00719.1 | 4 | 0 | 0 | 1 | 4 | 0 |
| 758. AN4490.1.NCU02943.1.MG04219.1.FG09359.1 | 4 | 0 | 0 | 0 | 2 | 0 |
| 759. AN4492.1.NCU00823.1.MG05858.1.FG00781.1 | 4 | 0 | 0 | 1 | 4 | 0 |
| 760. AN4501.1.NCU02806.1.MG01588.1.FG06847.1 | 12 | 0 | 1 | 3 | 0 | 0 |
| 761. AN4515.1.NCU05686.1.MG01134.1.FG02720.1 | 4 | 0 | 0 | 0 | 1 | 0 |
| 762. AN4517.1.NCU00010.1.MG05190.1.FG05527.1 | 0 | 0 | 0 | 4 | 1 | 0 |
| 763. AN4522.1.NCU07857.1.MG05296.1.FG04149.1 | 4 | 0 | 0 | 0 | 3 | 0 |
| 764. AN4536.1.NCU06249.1.MG05220.1.FG06970.1 | 0 | 1 | 0 | 0 | 5 | 0 |
| 765. AN4547.1.NCU01992.1.MG02634.1.FG08840.1 | 8 | 0 | 0 | 1 | 2 | 0 |
| 766. AN4549.1.NCU03173.1.MG03021.1.FG09541.1 | 4 | 0 | 0 | 0 | 3 | 0 |
| 767. AN4556.1.NCU00080.1.MG10154.1.FG06717.1 | 0 | 0 | 1 | 2 | 2 | 0 |
| 768. AN4557.1.NCU01479.1.MG07441.1.FG06816.1 | 4 | 0 | 0 | 0 | 0 | 0 |
| 769. AN4558.1.NCU01478.1.MG07450.1.FG06810.1 | 4 | 0 | 0 | 2 | 6 | 0 |
| 770. AN4559.1.NCU00075.1.MG10159.1.FG06723.1 | 8 | 0 | 0 | 1 | 0 | 0 |
| 771. AN4563.1.NCU00685.1.MG02829.1.FG08731.1 | 0 | 1 | 0 | 0 | 5 | 0 |
| 772. AN4564.1.NCU00622.1.MG02875.1.FG08703.1 | 8 | 0 | 0 | 0 | 2 | 0 |
| 773. AN4566.1.NCU04251.1.MG09551.1.FG10327.1 | 0 | 1 | 0 | 5 | 4 | 0 |
| 774. AN4576.1.NCU08315.1.MG04166.1.FG05063.1 | 0 | 0 | 2 | 2 | 1 | 0 |
| 775. AN4577.1.NCU02361.1.MG03533.1.FG09042.1 | 0 | 8 | 3 | 6 | 5 | 0 |
| Ortholog | Totally Conserved Introns | Gained Introns | Lost Introns | Other Introns | Fail/Homology Positions | Fail/Nearby Intron Positions |
| 776. AN4578.1.NCU02360.1.MG03534.1.FG07966.1 | 8 | 2 | 0 | 1 | 1 | 0 |
| 777. AN4583.1.NCU03853.1.MG08104.1.FG10352.1 | 0 | 0 | 0 | 4 | 1 | 2 |
| 778. AN4589.1.NCU04127.1.MG02742.1.FG08871.1 | 8 | 2 | 0 | 0 | 0 | 0 |
| 779. AN4602.1.NCU02264.1.MG00904.1.FG06294.1 | 4 | 0 | 2 | 4 | 3 | 0 |
| 780. AN4603.1.NCU02296.1.MG10412.1.FG10934.1 | 0 | 0 | 0 | 2 | 0 | 0 |
| 781. AN4611.1.NCU00564.1.MG03019.1.FG00168.1 | 8 | 0 | 0 | 0 | 0 | 0 |
| 782. AN4616.1.NCU00692.1.MG02842.1.FG08644.1 | 0 | 0 | 1 | 4 | 2 | 0 |
| 783. AN4623.1.NCU02349.1.MG03510.1.FG07940.1 | 4 | 1 | 0 | 0 | 3 | 0 |
| 784. AN4625.1.NCU05616.1.MG08029.1.FG04845.1 | 4 | 0 | 0 | 0 | 4 | 0 |
| 785. AN4639.1.NCU00621.1.MG02874.1.FG08704.1 | 4 | 0 | 0 | 1 | 8 | 0 |
| 786. AN4645.1.NCU01356.1.MG09545.1.FG10018.1 | 0 | 1 | 0 | 0 | 2 | 0 |
| 787. AN4647.1.NCU04228.1.MG06880.1.FG10257.1 | 0 | 1 | 0 | 0 | 0 | 0 |
| 788. AN4655.1.NCU05778.1.MG01170.1.FG02624.1 | 0 | 2 | 0 | 1 | 2 | 0 |
| 789. AN4663.1.NCU04698.1.MG01296.1.FG09525.1 | 8 | 0 | 0 | 0 | 0 | 0 |
| 790. AN4664.1.NCU09982.1.MG03075.1.FG09418.1 | 0 | 0 | 1 | 3 | 6 | 0 |
| 791. AN4666.1.NCU02466.1.MG03088.1.FG09420.1 | 0 | 0 | 2 | 2 | 4 | 0 |
| 792. AN4667.1.NCU02464.1.MG03087.1.FG09421.1 | 0 | 0 | 0 | 3 | 8 | 0 |
| 793. AN4673.1.NCU09173.1.MG04336.1.FG05679.1 | 4 | 0 | 0 | 0 | 1 | 0 |
| 794. AN4678.1.NCU01936.1.MG02924.1.FG08914.1 | 0 | 1 | 0 | 3 | 0 | 0 |
| 795. AN4683.1.NCU00669.1.MG02821.1.FG00189.1 | 0 | 0 | 1 | 3 | 2 | 0 |
| 796. AN4684.1.NCU02148.1.MG02622.1.FG08571.1 | 0 | 0 | 0 | 3 | 3 | 0 |
| 797. AN4687.1.NCU02127.1.MG02541.1.FG08509.1 | 0 | 0 | 1 | 6 | 4 | 0 |
| 798. AN4688.1.NCU02126.1.MG02540.1.FG08510.1 | 0 | 0 | 1 | 7 | 0 | 0 |
| 799. AN4690.1.NCU00591.1.MG10320.1.FG08688.1 | 0 | 0 | 0 | 3 | 3 | 0 |
| 800. AN4691.1.NCU02128.1.MG02544.1.FG08511.1 | 0 | 2 | 0 | 2 | 6 | 0 |
| Ortholog | Totally Conserved Introns | Gained Introns | Lost Introns | Other Introns | Fail/Homology Positions | Fail/Nearby Intron Positions |
| 801. AN4702.1.NCU00881.1.MG10586.1.FG02810.1 | 4 | 0 | 0 | 0 | 1 | 0 |
| 802. AN4706.1.NCU00551.1.MG03060.1.FG08719.1 | 4 | 0 | 0 | 2 | 2 | 0 |
| 803. AN4707.1.NCU00664.1.MG02820.1.FG00188.1 | 4 | 0 | 0 | 0 | 0 | 0 |
| 804. AN4709.1.NCU00656.1.MG03069.1.FG08522.1 | 0 | 1 | 0 | 0 | 7 | 0 |
| 805. AN4714.1.NCU00605.1.MG02856.1.FG09413.1 | 0 | 0 | 0 | 3 | 1 | 0 |
| 806. AN4717.1.NCU00682.1.MG02832.1.FG08729.1 | 4 | 0 | 2 | 3 | 2 | 0 |
| 807. AN4718.1.NCU00667.1.MG03065.1.FG08573.1 | 12 | 0 | 0 | 1 | 4 | 0 |
| 808. AN4727.1.NCU04442.1.MG08012.1.FG05689.1 | 8 | 0 | 0 | 0 | 2 | 0 |
| 809. AN4728.1.NCU00614.1.MG02867.1.FG00261.1 | 4 | 0 | 0 | 1 | 1 | 0 |
| 810. AN4736.1.NCU01961.1.MG02980.1.FG08889.1 | 0 | 0 | 0 | 1 | 5 | 0 |
| 811. AN4759.1.NCU01911.1.MG02923.1.FG08936.1 | 0 | 0 | 0 | 1 | 1 | 0 |
| 812. AN4761.1.NCU01912.1.MG02954.1.FG08935.1 | 8 | 0 | 1 | 3 | 3 | 0 |
| 813. AN4762.1.NCU02016.1.MG03663.1.FG08530.1 | 0 | 0 | 1 | 7 | 1 | 0 |
| 814. AN4763.1.NCU02015.1.MG03664.1.FG08531.1 | 4 | 0 | 0 | 0 | 1 | 0 |
| 815. AN4769.1.NCU01985.1.MG02643.1.FG08875.1 | 0 | 0 | 3 | 9 | 4 | 0 |
| 816. AN4775.1.NCU02650.1.MG02608.1.FG09432.1 | 4 | 0 | 2 | 7 | 1 | 0 |
| 817. AN4777.1.NCU00618.1.MG02872.1.FG06407.1 | 4 | 0 | 0 | 0 | 1 | 0 |
| 818. AN4778.1.NCU01993.1.MG02633.1.FG08706.1 | 4 | 0 | 1 | 7 | 0 | 0 |
| 819. AN4780.1.NCU00590.1.MG10319.1.FG08689.1 | 0 | 0 | 0 | 3 | 1 | 0 |
| 820. AN4782.1.NCU00600.1.MG10323.1.FG00170.1 | 4 | 0 | 1 | 9 | 1 | 0 |
| 821. AN4793.1.NCU00554.1.MG03051.1.FG08498.1 | 4 | 0 | 0 | 5 | 1 | 0 |
| 822. AN4803.1.NCU01949.1.MG02952.1.FG08896.1 | 8 | 1 | 0 | 1 | 1 | 0 |
| 823. AN4804.1.NCU01971.1.MG02950.1.FG08501.1 | 0 | 0 | 0 | 1 | 3 | 0 |
| 824. AN4806.1.NCU04061.1.MG01293.1.FG11045.1 | 0 | 0 | 1 | 7 | 1 | 0 |
| 825. AN4817.1.NCU01968.1.MG10131.1.FG08672.1 | 0 | 0 | 0 | 1 | 3 | 0 |
| Ortholog | Totally Conserved Introns | Gained Introns | Lost Introns | Other Introns | Fail/Homology Positions | Fail/Nearby Intron Positions |
| 826. AN4830.1.NCU09090.1.MG02725.1.FG08803.1 | 0 | 3 | 0 | 0 | 1 | 0 |
| 827. AN4842.1.NCU03177.1.MG03006.1.FG09542.1 | 0 | 0 | 1 | 4 | 2 | 0 |
| 828. AN4854.1.NCU04193.1.MG10057.1.FG04161.1 | 0 | 0 | 1 | 6 | 0 | 0 |
| 829. AN4857.1.NCU01997.1.MG02627.1.FG08709.1 | 4 | 0 | 0 | 0 | 2 | 0 |
| 830. AN4862.1.NCU03207.1.MG01248.1.FG00562.1 | 0 | 0 | 0 | 3 | 0 | 0 |
| 831. AN4864.1.NCU03317.1.MG09310.1.FG05607.1 | 4 | 0 | 0 | 1 | 0 | 0 |
| 832. AN4869.1.NCU10061.1.MG01153.1.FG10255.1 | 8 | 0 | 0 | 0 | 3 | 1 |
| 833. AN4870.1.NCU08972.1.MG01152.1.FG10254.1 | 0 | 0 | 0 | 2 | 3 | 0 |
| 834. AN4871.1.NCU04554.1.MG07927.1.FG10939.1 | 0 | 0 | 0 | 6 | 5 | 0 |
| 835. AN4876.1.NCU05902.1.MG03313.1.FG06075.1 | 4 | 0 | 1 | 3 | 3 | 0 |
| 836. AN4883.1.NCU09706.1.MG03854.1.FG06316.1 | 0 | 0 | 1 | 2 | 4 | 0 |
| 837. AN4884.1.NCU02270.1.MG00918.1.FG06301.1 | 0 | 0 | 0 | 2 | 7 | 0 |
| 838. AN4890.1.NCU09432.1.MG00926.1.FG00343.1 | 0 | 0 | 1 | 8 | 7 | 0 |
| 839. AN4892.1.NCU09435.1.MG03265.1.FG00349.1 | 4 | 0 | 1 | 5 | 5 | 0 |
| 840. AN4900.1.NCU00223.1.MG05306.1.FG06664.1 | 4 | 0 | 1 | 3 | 3 | 0 |
| 841. AN4905.1.NCU04109.1.MG06747.1.FG02000.1 | 0 | 0 | 1 | 3 | 2 | 1 |
| 842. AN4913.1.NCU05151.1.MG09116.1.FG06553.1 | 0 | 1 | 1 | 7 | 3 | 0 |
| 843. AN4915.1.NCU06410.1.MG06241.1.FG05501.1 | 8 | 0 | 0 | 0 | 0 | 0 |
| 844. AN4916.1.NCU00258.1.MG00221.1.FG06587.1 | 8 | 0 | 0 | 0 | 0 | 0 |
| 845. AN4917.1.NCU00257.1.MG00222.1.FG06588.1 | 4 | 0 | 0 | 0 | 3 | 0 |
| 846. AN4923.1.NCU03922.1.MG01026.1.FG09266.1 | 4 | 0 | 0 | 3 | 1 | 0 |
| 847. AN4939.1.NCU07286.1.MG00274.1.FG02758.1 | 0 | 0 | 0 | 1 | 4 | 0 |
| 848. AN4940.1.NCU04415.1.MG05164.1.FG11602.1 | 4 | 2 | 1 | 5 | 1 | 0 |
| 849. AN4942.1.NCU04759.1.MG02479.1.FG08477.1 | 4 | 0 | 0 | 0 | 4 | 0 |
| 850. AN4947.1.NCU07965.1.MG05077.1.FG04044.1 | 4 | 1 | 0 | 0 | 0 | 0 |
| Ortholog | Totally Conserved Introns | Gained Introns | Lost Introns | Other Introns | Fail/Homology Positions | Fail/Nearby Intron Positions |
| 851. AN4951.1.NCU06284.1.MG05089.1.FG02756.1 | 4 | 0 | 0 | 5 | 1 | 0 |
| 852. AN4956.1.NCU07982.1.MG06868.1.FG01086.1 | 0 | 3 | 1 | 3 | 4 | 0 |
| 853. AN4957.1.NCU08687.1.MG05440.1.FG04085.1 | 4 | 0 | 0 | 0 | 3 | 0 |
| 854. AN4965.1.NCU04766.1.MG02476.1.FG08474.1 | 4 | 0 | 0 | 0 | 3 | 0 |
| 855. AN4967.1.NCU04743.1.MG02486.1.FG09516.1 | 4 | 0 | 0 | 1 | 4 | 0 |
| 856. AN4969.1.NCU03899.1.MG01027.1.FG09262.1 | 0 | 1 | 0 | 3 | 1 | 0 |
| 857. AN4975.1.NCU04771.1.MG08877.1.FG08428.1 | 0 | 1 | 0 | 1 | 1 | 0 |
| 858. AN4977.1.NCU04768.1.MG08880.1.FG08425.1 | 0 | 0 | 2 | 2 | 1 | 0 |
| 859. AN4986.1.NCU02176.1.MG02749.1.FG08717.1 | 4 | 0 | 0 | 0 | 3 | 0 |
| 860. AN4987.1.NCU01166.1.MG07335.1.FG09908.1 | 0 | 0 | 0 | 3 | 0 | 0 |
| 861. AN4992.1.NCU02416.1.MG03560.1.FG06375.1 | 0 | 0 | 0 | 1 | 1 | 0 |
| 862. AN4993.1.NCU02417.1.MG03561.1.FG06376.1 | 0 | 0 | 0 | 1 | 4 | 0 |
| 863. AN4997.1.NCU02263.1.MG00905.1.FG10779.1 | 8 | 0 | 0 | 3 | 0 | 0 |
| 864. AN4998.1.NCU06122.1.MG03846.1.FG00355.1 | 8 | 0 | 0 | 3 | 1 | 0 |
| 865. AN5008.1.NCU02293.1.MG06573.1.FG10793.1 | 0 | 0 | 1 | 3 | 0 | 0 |
| 866. AN5010.1.NCU05894.1.MG03271.1.FG06070.1 | 4 | 0 | 0 | 0 | 1 | 0 |
| 867. AN5011.1.NCU05895.1.MG03376.1.FG06069.1 | 4 | 0 | 0 | 4 | 9 | 0 |
| 868. AN5014.1.NCU06661.1.MG04104.1.FG09974.1 | 0 | 0 | 1 | 2 | 4 | 0 |
| 869. AN5019.1.NCU08434.1.MG05888.1.FG07880.1 | 4 | 1 | 0 | 1 | 1 | 0 |
| 870. AN5020.1.NCU07173.1.MG10676.1.FG04483.1 | 4 | 0 | 1 | 5 | 0 | 0 |
| 871. AN5021.1.NCU09559.1.MG00195.1.FG04456.1 | 0 | 0 | 1 | 6 | 7 | 0 |
| 872. AN5022.1.NCU07196.1.MG08423.1.FG10315.1 | 0 | 0 | 1 | 5 | 0 | 0 |
| 873. AN5049.1.NCU09368.1.MG04623.1.FG01162.1 | 4 | 1 | 0 | 5 | 3 | 0 |
| 874. AN5057.1.NCU03246.1.MG04637.1.FG00543.1 | 4 | 0 | 0 | 0 | 3 | 0 |
| 875. AN5101.1.NCU07200.1.MG08041.1.FG00028.1 | 0 | 0 | 1 | 2 | 2 | 0 |
| Ortholog | Totally Conserved Introns | Gained Introns | Lost Introns | Other Introns | Fail/Homology Positions | Fail/Nearby Intron Positions |
| 876. AN5102.1.NCU01164.1.MG07316.1.FG10040.1 | 8 | 0 | 0 | 2 | 3 | 1 |
| 877. AN5109.1.NCU01759.1.MG03160.1.FG06240.1 | 0 | 0 | 0 | 1 | 3 | 0 |
| 878. AN5110.1.NCU01758.1.MG03159.1.FG05197.1 | 0 | 1 | 0 | 1 | 3 | 0 |
| 879. AN5121.1.NCU01547.1.MG01059.1.FG10738.1 | 4 | 0 | 0 | 0 | 2 | 0 |
| 880. AN5122.1.NCU01550.1.MG01058.1.FG10737.1 | 8 | 0 | 1 | 3 | 0 | 0 |
| 881. AN5123.1.NCU01704.1.MG08569.1.FG00320.1 | 4 | 0 | 0 | 2 | 1 | 0 |
| 882. AN5127.1.NCU01741.1.MG07189.1.FG01435.1 | 0 | 0 | 2 | 2 | 3 | 0 |
| 883. AN5129.1.NCU09602.1.MG06958.2.FG00838.1 | 0 | 4 | 0 | 0 | 0 | 2 |
| 884. AN5132.1.NCU03561.1.MG03224.1.FG00646.1 | 4 | 1 | 3 | 8 | 1 | 0 |
| 885. AN5134.1.NCU01744.1.MG07187.1.FG01433.1 | 4 | 1 | 3 | 9 | 1 | 0 |
| 886. AN5139.1.NCU08484.1.MG06969.1.FG10869.1 | 4 | 0 | 0 | 2 | 6 | 0 |
| 887. AN5140.1.NCU09098.1.MG02722.1.FG08810.1 | 4 | 0 | 0 | 3 | 1 | 0 |
| 888. AN5146.1.NCU07362.1.MG00335.1.FG01027.1 | 12 | 0 | 0 | 1 | 0 | 0 |
| 889. AN5152.1.NCU01814.1.MG06689.1.FG06003.1 | 4 | 0 | 0 | 1 | 6 | 0 |
| 890. AN5162.1.NCU06482.1.MG06371.1.FG05454.1 | 8 | 1 | 0 | 0 | 1 | 0 |
| 891. AN5166.1.NCU09643.1.MG09371.1.FG04237.1 | 0 | 1 | 1 | 3 | 3 | 0 |
| 892. AN5167.1.NCU07565.1.MG00134.1.FG06762.1 | 0 | 0 | 3 | 5 | 3 | 0 |
| 893. AN5181.1.NCU02588.1.MG03604.1.FG09165.1 | 0 | 0 | 0 | 1 | 4 | 0 |
| 894. AN5187.1.NCU01724.1.MG08580.1.FG07860.1 | 0 | 1 | 1 | 2 | 6 | 0 |
| 895. AN5193.1.NCU02543.1.MG01564.1.FG01389.1 | 0 | 1 | 0 | 1 | 1 | 2 |
| 896. AN5194.1.NCU02544.1.MG01563.1.FG01388.1 | 0 | 0 | 0 | 5 | 3 | 0 |
| 897. AN5195.1.NCU06922.1.MG08132.1.FG01398.1 | 8 | 0 | 1 | 3 | 0 | 0 |
| 898. AN5196.1.NCU06921.1.MG08133.1.FG01399.1 | 0 | 2 | 0 | 0 | 2 | 0 |
| 899. AN5199.1.NCU06923.1.MG08139.1.FG01397.1 | 0 | 0 | 1 | 6 | 3 | 0 |
| 900. AN5200.1.NCU06924.1.MG11020.1.FG01395.1 | 0 | 1 | 0 | 0 | 4 | 0 |
| Ortholog | Totally Conserved Introns | Gained Introns | Lost Introns | Other Introns | Fail/Homology Positions | Fail/Nearby Intron Positions |
| 901. AN5206.1.NCU02954.1.MG01566.1.FG01392.1 | 0 | 0 | 0 | 2 | 0 | 2 |
| 902. AN5210.1.NCU06075.1.MG08063.1.FG07528.1 | 0 | 2 | 0 | 6 | 1 | 0 |
| 903. AN5217.1.NCU07495.1.MG00153.1.FG00900.1 | 0 | 0 | 2 | 5 | 3 | 0 |
| 904. AN5221.1.NCU03103.1.MG03693.1.FG00672.1 | 0 | 0 | 2 | 2 | 3 | 0 |
| 905. AN5222.1.NCU03102.1.MG03680.1.FG00671.1 | 8 | 1 | 0 | 0 | 0 | 0 |
| 906. AN5230.1.NCU03211.1.MG03679.1.FG00670.1 | 4 | 0 | 0 | 0 | 0 | 0 |
| 907. AN5232.1.NCU04594.1.MG01347.1.FG10039.1 | 0 | 0 | 1 | 2 | 2 | 0 |
| 908. AN5282.1.NCU09680.1.MG05520.1.FG03628.1 | 0 | 4 | 2 | 4 | 8 | 2 |
| 909. AN5287.1.NCU01181.1.MG08661.1.FG05140.1 | 0 | 0 | 1 | 2 | 4 | 0 |
| 910. AN5338.1.NCU07332.1.MG05574.1.FG06539.1 | 0 | 1 | 2 | 5 | 0 | 0 |
| 911. AN5341.1.NCU04379.1.MG01550.1.FG09591.1 | 8 | 0 | 0 | 0 | 1 | 0 |
| 912. AN5343.1.NCU09730.1.MG01757.1.FG05088.1 | 4 | 0 | 0 | 1 | 4 | 0 |
| 913. AN5354.1.NCU06080.1.MG04975.1.FG09186.1 | 0 | 0 | 1 | 3 | 5 | 0 |
| 914. AN5355.1.NCU08402.1.MG00246.1.FG01686.1 | 0 | 0 | 3 | 4 | 0 | 0 |
| 915. AN5404.1.NCU06361.1.MG04197.1.FG09796.1 | 0 | 0 | 0 | 2 | 7 | 0 |
| 916. AN5436.1.NCU05200.1.MG01824.1.FG05095.1 | 0 | 2 | 0 | 6 | 7 | 0 |
| 917. AN5441.1.NCU00475.1.MG05661.1.FG06893.1 | 0 | 3 | 1 | 3 | 5 | 0 |
| 918. AN5442.1.NCU00477.1.MG05663.1.FG06895.1 | 4 | 0 | 0 | 0 | 0 | 0 |
| 919. AN5447.1.NCU06803.1.MG02378.1.FG01572.1 | 4 | 0 | 1 | 3 | 2 | 0 |
| 920. AN5452.1.NCU00396.1.MG06457.1.FG07245.1 | 8 | 0 | 0 | 0 | 5 | 0 |
| 921. AN5455.1.NCU00336.1.MG04995.1.FG07262.1 | 0 | 0 | 1 | 4 | 2 | 0 |
| 922. AN5457.1.NCU02110.1.MG00750.1.FG00739.1 | 0 | 0 | 1 | 6 | 2 | 0 |
| 923. AN5482.1.NCU09269.1.MG09952.1.FG01099.1 | 12 | 0 | 0 | 0 | 3 | 0 |
| 924. AN5486.1.NCU06726.1.MG06196.1.FG10250.1 | 0 | 0 | 0 | 1 | 5 | 0 |
| 925. AN5493.1.NCU08148.1.MG05467.1.FG10531.1 | 0 | 0 | 0 | 2 | 3 | 1 |
| Ortholog | Totally Conserved Introns | Gained Introns | Lost Introns | Other Introns | Fail/Homology Positions | Fail/Nearby Intron Positions |
| 926. AN5497.1.NCU05983.1.MG01049.1.FG08452.1 | 4 | 0 | 0 | 1 | 2 | 0 |
| 927. AN5512.1.NCU07321.1.MG04948.1.FG04986.1 | 0 | 1 | 0 | 0 | 1 | 0 |
| 928. AN5517.1.NCU05939.1.MG08345.1.FG02237.1 | 4 | 0 | 0 | 0 | 2 | 0 |
| 929. AN5519.1.NCU09461.1.MG02434.1.FG06007.1 | 4 | 0 | 0 | 0 | 0 | 0 |
| 930. AN5520.1.NCU04779.1.MG04612.1.FG08478.1 | 8 | 0 | 0 | 0 | 2 | 0 |
| 931. AN5523.1.NCU09715.1.MG03860.1.FG06051.1 | 4 | 0 | 1 | 5 | 5 | 0 |
| 932. AN5525.1.NCU02366.1.MG03521.1.FG07953.1 | 0 | 4 | 1 | 5 | 3 | 0 |
| 933. AN5528.1.NCU05029.1.MG03406.1.FG10911.1 | 0 | 0 | 0 | 3 | 2 | 0 |
| 934. AN5529.1.NCU07296.1.MG05376.1.FG01188.1 | 4 | 0 | 0 | 0 | 3 | 0 |
| 935. AN5530.1.NCU07299.1.MG05375.1.FG01187.1 | 0 | 0 | 0 | 1 | 0 | 0 |
| 936. AN5535.1.NCU04505.1.MG00102.1.FG03976.1 | 0 | 0 | 0 | 1 | 1 | 0 |
| 937. AN5536.1.NCU04507.1.MG00101.1.FG04038.1 | 4 | 0 | 0 | 0 | 0 | 0 |
| 938. AN5563.1.NCU04923.1.MG00097.1.FG07908.1 | 0 | 0 | 0 | 1 | 1 | 0 |
| 939. AN5564.1.NCU04924.1.MG00099.1.FG07907.1 | 0 | 1 | 0 | 0 | 0 | 0 |
| 940. AN5566.1.NCU02325.1.MG00919.1.FG10358.1 | 0 | 10 | 0 | 1 | 10 | 2 |
| 941. AN5574.1.NCU09561.1.MG00213.1.FG04453.1 | 4 | 0 | 0 | 1 | 1 | 0 |
| 942. AN5577.1.NCU09560.1.MG00212.1.FG04454.1 | 4 | 0 | 0 | 1 | 1 | 0 |
| 943. AN5579.1.NCU05837.1.MG06537.1.FG10530.1 | 0 | 0 | 0 | 3 | 1 | 0 |
| 944. AN5586.1.NCU06003.1.MG01288.1.FG08773.1 | 0 | 0 | 0 | 1 | 3 | 0 |
| 945. AN5588.1.NCU03359.1.MG03926.1.FG01475.1 | 4 | 0 | 0 | 0 | 4 | 0 |
| 946. AN5591.1.NCU03500.1.MG03940.1.FG05169.1 | 0 | 8 | 1 | 3 | 4 | 0 |
| 947. AN5592.1.NCU06067.1.MG09697.1.FG10511.1 | 4 | 0 | 1 | 6 | 3 | 0 |
| 948. AN5599.1.NCU05985.1.MG01291.1.FG08766.1 | 4 | 0 | 0 | 2 | 4 | 0 |
| 949. AN5601.1.NCU03748.1.MG08564.1.FG00346.1 | 0 | 6 | 3 | 9 | 4 | 2 |
| 950. AN5602.1.NCU04087.1.MG00814.1.FG09535.1 | 0 | 0 | 1 | 3 | 4 | 0 |
| Ortholog | Totally Conserved Introns | Gained Introns | Lost Introns | Other Introns | Fail/Homology Positions | Fail/Nearby Intron Positions |
| 951. AN5604.1.NCU04797.1.MG08895.1.FG09280.1 | 4 | 0 | 0 | 3 | 0 | 0 |
| 952. AN5606.1.NCU03463.1.MG03947.1.FG00818.1 | 8 | 3 | 0 | 0 | 2 | 0 |
| 953. AN5607.1.NCU05620.1.MG04342.1.FG06417.1 | 4 | 0 | 1 | 3 | 1 | 0 |
| 954. AN5616.1.NCU03347.1.MG03264.1.FG01560.1 | 4 | 1 | 4 | 10 | 2 | 0 |
| 955. AN5626.1.NCU06836.1.MG03201.1.FG00330.1 | 4 | 0 | 2 | 5 | 3 | 0 |
| 956. AN5629.1.NCU04044.1.MG00647.1.FG09250.1 | 4 | 0 | 0 | 4 | 0 | 0 |
| 957. AN5634.1.NCU04230.1.MG04895.1.FG09896.1 | 8 | 0 | 2 | 4 | 0 | 0 |
| 958. AN5635.1.NCU04221.1.MG09471.1.FG09895.1 | 4 | 0 | 0 | 0 | 5 | 0 |
| 959. AN5643.1.NCU03875.1.MG01012.1.FG10269.1 | 0 | 1 | 0 | 1 | 6 | 0 |
| 960. AN5656.1.NCU01411.1.MG09502.1.FG10120.1 | 0 | 4 | 0 | 0 | 4 | 0 |
| 961. AN5662.1.NCU02380.1.MG03551.1.FG01248.1 | 0 | 3 | 1 | 4 | 6 | 0 |
| 962. AN5670.1.NCU02280.1.MG00878.1.FG06337.1 | 4 | 0 | 0 | 0 | 3 | 0 |
| 963. AN5672.1.NCU09034.1.MG08317.1.FG09883.1 | 0 | 1 | 0 | 2 | 4 | 0 |
| 964. AN5673.1.NCU09033.1.MG08314.1.FG09884.1 | 0 | 0 | 0 | 1 | 8 | 0 |
| 965. AN5677.1.NCU09537.1.MG09531.1.FG07433.1 | 4 | 0 | 0 | 4 | 2 | 0 |
| 966. AN5678.1.NCU05830.1.MG11062.1.FG00613.1 | 4 | 0 | 0 | 0 | 2 | 0 |
| 967. AN5686.1.NCU01204.1.MG08078.1.FG07443.1 | 8 | 0 | 0 | 0 | 0 | 0 |
| 968. AN5701.1.NCU05548.1.MG09766.1.FG08338.1 | 4 | 0 | 0 | 3 | 1 | 0 |
| 969. AN5704.1.NCU05008.1.MG03484.1.FG07865.1 | 4 | 0 | 2 | 6 | 3 | 0 |
| 970. AN5705.1.NCU06091.1.MG00839.1.FG06346.1 | 4 | 0 | 1 | 7 | 0 | 0 |
| 971. AN5713.1.NCU09700.1.MG03310.1.FG06306.1 | 8 | 0 | 1 | 5 | 0 | 0 |
| 972. AN5718.1.NCU08959.1.MG06186.1.FG10367.1 | 8 | 0 | 2 | 2 | 5 | 0 |
| 973. AN5719.1.NCU08960.1.MG06185.1.FG10368.1 | 8 | 0 | 0 | 3 | 0 | 0 |
| 974. AN5722.1.NCU01767.1.MG03154.1.FG05205.1 | 4 | 0 | 1 | 3 | 0 | 0 |
| 975. AN5725.1.NCU06779.1.MG03883.1.FG10858.1 | 4 | 0 | 0 | 0 | 4 | 0 |
| Ortholog | Totally Conserved Introns | Gained Introns | Lost Introns | Other Introns | Fail/Homology Positions | Fail/Nearby Intron Positions |
| 976. AN5727.1.NCU00972.1.MG09726.1.FG11048.1 | 0 | 2 | 0 | 1 | 1 | 0 |
| 977. AN5731.1.NCU05420.1.MG03281.1.FG01643.1 | 0 | 1 | 1 | 4 | 0 | 0 |
| 978. AN5732.1.NCU05421.1.MG03282.1.FG01642.1 | 0 | 0 | 1 | 3 | 1 | 0 |
| 979. AN5734.1.NCU00988.1.MG04225.1.FG06235.1 | 0 | 6 | 1 | 7 | 6 | 1 |
| 980. AN5742.1.NCU05897.1.MG09193.1.FG01593.1 | 4 | 1 | 0 | 3 | 2 | 0 |
| 981. AN5746.1.NCU10042.1.MG10607.1.FG01346.1 | 4 | 0 | 1 | 7 | 1 | 0 |
| 982. AN5747.1.NCU07367.1.MG00330.1.FG01198.1 | 8 | 0 | 0 | 0 | 7 | 0 |
| 983. AN5751.1.NCU08151.1.MG09734.1.FG10482.1 | 0 | 0 | 6 | 10 | 12 | 0 |
| 984. AN5753.1.NCU08137.1.MG09743.1.FG10755.1 | 4 | 0 | 0 | 0 | 0 | 0 |
| 985. AN5757.1.NCU04005.1.MG08097.1.FG10066.1 | 8 | 0 | 1 | 4 | 3 | 0 |
| 986. AN5763.1.NCU07960.1.MG05085.1.FG00405.1 | 4 | 1 | 0 | 2 | 11 | 0 |
| 987. AN5768.1.NCU03660.1.MG07482.1.FG07425.1 | 0 | 0 | 0 | 2 | 6 | 0 |
| 988. AN5778.1.NCU02781.1.MG04352.1.FG00610.1 | 4 | 0 | 0 | 1 | 1 | 0 |
| 989. AN5785.1.NCU07956.1.MG05136.1.FG01345.1 | 0 | 5 | 0 | 1 | 3 | 0 |
| 990. AN5786.1.NCU07955.1.MG05135.1.FG01344.1 | 0 | 0 | 1 | 4 | 1 | 0 |
| 991. AN5789.1.NCU00777.1.MG08705.1.FG05728.1 | 4 | 0 | 0 | 0 | 0 | 0 |
| 992. AN5790.1.NCU00775.1.MG01995.1.FG05733.1 | 0 | 0 | 1 | 4 | 5 | 0 |
| 993. AN5793.1.NCU02493.1.MG07727.1.FG00564.1 | 8 | 0 | 0 | 2 | 0 | 0 |
| 994. AN5800.1.NCU03988.1.MG04484.1.FG09866.1 | 12 | 0 | 0 | 0 | 0 | 0 |
| 995. AN5801.1.NCU03989.1.MG04483.1.FG09865.1 | 4 | 0 | 1 | 4 | 6 | 0 |
| 996. AN5808.1.NCU07705.1.MG01734.1.FG05068.1 | 4 | 0 | 0 | 0 | 2 | 0 |
| 997. AN5811.1.NCU08550.1.MG09114.1.FG06524.1 | 0 | 0 | 0 | 3 | 3 | 0 |
| 998. AN5812.1.NCU06732.1.MG09481.1.FG10188.1 | 0 | 0 | 1 | 11 | 7 | 0 |
| 999. AN5815.1.NCU00108.1.MG00479.1.FG06959.1 | 4 | 0 | 0 | 0 | 3 | 0 |
| 1000. AN5817.1.NCU00106.1.MG00481.1.FG06961.1 | 0 | 0 | 0 | 1 | 11 | 0 |
| Ortholog | Totally Conserved Introns | Gained Introns | Lost Introns | Other Introns | Fail/Homology Positions | Fail/Nearby Intron Positions |
| 1001. AN5820.1.NCU08216.1.MG07384.1.FG06544.1 | 4 | 0 | 0 | 1 | 1 | 0 |
| 1002. AN5823.1.NCU07117.1.MG04212.1.FG05371.1 | 4 | 0 | 0 | 0 | 2 | 0 |
| 1003. AN5832.1.NCU03616.1.MG09499.1.FG10114.1 | 0 | 0 | 0 | 4 | 1 | 0 |
| 1004. AN5833.1.NCU01417.1.MG00689.1.FG10126.1 | 0 | 2 | 0 | 1 | 6 | 0 |
| 1005. AN5839.1.NCU05802.1.MG04709.1.FG09875.1 | 0 | 1 | 2 | 10 | 5 | 1 |
| 1006. AN5840.1.NCU05803.1.MG04710.1.FG09874.1 | 4 | 0 | 0 | 2 | 11 | 0 |
| 1007. AN5856.1.NCU03312.1.MG09884.1.FG00371.1 | 0 | 0 | 2 | 6 | 1 | 0 |
| 1008. AN5865.1.NCU05289.1.MG07136.1.FG06165.1 | 4 | 10 | 0 | 4 | 0 | 0 |
| 1009. AN5872.1.NCU05295.1.MG07130.1.FG06159.1 | 0 | 0 | 1 | 8 | 1 | 0 |
| 1010. AN5873.1.NCU05296.1.MG07129.1.FG06158.1 | 4 | 0 | 0 | 0 | 5 | 0 |
| 1011. AN5875.1.NCU07041.1.MG06049.1.FG06203.1 | 0 | 3 | 0 | 1 | 2 | 0 |
| 1012. AN5880.1.NCU04387.1.MG01556.1.FG09587.1 | 8 | 0 | 0 | 0 | 1 | 2 |
| 1013. AN5883.1.NCU07690.1.MG01728.1.FG09572.1 | 4 | 0 | 0 | 1 | 0 | 0 |
| 1014. AN5886.1.NCU04385.1.MG01553.1.FG09589.1 | 8 | 0 | 1 | 3 | 0 | 0 |
| 1015. AN5895.1.NCU05288.1.MG07137.1.FG06166.1 | 8 | 0 | 3 | 16 | 0 | 0 |
| 1016. AN5899.1.NCU07679.1.MG07098.1.FG05105.1 | 8 | 0 | 1 | 6 | 1 | 0 |
| 1017. AN5901.1.NCU06322.1.MG01917.1.FG05247.1 | 0 | 0 | 1 | 4 | 0 | 0 |
| 1018. AN5912.1.NCU08989.1.MG04976.1.FG06920.1 | 4 | 0 | 1 | 5 | 2 | 0 |
| 1019. AN5914.1.NCU03032.1.MG01330.1.FG00742.1 | 4 | 0 | 0 | 1 | 9 | 0 |
| 1020. AN5915.1.NCU06447.1.MG06273.1.FG07288.1 | 4 | 0 | 0 | 0 | 3 | 0 |
| 1021. AN5925.1.NCU06365.1.MG06067.1.FG01945.1 | 0 | 0 | 1 | 2 | 13 | 0 |
| 1022. AN5932.1.NCU07870.1.MG06397.1.FG05421.1 | 0 | 0 | 1 | 5 | 17 | 0 |
| 1023. AN5935.1.NCU07375.1.MG00346.1.FG01311.1 | 0 | 3 | 0 | 3 | 3 | 0 |
| 1024. AN5950.1.NCU05232.1.MG04150.1.FG09735.1 | 4 | 0 | 1 | 4 | 3 | 0 |
| 1025. AN5954.1.NCU06279.1.MG05266.1.FG02571.1 | 4 | 0 | 0 | 3 | 0 | 0 |
| Ortholog | Totally Conserved Introns | Gained Introns | Lost Introns | Other Introns | Fail/Homology Positions | Fail/Nearby Intron Positions |
| 1026. AN5957.1.NCU04292.1.MG00942.1.FG04570.1 | 4 | 0 | 0 | 1 | 1 | 0 |
| 1027. AN5959.1.NCU00468.1.MG05276.1.FG04139.1 | 0 | 0 | 0 | 2 | 12 | 0 |
| 1028. AN5960.1.NCU07830.1.MG05238.1.FG02541.1 | 4 | 1 | 2 | 6 | 0 | 0 |
| 1029. AN5971.1.NCU05299.1.MG06030.1.FG01941.1 | 4 | 0 | 0 | 0 | 1 | 0 |
| 1030. AN5973.1.NCU07280.1.MG06599.1.FG05845.1 | 4 | 0 | 0 | 2 | 4 | 0 |
| 1031. AN5979.1.NCU07014.1.MG04114.1.FG05640.1 | 0 | 1 | 1 | 4 | 2 | 0 |
| 1032. AN5986.1.NCU01906.1.MG02921.1.FG08941.1 | 0 | 0 | 0 | 8 | 3 | 0 |
| 1033. AN5996.1.NCU00979.1.MG10827.1.FG05605.1 | 4 | 0 | 0 | 2 | 0 | 0 |
| 1034. AN6004.1.NCU05488.1.MG00444.1.FG08365.1 | 4 | 0 | 0 | 1 | 0 | 0 |
| 1035. AN6006.1.NCU01939.1.MG02927.1.FG08910.1 | 12 | 1 | 0 | 2 | 3 | 0 |
| 1036. AN6013.1.NCU04381.1.MG01552.1.FG09590.1 | 4 | 0 | 0 | 0 | 2 | 0 |
| 1037. AN6033.1.NCU08811.1.MG01472.1.FG09712.1 | 8 | 0 | 0 | 0 | 3 | 0 |
| 1038. AN6040.1.NCU06966.1.MG01909.1.FG05666.1 | 4 | 0 | 0 | 0 | 1 | 0 |
| 1039. AN6045.1.NCU07851.1.MG05281.1.FG04124.1 | 0 | 0 | 2 | 6 | 1 | 0 |
| 1040. AN6046.1.NCU07850.1.MG05280.1.FG04123.1 | 8 | 0 | 0 | 0 | 4 | 0 |
| 1041. AN6048.1.NCU07941.1.MG04156.1.FG09739.1 | 4 | 0 | 0 | 4 | 0 | 0 |
| 1042. AN6066.1.NCU00166.1.MG05741.1.FG07259.1 | 4 | 0 | 0 | 0 | 3 | 0 |
| 1043. AN6067.1.NCU00366.1.MG08866.1.FG05425.1 | 0 | 0 | 1 | 2 | 0 | 0 |
| 1044. AN6073.1.NCU03310.1.MG09886.1.FG01119.1 | 8 | 0 | 0 | 1 | 0 | 0 |
| 1045. AN6077.1.NCU01169.1.MG07301.1.FG10231.1 | 8 | 0 | 0 | 0 | 0 | 0 |
| 1046. AN6080.1.NCU02538.1.MG09294.1.FG00505.1 | 0 | 1 | 1 | 3 | 3 | 0 |
| 1047. AN6083.1.NCU08964.1.MG03136.1.FG10246.1 | 4 | 0 | 0 | 3 | 1 | 0 |
| 1048. AN6087.1.NCU07478.1.MG03631.1.FG09323.1 | 0 | 0 | 1 | 3 | 0 | 0 |
| 1049. AN6089.1.NCU01589.1.MG03165.1.FG06246.1 | 4 | 0 | 0 | 0 | 2 | 0 |
| 1050. AN6095.1.NCU01231.1.MG03360.1.FG05882.1 | 0 | 0 | 0 | 6 | 2 | 1 |
| Ortholog | Totally Conserved Introns | Gained Introns | Lost Introns | Other Introns | Fail/Homology Positions | Fail/Nearby Intron Positions |
| 1051. AN6111.1.NCU00334.1.MG08860.1.FG05509.1 | 4 | 0 | 0 | 0 | 2 | 0 |
| 1052. AN6114.1.NCU01321.1.MG06908.1.FG01896.1 | 0 | 1 | 1 | 2 | 0 | 0 |
| 1053. AN6119.1.NCU01464.1.MG06906.1.FG01898.1 | 0 | 2 | 0 | 0 | 1 | 0 |
| 1054. AN6126.1.NCU08535.1.MG07613.1.FG06580.1 | 4 | 1 | 0 | 1 | 1 | 0 |
| 1055. AN6139.1.NCU00168.1.MG11040.1.FG06642.1 | 4 | 0 | 0 | 0 | 0 | 0 |
| 1056. AN6145.1.NCU08554.1.MG07389.1.FG06532.1 | 0 | 0 | 1 | 3 | 3 | 0 |
| 1057. AN6146.1.NCU08552.1.MG07392.1.FG06530.1 | 4 | 0 | 0 | 0 | 3 | 0 |
| 1058. AN6158.1.NCU01320.1.MG06907.1.FG01897.1 | 0 | 0 | 1 | 2 | 3 | 0 |
| 1059. AN6162.1.NCU00034.1.MG05331.1.FG06689.1 | 0 | 0 | 0 | 1 | 0 | 0 |
| 1060. AN6166.1.NCU01322.1.MG06909.1.FG01903.1 | 0 | 0 | 0 | 4 | 0 | 0 |
| 1061. AN6171.1.NCU01008.1.MG04201.1.FG10219.1 | 0 | 0 | 2 | 3 | 2 | 2 |
| 1062. AN6175.1.NCU03307.1.MG09889.1.FG00511.1 | 0 | 0 | 0 | 1 | 0 | 0 |
| 1063. AN6177.1.NCU05899.1.MG00835.1.FG01705.1 | 0 | 2 | 0 | 1 | 4 | 0 |
| 1064. AN6179.1.NCU03309.1.MG09887.1.FG01118.1 | 8 | 0 | 0 | 0 | 1 | 0 |
| 1065. AN6181.1.NCU00706.1.MG00546.1.FG10181.1 | 0 | 1 | 0 | 1 | 2 | 0 |
| 1066. AN6182.1.NCU04460.1.MG05098.1.FG05668.1 | 0 | 0 | 1 | 5 | 4 | 0 |
| 1067. AN6186.1.NCU06585.1.MG01699.1.FG05269.1 | 0 | 2 | 0 | 0 | 0 | 0 |
| 1068. AN6209.1.NCU06187.1.MG03645.1.FG09185.1 | 0 | 5 | 0 | 4 | 7 | 0 |
| 1069. AN6210.1.NCU08012.1.MG01760.1.FG01919.1 | 0 | 0 | 0 | 2 | 3 | 0 |
| 1070. AN6211.1.NCU08298.1.MG01523.1.FG01969.1 | 0 | 1 | 0 | 0 | 1 | 0 |
| 1071. AN6217.1.NCU06483.1.MG06372.1.FG05455.1 | 8 | 0 | 0 | 6 | 1 | 0 |
| 1072. AN6224.1.NCU05312.1.MG06076.1.FG05313.1 | 0 | 1 | 1 | 3 | 1 | 0 |
| 1073. AN6225.1.NCU05313.1.MG06075.1.FG05314.1 | 12 | 0 | 0 | 0 | 0 | 0 |
| 1074. AN6232.1.NCU08515.1.MG03244.1.FG00637.1 | 20 | 0 | 1 | 5 | 0 | 0 |
| 1075. AN6248.1.NCU01516.1.MG03214.1.FG00305.1 | 0 | 0 | 0 | 1 | 1 | 0 |
| Ortholog | Totally Conserved Introns | Gained Introns | Lost Introns | Other Introns | Fail/Homology Positions | Fail/Nearby Intron Positions |
| 1076. AN6250.1.NCU01803.1.MG06681.1.FG10878.1 | 0 | 0 | 0 | 1 | 10 | 0 |
| 1077. AN6251.1.NCU01804.1.MG06680.1.FG10876.1 | 4 | 0 | 0 | 0 | 0 | 0 |
| 1078. AN6255.1.NCU06741.1.MG01111.1.FG06268.1 | 8 | 0 | 0 | 0 | 3 | 0 |
| 1079. AN6256.1.NCU06739.1.MG01109.1.FG06266.1 | 4 | 0 | 0 | 0 | 0 | 0 |
| 1080. AN6265.1.NCU01502.1.MG01100.1.FG04404.1 | 0 | 1 | 1 | 3 | 0 | 0 |
| 1081. AN6266.1.NCU01503.1.MG01087.1.FG04403.1 | 4 | 0 | 0 | 0 | 0 | 0 |
| 1082. AN6267.1.NCU01674.1.MG01121.1.FG06277.1 | 4 | 0 | 0 | 1 | 2 | 0 |
| 1083. AN6269.1.NCU06333.1.MG06172.1.FG06177.1 | 4 | 0 | 0 | 0 | 1 | 0 |
| 1084. AN6272.1.NCU08005.1.MG01718.1.FG01927.1 | 0 | 0 | 0 | 2 | 5 | 0 |
| 1085. AN6273.1.NCU07787.1.MG05344.1.FG11205.1 | 0 | 1 | 0 | 0 | 1 | 0 |
| 1086. AN6287.1.NCU01606.1.MG03152.1.FG00300.1 | 12 | 0 | 0 | 0 | 0 | 0 |
| 1087. AN6288.1.NCU01605.1.MG03151.1.FG00301.1 | 4 | 0 | 0 | 2 | 3 | 0 |
| 1088. AN6293.1.NCU03352.1.MG03261.1.FG01562.1 | 8 | 0 | 0 | 0 | 8 | 0 |
| 1089. AN6299.1.NCU04457.1.MG08036.1.FG09673.1 | 4 | 0 | 0 | 0 | 0 | 0 |
| 1090. AN6300.1.NCU06769.1.MG03202.1.FG00329.1 | 8 | 0 | 0 | 3 | 0 | 0 |
| 1091. AN6307.1.NCU06242.1.MG06367.1.FG07250.1 | 8 | 0 | 0 | 2 | 1 | 0 |
| 1092. AN6330.1.NCU07700.1.MG01742.1.FG09574.1 | 0 | 2 | 0 | 1 | 4 | 0 |
| 1093. AN6338.1.NCU09116.1.MG09919.1.FG01285.1 | 4 | 0 | 0 | 2 | 2 | 0 |
| 1094. AN6346.1.NCU04579.1.MG05345.1.FG02056.1 | 0 | 0 | 0 | 1 | 1 | 0 |
| 1095. AN6364.1.NCU07554.1.MG04988.1.FG06754.1 | 8 | 0 | 0 | 1 | 3 | 0 |
| 1096. AN6366.1.NCU00216.1.MG06289.1.FG06947.1 | 0 | 0 | 1 | 6 | 1 | 0 |
| 1097. AN6368.1.NCU08195.1.MG09080.1.FG06576.1 | 4 | 0 | 0 | 1 | 2 | 0 |
| 1098. AN6369.1.NCU00290.1.MG08918.1.FG05589.1 | 0 | 1 | 3 | 7 | 4 | 3 |
| 1099. AN6374.1.NCU07070.1.MG04993.1.FG02752.1 | 0 | 3 | 1 | 3 | 2 | 0 |
| 1100. AN6394.1.NCU08924.1.MG05949.1.FG10285.1 | 0 | 6 | 0 | 2 | 2 | 0 |
| Ortholog | Totally Conserved Introns | Gained Introns | Lost Introns | Other Introns | Fail/Homology Positions | Fail/Nearby Intron Positions |
| 1101. AN6443.1.NCU04161.1.MG08309.1.FG11028.1 | 0 | 1 | 0 | 0 | 3 | 0 |
| 1102. AN6453.1.NCU02196.1.MG06615.1.FG00162.1 | 0 | 0 | 1 | 2 | 4 | 0 |
| 1103. AN6487.1.NCU00338.1.MG06327.1.FG05471.1 | 0 | 0 | 0 | 1 | 3 | 0 |
| 1104. AN6492.1.NCU00116.1.MG00472.1.FG02608.1 | 0 | 0 | 0 | 2 | 5 | 0 |
| 1105. AN6493.1.NCU00117.1.MG00471.1.FG02609.1 | 8 | 0 | 1 | 5 | 9 | 0 |
| 1106. AN6499.1.NCU06211.1.MG08835.1.FG02504.1 | 12 | 0 | 0 | 1 | 4 | 0 |
| 1107. AN6502.1.NCU04180.1.MG03974.1.FG07341.1 | 0 | 0 | 0 | 1 | 10 | 0 |
| 1108. AN6506.1.NCU06207.1.MG08832.1.FG02502.1 | 4 | 0 | 0 | 0 | 0 | 0 |
| 1109. AN6510.1.NCU01179.1.MG08637.1.FG07331.1 | 8 | 2 | 0 | 0 | 0 | 0 |
| 1110. AN6515.1.NCU01190.1.MG08069.1.FG07320.1 | 0 | 0 | 0 | 4 | 2 | 0 |
| 1111. AN6521.1.NCU08898.1.MG04842.1.FG10949.1 | 0 | 6 | 0 | 1 | 1 | 0 |
| 1112. AN6522.1.NCU04149.1.MG04070.1.FG05220.1 | 0 | 0 | 1 | 2 | 4 | 0 |
| 1113. AN6525.1.NCU03813.1.MG04034.1.FG06127.1 | 0 | 0 | 2 | 5 | 3 | 0 |
| 1114. AN6532.1.NCU00148.1.MG08857.1.FG02599.1 | 4 | 0 | 0 | 0 | 0 | 0 |
| 1115. AN6536.1.NCU01300.1.MG07528.1.FG06093.1 | 4 | 0 | 0 | 0 | 0 | 0 |
| 1116. AN6542.1.NCU04173.1.MG03982.2.FG07335.1 | 16 | 0 | 0 | 0 | 2 | 0 |
| 1117. AN6544.1.NCU04188.1.MG03971.1.FG07322.1 | 0 | 0 | 0 | 1 | 4 | 0 |
| 1118. AN6546.1.NCU06713.1.MG04054.1.FG05194.1 | 4 | 0 | 0 | 1 | 0 | 0 |
| 1119. AN6547.1.NCU06712.1.MG04053.1.FG05222.1 | 8 | 0 | 0 | 0 | 1 | 0 |
| 1120. AN6549.1.NCU03809.1.MG04038.1.FG06100.1 | 0 | 0 | 0 | 1 | 1 | 0 |
| 1121. AN6551.1.NCU03811.1.MG04037.1.FG06097.1 | 0 | 0 | 0 | 1 | 9 | 0 |
| 1122. AN6557.1.NCU00418.1.MG06310.1.FG06936.1 | 8 | 0 | 0 | 0 | 2 | 0 |
| 1123. AN6558.1.NCU00419.1.MG06311.1.FG06937.1 | 8 | 0 | 0 | 0 | 1 | 0 |
| 1124. AN6563.1.NCU03826.1.MG06936.1.FG07401.1 | 4 | 0 | 1 | 6 | 1 | 0 |
| 1125. AN6564.1.NCU03827.1.MG06935.1.FG07402.1 | 4 | 0 | 0 | 0 | 2 | 0 |
| Ortholog | Totally Conserved Introns | Gained Introns | Lost Introns | Other Introns | Fail/Homology Positions | Fail/Nearby Intron Positions |
| 1126. AN6566.1.NCU03833.1.MG06933.1.FG07404.1 | 4 | 0 | 0 | 0 | 3 | 0 |
| 1127. AN6567.1.NCU03834.1.MG06932.1.FG07405.1 | 8 | 0 | 0 | 1 | 1 | 0 |
| 1128. AN6569.1.NCU03836.1.MG06931.1.FG07406.1 | 0 | 0 | 0 | 1 | 2 | 0 |
| 1129. AN6591.1.NCU04104.1.MG03994.1.FG07169.1 | 0 | 0 | 3 | 8 | 10 | 0 |
| 1130. AN6598.1.NCU08993.1.MG04982.1.FG06903.1 | 0 | 0 | 0 | 5 | 2 | 0 |
| 1131. AN6601.1.NCU06710.1.MG04052.1.FG05224.1 | 0 | 1 | 0 | 2 | 2 | 0 |
| 1132. AN6610.1.NCU03718.1.MG00727.1.FG07154.1 | 4 | 0 | 0 | 1 | 4 | 0 |
| 1133. AN6614.1.NCU03818.1.MG04066.1.FG05149.1 | 0 | 0 | 0 | 4 | 3 | 0 |
| 1134. AN6618.1.NCU03661.1.MG04067.1.FG05191.1 | 0 | 0 | 0 | 1 | 6 | 0 |
| 1135. AN6629.1.NCU00634.1.MG02659.1.FG08561.1 | 4 | 0 | 0 | 1 | 2 | 0 |
| 1136. AN6630.1.NCU00635.1.MG02660.1.FG08560.1 | 8 | 0 | 0 | 0 | 0 | 0 |
| 1137. AN6636.1.NCU07442.1.MG02766.1.FG08596.1 | 0 | 1 | 0 | 1 | 4 | 0 |
| 1138. AN6639.1.NCU00680.1.MG02836.1.FG08851.1 | 4 | 0 | 1 | 4 | 2 | 0 |
| 1139. AN6640.1.NCU08886.1.MG09071.1.FG02121.1 | 0 | 0 | 0 | 2 | 3 | 0 |
| 1140. AN6650.1.NCU02482.1.MG02617.1.FG00175.1 | 0 | 0 | 1 | 3 | 3 | 0 |
| 1141. AN6651.1.NCU09903.1.MG02572.1.FG08532.1 | 4 | 0 | 0 | 1 | 0 | 0 |
| 1142. AN6653.1.NCU10007.1.MG02813.1.FG08700.1 | 0 | 0 | 2 | 7 | 3 | 0 |
| 1143. AN6654.1.NCU02479.1.MG02538.1.FG00178.1 | 4 | 0 | 1 | 4 | 3 | 0 |
| 1144. AN6655.1.NCU02480.1.MG02612.1.FG00177.1 | 4 | 0 | 0 | 5 | 0 | 0 |
| 1145. AN6675.1.NCU00045.1.MG10196.1.FG07232.1 | 4 | 0 | 1 | 2 | 0 | 0 |
| 1146. AN6679.1.NCU03038.1.MG00349.1.FG07048.1 | 8 | 1 | 0 | 0 | 1 | 0 |
| 1147. AN6681.1.NCU03039.1.MG00348.1.FG07049.1 | 8 | 0 | 0 | 0 | 8 | 0 |
| 1148. AN6685.1.NCU03045.1.MG04703.1.FG07053.1 | 0 | 0 | 0 | 1 | 3 | 0 |
| 1149. AN6688.1.NCU08297.1.MG01521.1.FG05315.1 | 8 | 0 | 0 | 0 | 0 | 0 |
| 1150. AN6689.1.NCU06968.1.MG08006.1.FG04983.1 | 4 | 0 | 0 | 0 | 1 | 2 |
| Ortholog | Totally Conserved Introns | Gained Introns | Lost Introns | Other Introns | Fail/Homology Positions | Fail/Nearby Intron Positions |
| 1151. AN6690.1.NCU08278.1.MG06141.1.FG06222.1 | 4 | 0 | 0 | 0 | 2 | 0 |
| 1152. AN6695.1.NCU05267.1.MG06063.1.FG01948.1 | 0 | 1 | 1 | 8 | 10 | 0 |
| 1153. AN6700.1.NCU07922.1.MG09432.1.FG04181.1 | 0 | 1 | 0 | 0 | 3 | 0 |
| 1154. AN6703.1.NCU07607.1.MG09827.1.FG04204.1 | 0 | 0 | 1 | 7 | 2 | 0 |
| 1155. AN6705.1.NCU08003.1.MG01720.1.FG01925.1 | 4 | 0 | 0 | 0 | 3 | 0 |
| 1156. AN6708.1.NCU07659.1.MG09878.1.FG04171.1 | 4 | 0 | 0 | 0 | 2 | 0 |
| 1157. AN6709.1.NCU07658.1.MG09877.1.FG04168.1 | 0 | 0 | 0 | 3 | 10 | 0 |
| 1158. AN6711.1.NCU05254.1.MG01828.1.FG04261.1 | 0 | 0 | 1 | 3 | 4 | 0 |
| 1159. AN6717.1.NCU04899.1.MG09367.1.FG02461.1 | 4 | 0 | 0 | 3 | 0 | 0 |
| 1160. AN6726.1.NCU06764.1.MG07165.2.FG04410.1 | 8 | 0 | 0 | 0 | 2 | 0 |
| 1161. AN6731.1.NCU05259.1.MG01790.1.FG06184.1 | 0 | 1 | 1 | 3 | 1 | 0 |
| 1162. AN6732.1.NCU06617.1.MG09470.1.FG05235.1 | 4 | 0 | 0 | 3 | 2 | 0 |
| 1163. AN6733.1.NCU05242.1.MG01776.1.FG09770.1 | 0 | 0 | 0 | 3 | 3 | 0 |
| 1164. AN6735.1.NCU06354.1.MG01450.1.FG02746.1 | 4 | 3 | 0 | 0 | 1 | 0 |
| 1165. AN6738.1.NCU04463.1.MG05095.1.FG05671.1 | 4 | 0 | 0 | 0 | 4 | 0 |
| 1166. AN6743.1.NCU01000.1.MG08785.1.FG04970.1 | 0 | 0 | 1 | 3 | 4 | 0 |
| 1167. AN6783.1.NCU04195.1.MG04868.1.FG07858.1 | 0 | 0 | 0 | 4 | 8 | 0 |
| 1168. AN6817.1.NCU02299.1.MG10413.1.FG10920.1 | 0 | 0 | 1 | 5 | 3 | 0 |
| 1169. AN6824.1.NCU03741.1.MG08159.1.FG07150.1 | 4 | 0 | 0 | 0 | 4 | 0 |
| 1170. AN6842.1.NCU01473.1.MG04007.1.FG09002.1 | 4 | 0 | 0 | 0 | 1 | 0 |
| 1171. AN6844.1.NCU03777.1.MG04012.1.FG07019.1 | 0 | 0 | 0 | 1 | 5 | 0 |
| 1172. AN6845.1.NCU08804.1.MG08460.1.FG05096.1 | 0 | 0 | 1 | 3 | 7 | 0 |
| 1173. AN6853.1.NCU03596.1.MG00707.1.FG05890.1 | 4 | 0 | 0 | 0 | 0 | 0 |
| 1174. AN6855.1.NCU03597.1.MG00706.1.FG07021.1 | 4 | 0 | 2 | 2 | 3 | 0 |
| 1175. AN6866.1.NCU07725.1.MG01710.1.FG09602.1 | 0 | 1 | 0 | 0 | 3 | 0 |
| Ortholog | Totally Conserved Introns | Gained Introns | Lost Introns | Other Introns | Fail/Homology Positions | Fail/Nearby Intron Positions |
| 1176. AN6869.1.NCU01348.1.MG10533.1.FG02206.1 | 8 | 1 | 1 | 8 | 1 | 0 |
| 1177. AN6870.1.NCU04672.1.MG10534.1.FG02207.1 | 0 | 0 | 2 | 2 | 0 | 0 |
| 1178. AN6874.1.NCU03503.1.MG02384.1.FG01640.1 | 0 | 0 | 2 | 5 | 3 | 0 |
| 1179. AN6889.1.NCU00445.1.MG05205.1.FG04109.1 | 4 | 1 | 0 | 3 | 4 | 0 |
| 1180. AN6892.1.NCU00434.1.MG05207.1.FG04111.1 | 4 | 0 | 0 | 0 | 0 | 0 |
| 1181. AN6894.1.NCU00181.1.MG06417.1.FG05403.1 | 0 | 1 | 0 | 4 | 1 | 0 |
| 1182. AN6897.1.NCU00436.1.MG05209.1.FG04113.1 | 0 | 0 | 0 | 1 | 1 | 0 |
| 1183. AN6898.1.NCU00435.1.MG05208.1.FG04112.1 | 0 | 0 | 1 | 3 | 4 | 0 |
| 1184. AN6900.1.NCU07550.1.MG08905.1.FG06702.1 | 0 | 0 | 0 | 2 | 4 | 0 |
| 1185. AN6902.1.NCU07547.1.MG08908.1.FG06705.1 | 4 | 0 | 0 | 0 | 1 | 0 |
| 1186. AN6904.1.NCU07549.1.MG08906.1.FG06703.1 | 4 | 0 | 0 | 0 | 2 | 0 |
| 1187. AN6907.1.NCU06278.1.MG05265.1.FG02570.1 | 0 | 0 | 2 | 6 | 4 | 0 |
| 1188. AN6911.1.NCU06268.1.MG05256.1.FG02565.1 | 0 | 1 | 0 | 4 | 7 | 0 |
| 1189. AN6915.1.NCU03714.1.MG08147.1.FG05144.1 | 0 | 0 | 0 | 2 | 3 | 0 |
| 1190. AN6922.1.NCU01795.1.MG07010.1.FG10829.1 | 4 | 0 | 0 | 1 | 1 | 0 |
| 1191. AN6933.1.NCU02906.1.MG08562.1.FG00805.1 | 0 | 0 | 3 | 7 | 4 | 0 |
| 1192. AN6945.1.NCU02328.1.MG07992.1.FG10331.1 | 0 | 2 | 1 | 3 | 6 | 0 |
| 1193. AN6974.1.NCU06404.1.MG06135.1.FG06209.1 | 8 | 0 | 0 | 0 | 0 | 0 |
| 1194. AN6976.1.NCU01859.1.MG09006.1.FG11619.1 | 12 | 0 | 0 | 1 | 1 | 0 |
| 1195. AN6978.1.NCU05513.1.MG00408.1.FG08173.1 | 0 | 0 | 1 | 3 | 1 | 0 |
| 1196. AN6985.1.NCU01701.1.MG08591.1.FG01430.1 | 4 | 0 | 0 | 0 | 6 | 0 |
| 1197. AN6988.1.NCU05363.1.MG01380.2.FG01605.1 | 4 | 0 | 0 | 1 | 1 | 0 |
| 1198. AN6992.1.NCU05544.1.MG10055.1.FG08277.1 | 8 | 0 | 0 | 0 | 0 | 0 |
| 1199. AN6993.1.NCU05542.1.MG10052.1.FG08275.1 | 4 | 0 | 0 | 2 | 1 | 0 |
| 1200. AN7000.1.NCU08471.1.MG06977.1.FG10885.1 | 4 | 0 | 0 | 7 | 3 | 0 |
| Ortholog | Totally Conserved Introns | Gained Introns | Lost Introns | Other Introns | Fail/Homology Positions | Fail/Nearby Intron Positions |
| 1201. AN7014.1.NCU07866.1.MG06395.1.FG05423.1 | 4 | 1 | 0 | 0 | 5 | 0 |
| 1202. AN7015.1.NCU05523.1.MG00397.1.FG08299.1 | 0 | 0 | 1 | 3 | 1 | 0 |
| 1203. AN7025.1.NCU06609.1.MG08050.1.FG05254.1 | 0 | 0 | 1 | 3 | 1 | 0 |
| 1204. AN7027.1.NCU09027.1.MG02315.1.FG00787.1 | 0 | 3 | 0 | 7 | 7 | 0 |
| 1205. AN7029.1.NCU04481.1.MG10169.1.FG09624.1 | 0 | 0 | 0 | 3 | 12 | 0 |
| 1206. AN7030.1.NCU06593.1.MG01702.1.FG05261.1 | 4 | 0 | 0 | 0 | 5 | 0 |
| 1207. AN7031.1.NCU06594.1.MG01703.1.FG05262.1 | 4 | 0 | 0 | 0 | 4 | 0 |
| 1208. AN7035.1.NCU04479.1.MG10171.1.FG05245.1 | 4 | 1 | 0 | 2 | 2 | 0 |
| 1209. AN7042.1.NCU06587.1.MG01707.1.FG05267.1 | 0 | 0 | 0 | 1 | 3 | 0 |
| 1210. AN7044.1.NCU06974.1.MG06040.1.FG05635.1 | 0 | 0 | 1 | 3 | 3 | 0 |
| 1211. AN7047.1.NCU07988.1.MG07075.1.FG01933.1 | 0 | 0 | 1 | 5 | 1 | 0 |
| 1212. AN7051.1.NCU07987.1.MG07074.1.FG01932.1 | 0 | 0 | 0 | 1 | 2 | 0 |
| 1213. AN7052.1.NCU06596.1.MG01705.1.FG05260.1 | 4 | 0 | 0 | 0 | 0 | 0 |
| 1214. AN7105.1.NCU07831.1.MG05243.1.FG02544.1 | 4 | 0 | 0 | 3 | 0 | 0 |
| 1215. AN7108.1.NCU08827.1.MG06149.1.FG09644.1 | 0 | 0 | 0 | 3 | 6 | 0 |
| 1216. AN7111.1.NCU08828.1.MG06148.1.FG09643.1 | 8 | 1 | 0 | 3 | 4 | 0 |
| 1217. AN7141.1.NCU08669.1.MG05814.1.FG09762.1 | 4 | 1 | 0 | 0 | 2 | 0 |
| 1218. AN7143.1.NCU03009.1.MG10565.1.FG02785.1 | 4 | 0 | 0 | 1 | 0 | 0 |
| 1219. AN7146.1.NCU03006.1.MG10568.1.FG02783.1 | 0 | 0 | 2 | 5 | 1 | 0 |
| 1220. AN7161.1.NCU01849.1.MG08431.1.FG01982.1 | 0 | 2 | 1 | 4 | 2 | 0 |
| 1221. AN7185.1.NCU09202.1.MG10596.1.FG02795.1 | 4 | 0 | 0 | 0 | 6 | 0 |
| 1222. AN7194.1.NCU01419.1.MG00738.1.FG10124.1 | 0 | 0 | 0 | 1 | 1 | 0 |
| 1223. AN7230.1.NCU00206.1.MG11036.1.FG09085.1 | 0 | 1 | 1 | 5 | 11 | 0 |
| 1224. AN7252.1.NCU00455.1.MG05199.1.FG04101.1 | 4 | 0 | 0 | 0 | 1 | 0 |
| 1225. AN7254.1.NCU00018.1.MG05193.1.FG05530.1 | 12 | 0 | 1 | 3 | 1 | 0 |
| Ortholog | Totally Conserved Introns | Gained Introns | Lost Introns | Other Introns | Fail/Homology Positions | Fail/Nearby Intron Positions |
| 1226. AN7290.1.NCU00348.1.MG05173.1.FG06746.1 | 0 | 1 | 1 | 2 | 2 | 0 |
| 1227. AN7293.1.NCU07563.1.MG00127.1.FG06758.1 | 0 | 0 | 1 | 2 | 5 | 0 |
| 1228. AN7299.1.NCU00422.1.MG06316.1.FG06955.1 | 4 | 0 | 1 | 4 | 2 | 0 |
| 1229. AN7301.1.NCU00163.1.MG05686.1.FG02528.1 | 0 | 0 | 1 | 5 | 6 | 0 |
| 1230. AN7302.1.NCU00162.1.MG05685.1.FG02529.1 | 0 | 0 | 0 | 1 | 5 | 0 |
| 1231. AN7303.1.NCU00164.1.MG05687.1.FG02527.1 | 4 | 0 | 0 | 0 | 9 | 1 |
| 1232. AN7305.1.NCU08668.1.MG05815.1.FG09761.1 | 0 | 1 | 0 | 1 | 0 | 0 |
| 1233. AN7307.1.NCU09737.1.MG04128.1.FG04255.1 | 0 | 0 | 1 | 3 | 3 | 0 |
| 1234. AN7313.1.NCU06861.1.MG03844.1.FG11366.1 | 0 | 0 | 0 | 2 | 5 | 0 |
| 1235. AN7325.1.NCU01192.1.MG08071.1.FG07175.1 | 4 | 0 | 0 | 4 | 1 | 0 |
| 1236. AN7353.1.NCU09794.1.MG08115.1.FG02628.1 | 0 | 0 | 0 | 1 | 7 | 0 |
| 1237. AN7354.1.NCU00464.1.MG05248.1.FG04137.1 | 4 | 0 | 0 | 0 | 3 | 0 |
| 1238. AN7360.1.NCU00465.1.MG05249.1.FG04121.1 | 0 | 0 | 0 | 1 | 5 | 0 |
| 1239. AN7370.1.NCU04396.1.MG09631.1.FG00850.1 | 0 | 0 | 1 | 6 | 11 | 0 |
| 1240. AN7388.1.NCU05770.1.MG04337.1.FG02974.1 | 0 | 1 | 0 | 0 | 1 | 0 |
| 1241. AN7401.1.NCU04997.1.MG09792.1.FG06445.1 | 0 | 1 | 1 | 3 | 8 | 0 |
| 1242. AN7402.1.NCU01517.1.MG01096.1.FG06278.1 | 0 | 0 | 2 | 5 | 8 | 0 |
| 1243. AN7422.1.NCU05777.1.MG08270.1.FG02640.1 | 4 | 0 | 0 | 0 | 5 | 0 |
| 1244. AN7423.1.NCU03606.1.MG00685.1.FG10122.1 | 8 | 1 | 0 | 2 | 1 | 0 |
| 1245. AN7428.1.NCU06672.1.MG07297.1.FG10226.1 | 4 | 0 | 0 | 1 | 7 | 0 |
| 1246. AN7430.1.NCU07156.1.MG00253.1.FG09984.1 | 0 | 0 | 0 | 1 | 1 | 0 |
| 1247. AN7435.1.NCU07482.1.MG03632.1.FG00911.1 | 4 | 0 | 0 | 2 | 2 | 0 |
| 1248. AN7444.1.NCU04241.1.MG06874.1.FG10274.1 | 4 | 0 | 0 | 0 | 14 | 0 |
| 1249. AN7451.1.NCU00461.1.MG05247.1.FG04117.1 | 0 | 3 | 0 | 1 | 3 | 0 |
| 1250. AN7463.1.NCU03257.1.MG00537.1.FG00529.1 | 4 | 1 | 3 | 5 | 1 | 0 |
| Ortholog | Totally Conserved Introns | Gained Introns | Lost Introns | Other Introns | Fail/Homology Positions | Fail/Nearby Intron Positions |
| 1251. AN7465.1.NCU03291.1.MG09891.1.FG00509.1 | 4 | 0 | 0 | 0 | 0 | 0 |
| 1252. AN7479.1.NCU06457.1.MG08897.1.FG08865.1 | 0 | 4 | 0 | 1 | 0 | 0 |
| 1253. AN7487.1.NCU06454.1.MG00466.1.FG05447.1 | 8 | 0 | 0 | 0 | 2 | 0 |
| 1254. AN7489.1.NCU00056.1.MG10202.1.FG07226.1 | 4 | 0 | 0 | 1 | 4 | 0 |
| 1255. AN7492.1.NCU06998.1.MG01422.1.FG09810.1 | 4 | 0 | 0 | 1 | 1 | 0 |
| 1256. AN7497.1.NCU05221.1.MG04136.1.FG02474.1 | 8 | 0 | 0 | 0 | 0 | 0 |
| 1257. AN7500.1.NCU05225.1.MG04140.1.FG02477.1 | 0 | 2 | 0 | 2 | 2 | 0 |
| 1258. AN7517.1.NCU03793.1.MG07464.1.FG07378.1 | 0 | 0 | 0 | 1 | 8 | 0 |
| 1259. AN7540.1.NCU07380.1.MG00341.1.FG01314.1 | 4 | 0 | 0 | 3 | 0 | 0 |
| 1260. AN7542.1.NCU07379.1.MG00342.1.FG01313.1 | 8 | 0 | 0 | 0 | 9 | 0 |
| 1261. AN7567.1.NCU09803.1.MG06356.1.FG01317.1 | 4 | 0 | 0 | 0 | 1 | 0 |
| 1262. AN7572.1.NCU07378.1.MG00345.1.FG01312.1 | 4 | 0 | 0 | 0 | 2 | 0 |
| 1263. AN7576.1.NCU02689.1.MG04377.1.FG01204.1 | 0 | 0 | 0 | 1 | 5 | 0 |
| 1264. AN7579.1.NCU02982.1.MG04506.1.FG01123.1 | 4 | 0 | 0 | 0 | 0 | 0 |
| 1265. AN7588.1.NCU00519.1.MG00534.1.FG00589.1 | 0 | 0 | 1 | 5 | 1 | 0 |
| 1266. AN7600.1.NCU05238.1.MG04144.1.FG02482.1 | 8 | 0 | 0 | 0 | 2 | 0 |
| 1267. AN7603.1.NCU09747.1.MG06108.1.FG05300.1 | 12 | 0 | 1 | 3 | 0 | 0 |
| 1268. AN7606.1.NCU06804.1.MG03143.1.FG04302.1 | 0 | 0 | 2 | 6 | 8 | 0 |
| 1269. AN7629.1.NCU09002.1.MG01667.1.FG05564.1 | 4 | 0 | 0 | 0 | 1 | 0 |
| 1270. AN7632.1.NCU06652.1.MG06011.1.FG10200.1 | 0 | 0 | 3 | 9 | 3 | 0 |
| 1271. AN7636.1.NCU06303.1.MG07773.1.FG01630.1 | 0 | 0 | 2 | 2 | 2 | 0 |
| 1272. AN7640.1.NCU05143.1.MG00194.1.FG03916.1 | 0 | 0 | 0 | 1 | 3 | 0 |
| 1273. AN7649.1.NCU00198.1.MG06427.1.FG05543.1 | 4 | 0 | 0 | 0 | 1 | 0 |
| 1274. AN7658.1.NCU01161.1.MG07332.1.FG10361.1 | 4 | 0 | 0 | 3 | 3 | 0 |
| 1275. AN7659.1.NCU01160.1.MG07333.1.FG10087.1 | 8 | 0 | 0 | 0 | 3 | 0 |
| Ortholog | Totally Conserved Introns | Gained Introns | Lost Introns | Other Introns | Fail/Homology Positions | Fail/Nearby Intron Positions |
| 1276. AN7665.1.NCU03268.1.MG09936.1.FG01091.1 | 0 | 0 | 0 | 3 | 1 | 0 |
| 1277. AN7672.1.NCU02635.1.MG00171.1.FG06856.1 | 4 | 0 | 0 | 0 | 0 | 0 |
| 1278. AN7673.1.NCU06380.1.MG06060.1.FG05293.1 | 0 | 3 | 0 | 4 | 3 | 0 |
| 1279. AN7674.1.NCU09528.1.MG07449.1.FG05124.1 | 4 | 0 | 0 | 0 | 2 | 0 |
| 1280. AN7677.1.NCU06637.1.MG00759.1.FG10096.1 | 4 | 0 | 0 | 1 | 2 | 0 |
| 1281. AN7678.1.NCU06638.1.MG00760.1.FG10095.1 | 0 | 0 | 0 | 3 | 7 | 0 |
| 1282. AN7679.1.NCU04293.1.MG00949.1.FG10388.1 | 8 | 0 | 0 | 0 | 0 | 0 |
| 1283. AN7698.1.NCU08067.1.MG09125.1.FG09435.1 | 8 | 0 | 0 | 1 | 0 | 0 |
| 1284. AN7705.1.NCU00879.1.MG10588.1.FG02812.1 | 4 | 0 | 1 | 5 | 2 | 0 |
| 1285. AN7707.1.NCU06429.1.MG06475.1.FG07284.1 | 4 | 2 | 1 | 3 | 1 | 0 |
| 1286. AN7709.1.NCU03730.1.MG08167.1.FG05131.1 | 0 | 0 | 1 | 3 | 3 | 0 |
| 1287. AN7715.1.NCU06386.1.MG06137.1.FG06214.1 | 0 | 0 | 1 | 3 | 1 | 0 |
| 1288. AN7721.1.NCU08897.1.MG04856.1.FG09016.1 | 4 | 0 | 0 | 2 | 1 | 0 |
| 1289. AN7723.1.NCU01370.1.MG09487.1.FG06122.1 | 0 | 0 | 0 | 3 | 0 | 0 |
| 1290. AN7725.1.NCU06550.1.MG05980.1.FG05035.1 | 0 | 0 | 0 | 2 | 0 | 0 |
| 1291. AN7736.1.NCU02147.1.MG03061.1.FG08845.1 | 0 | 0 | 0 | 2 | 1 | 0 |
| 1292. AN7741.1.NCU09673.1.MG05484.1.FG02105.1 | 4 | 0 | 0 | 0 | 0 | 0 |
| 1293. AN7744.1.NCU00127.1.MG04836.1.FG07271.1 | 4 | 0 | 0 | 0 | 4 | 0 |
| 1294. AN7750.1.NCU06389.1.MG06138.1.FG06212.1 | 0 | 0 | 0 | 1 | 13 | 0 |
| 1295. AN7752.1.NCU08291.1.MG01513.1.FG05316.1 | 8 | 0 | 0 | 0 | 0 | 0 |
| 1296. AN7755.1.NCU01954.1.MG02960.1.FG08901.1 | 0 | 0 | 0 | 2 | 0 | 0 |
| 1297. AN7770.1.NCU07958.1.MG05138.1.FG00407.1 | 4 | 0 | 0 | 6 | 2 | 0 |
| 1298. AN7832.1.NCU04938.1.MG00779.1.FG11008.1 | 0 | 0 | 0 | 1 | 0 | 0 |
| 1299. AN7936.1.NCU07938.1.MG04153.1.FG09737.1 | 0 | 0 | 0 | 1 | 4 | 0 |
| 1300. AN7952.1.NCU00796.1.MG00722.1.FG06997.1 | 0 | 1 | 0 | 0 | 8 | 0 |
| Ortholog | Totally Conserved Introns | Gained Introns | Lost Introns | Other Introns | Fail/Homology Positions | Fail/Nearby Intron Positions |
| 1301. AN7994.1.NCU01171.1.MG08646.1.FG06777.1 | 4 | 0 | 1 | 6 | 1 | 0 |
| 1302. AN8002.1.NCU01377.1.MG07440.1.FG06092.1 | 4 | 0 | 0 | 0 | 0 | 0 |
| 1303. AN8010.1.NCU06687.1.MG07289.1.FG06822.1 | 4 | 0 | 0 | 7 | 1 | 0 |
| 1304. AN8013.1.NCU01174.1.MG08648.1.FG06781.1 | 0 | 0 | 0 | 3 | 4 | 0 |
| 1305. AN8016.1.NCU01234.1.MG04885.1.FG05858.1 | 0 | 0 | 0 | 9 | 0 | 0 |
| 1306. AN8023.1.NCU04100.1.MG09517.1.FG07172.1 | 8 | 0 | 2 | 11 | 1 | 0 |
| 1307. AN8032.1.NCU01177.1.MG08631.1.FG06786.1 | 0 | 0 | 1 | 3 | 8 | 0 |
| 1308. AN8039.1.NCU05347.1.MG06213.1.FG01627.1 | 4 | 0 | 0 | 4 | 0 | 0 |
| 1309. AN8041.1.NCU01528.1.MG01084.1.FG06257.1 | 4 | 0 | 0 | 2 | 3 | 0 |
| 1310. AN8042.1.NCU01525.1.MG01076.1.FG04324.1 | 4 | 0 | 1 | 7 | 11 | 0 |
| 1311. AN8045.1.NCU09014.1.MG01676.1.FG06907.1 | 0 | 0 | 0 | 1 | 3 | 0 |
| 1312. AN8046.1.NCU03639.1.MG07016.1.FG05906.1 | 0 | 0 | 0 | 1 | 6 | 0 |
| 1313. AN8050.1.NCU09013.1.MG01675.1.FG06908.1 | 0 | 4 | 3 | 4 | 4 | 0 |
| 1314. AN8054.1.NCU06440.1.MG06472.1.FG07282.1 | 4 | 0 | 2 | 6 | 2 | 0 |
| 1315. AN8056.1.NCU06216.1.MG08841.1.FG02510.1 | 0 | 0 | 0 | 1 | 1 | 0 |
| 1316. AN8057.1.NCU06452.1.MG06466.1.FG07287.1 | 4 | 0 | 0 | 0 | 0 | 0 |
| 1317. AN8059.1.NCU03798.1.MG07469.1.FG06120.1 | 4 | 0 | 0 | 0 | 1 | 0 |
| 1318. AN8063.1.NCU07959.1.MG05139.1.FG00406.1 | 0 | 0 | 0 | 3 | 0 | 0 |
| 1319. AN8117.1.NCU06694.1.MG07280.1.FG07347.1 | 4 | 0 | 1 | 6 | 0 | 0 |
| 1320. AN8119.1.NCU01388.1.MG08182.1.FG07370.1 | 12 | 0 | 0 | 0 | 0 | 0 |
| 1321. AN8122.1.NCU00754.1.MG04158.1.FG09697.1 | 0 | 3 | 1 | 7 | 1 | 0 |
| 1322. AN8167.1.NCU01395.1.MG04078.1.FG05979.1 | 0 | 0 | 2 | 5 | 1 | 0 |
| 1323. AN8169.1.NCU03768.1.MG04017.1.FG07410.1 | 0 | 0 | 0 | 2 | 5 | 0 |
| 1324. AN8172.1.NCU03756.1.MG08189.1.FG07188.1 | 4 | 1 | 2 | 5 | 2 | 0 |
| 1325. AN8176.1.NCU03757.1.MG08190.1.FG07186.1 | 4 | 1 | 0 | 4 | 0 | 0 |
| Ortholog | Totally Conserved Introns | Gained Introns | Lost Introns | Other Introns | Fail/Homology Positions | Fail/Nearby Intron Positions |
| 1326. AN8181.1.NCU09541.1.MG09524.1.FG07005.1 | 4 | 0 | 0 | 1 | 2 | 0 |
| 1327. AN8182.1.NCU03795.1.MG07466.1.FG07380.1 | 12 | 0 | 0 | 2 | 0 | 0 |
| 1328. AN8183.1.NCU03794.1.MG07465.1.FG07379.1 | 4 | 0 | 0 | 2 | 2 | 0 |
| 1329. AN8187.1.NCU06679.1.MG07323.1.FG06798.1 | 0 | 0 | 2 | 6 | 12 | 0 |
| 1330. AN8188.1.NCU07774.1.MG09458.1.FG05854.1 | 0 | 1 | 1 | 3 | 0 | 0 |
| 1331. AN8194.1.NCU03800.1.MG07471.1.FG06115.1 | 4 | 0 | 1 | 3 | 2 | 0 |
| 1332. AN8204.1.NCU00293.1.MG04697.1.FG05580.1 | 0 | 0 | 2 | 2 | 3 | 0 |
| 1333. AN8215.1.NCU09545.1.MG08171.1.FG07127.1 | 0 | 0 | 0 | 1 | 0 | 0 |
| 1334. AN8216.1.NCU04202.1.MG08622.1.FG05972.1 | 0 | 2 | 4 | 10 | 2 | 0 |
| 1335. AN8224.1.NCU08894.1.MG05956.1.FG04582.1 | 0 | 1 | 0 | 0 | 5 | 0 |
| 1336. AN8225.1.NCU08893.1.MG05957.1.FG05932.1 | 4 | 0 | 0 | 0 | 1 | 0 |
| 1337. AN8232.1.NCU07757.1.MG04877.1.FG09011.1 | 4 | 0 | 0 | 0 | 1 | 0 |
| 1338. AN8258.1.NCU03623.1.MG04081.1.FG05977.1 | 4 | 0 | 0 | 0 | 0 | 0 |
| 1339. AN8261.1.NCU07580.1.MG04660.2.FG05393.1 | 0 | 0 | 1 | 4 | 1 | 0 |
| 1340. AN8274.1.NCU01689.1.MG07201.1.FG01421.1 | 4 | 0 | 0 | 3 | 2 | 0 |
| 1341. AN8275.1.NCU01692.1.MG07202.1.FG01422.1 | 12 | 1 | 0 | 6 | 0 | 0 |
| 1342. AN8277.1.NCU01652.1.MG07195.1.FG01417.1 | 12 | 1 | 1 | 4 | 0 | 0 |
| 1343. AN8280.1.NCU01654.1.MG07197.1.FG01419.1 | 4 | 0 | 0 | 0 | 6 | 0 |
| 1344. AN8283.1.NCU02624.1.MG04432.1.FG01000.1 | 0 | 0 | 1 | 3 | 2 | 0 |
| 1345. AN8286.1.NCU02626.1.MG04366.1.FG01004.1 | 0 | 1 | 0 | 0 | 3 | 0 |
| 1346. AN8291.1.NCU04652.1.MG07481.1.FG07432.1 | 0 | 0 | 1 | 3 | 12 | 0 |
| 1347. AN8293.1.NCU07011.1.MG06038.1.FG05081.1 | 0 | 0 | 0 | 6 | 2 | 0 |
| 1348. AN8363.1.NCU03255.1.MG00538.1.FG00530.1 | 0 | 0 | 1 | 6 | 10 | 0 |
| 1349. AN8401.1.NCU09923.1.MG08985.1.FG07993.1 | 0 | 0 | 1 | 2 | 2 | 0 |
| 1350. AN8485.1.NCU04315.1.MG00776.1.FG10144.1 | 0 | 1 | 1 | 3 | 0 | 0 |
| Ortholog | Totally Conserved Introns | Gained Introns | Lost Introns | Other Introns | Fail/Homology Positions | Fail/Nearby Intron Positions |
| 1351. AN8491.1.NCU00105.1.MG00482.1.FG06962.1 | 0 | 4 | 0 | 2 | 2 | 0 |
| 1352. AN8498.1.NCU00825.1.MG05855.1.FG06725.1 | 0 | 0 | 3 | 7 | 8 | 0 |
| 1353. AN8534.1.NCU01882.1.MG08279.1.FG11617.1 | 0 | 2 | 1 | 7 | 0 | 2 |
| 1354. AN8559.1.NCU03913.1.MG06511.1.FG08622.1 | 0 | 0 | 0 | 1 | 6 | 0 |
| 1355. AN8562.1.NCU08603.1.MG05656.1.FG00550.1 | 0 | 4 | 0 | 3 | 5 | 0 |
| 1356. AN8566.1.NCU04072.1.MG08983.1.FG03349.1 | 4 | 0 | 0 | 0 | 0 | 0 |
| 1357. AN8637.1.NCU08791.1.MG10061.1.FG06554.1 | 0 | 1 | 1 | 3 | 0 | 0 |
| 1358. AN8664.1.NCU02124.1.MG02570.1.FG08780.1 | 0 | 0 | 1 | 4 | 4 | 0 |
| 1359. AN8668.1.NCU09094.1.MG02761.1.FG08598.1 | 4 | 0 | 0 | 0 | 1 | 0 |
| 1360. AN8671.1.NCU02036.1.MG02784.1.FG08650.1 | 0 | 1 | 0 | 0 | 9 | 0 |
| 1361. AN8672.1.NCU07443.1.MG02767.1.FG08595.1 | 8 | 0 | 0 | 1 | 0 | 0 |
| 1362. AN8674.1.NCU07446.1.MG02770.1.FG08593.1 | 4 | 0 | 0 | 0 | 1 | 0 |
| 1363. AN8676.1.NCU07430.1.MG02773.1.FG08696.1 | 8 | 0 | 0 | 0 | 1 | 0 |
| 1364. AN8680.1.NCU00578.1.MG02997.1.FG08777.1 | 0 | 0 | 0 | 1 | 1 | 0 |
| 1365. AN8681.1.NCU00568.1.MG02998.1.FG08778.1 | 0 | 0 | 0 | 3 | 0 | 0 |
| 1366. AN8692.1.NCU03151.1.MG02710.1.FG08677.1 | 0 | 1 | 1 | 3 | 3 | 0 |
| 1367. AN8696.1.NCU02014.1.MG03665.1.FG08527.1 | 4 | 0 | 0 | 0 | 0 | 0 |
| 1368. AN8698.1.NCU01918.1.MG02971.1.FG08919.1 | 8 | 0 | 0 | 1 | 2 | 0 |
| 1369. AN8701.1.NCU02112.1.MG02569.1.FG08557.1 | 0 | 1 | 0 | 0 | 0 | 0 |
| 1370. AN8702.1.NCU02113.1.MG02568.1.FG08558.1 | 4 | 0 | 0 | 3 | 2 | 0 |
| 1371. AN8713.1.NCU07440.1.MG02804.1.FG08658.1 | 0 | 0 | 0 | 3 | 3 | 0 |
| 1372. AN8721.1.NCU01980.1.MG03717.1.FG08879.1 | 0 | 1 | 0 | 0 | 2 | 0 |
| 1373. AN8748.1.NCU02027.1.MG02811.1.FG06404.1 | 0 | 0 | 0 | 1 | 5 | 2 |
| 1374. AN8751.1.NCU09071.1.MG02757.1.FG08635.1 | 4 | 1 | 1 | 2 | 0 | 0 |
| 1375. AN8755.1.NCU02481.1.MG02616.1.FG00176.1 | 0 | 0 | 0 | 4 | 2 | 0 |
| Ortholog | Totally Conserved Introns | Gained Introns | Lost Introns | Other Introns | Fail/Homology Positions | Fail/Nearby Intron Positions |
| 1376. AN8763.1.NCU03156.1.MG02669.1.FG08566.1 | 4 | 0 | 2 | 6 | 1 | 0 |
| 1377. AN8782.1.NCU00173.1.MG05317.1.FG06681.1 | 0 | 1 | 0 | 4 | 1 | 0 |
| 1378. AN8785.1.NCU01241.1.MG07066.1.FG07131.1 | 8 | 0 | 0 | 0 | 1 | 0 |
| 1379. AN8787.1.NCU00134.1.MG10127.1.FG06659.1 | 0 | 0 | 0 | 1 | 5 | 0 |
| 1380. AN8796.1.NCU01099.1.MG04881.1.FG05865.1 | 0 | 0 | 1 | 3 | 0 | 0 |
| 1381. AN8800.1.NCU05642.1.MG04872.1.FG05963.1 | 0 | 3 | 0 | 0 | 5 | 0 |
| 1382. AN8801.1.NCU04803.1.MG09511.1.FG08435.1 | 0 | 2 | 1 | 4 | 2 | 0 |
| 1383. AN8812.1.NCU04021.1.MG00937.1.FG08373.1 | 0 | 1 | 0 | 0 | 6 | 0 |
| 1384. AN8820.1.NCU03804.1.MG07456.2.FG06103.1 | 8 | 0 | 0 | 1 | 1 | 0 |
| 1385. AN8825.1.NCU00269.1.MG01661.1.FG05558.1 | 4 | 0 | 0 | 1 | 5 | 0 |
| 1386. AN8828.1.NCU06176.1.MG06435.1.FG06941.1 | 8 | 0 | 0 | 1 | 1 | 0 |
| 1387. AN8831.1.NCU01069.1.MG05528.1.FG02092.1 | 0 | 1 | 3 | 10 | 3 | 0 |
| 1388. AN8834.1.NCU00404.1.MG06322.1.FG05466.1 | 0 | 2 | 0 | 0 | 1 | 2 |
| 1389. AN8838.1.NCU00095.1.MG06462.1.FG06948.1 | 0 | 0 | 0 | 4 | 3 | 0 |
| 1390. AN8843.1.NCU04277.1.MG09550.1.FG10211.1 | 8 | 0 | 0 | 0 | 1 | 0 |
| 1391. AN8848.1.NCU06198.1.MG08858.1.FG02699.1 | 4 | 0 | 1 | 3 | 2 | 0 |
| 1392. AN8853.1.NCU00410.1.MG06266.1.FG06927.1 | 8 | 0 | 0 | 3 | 0 | 0 |
| 1393. AN8856.1.NCU06226.1.MG10185.1.FG02493.1 | 0 | 1 | 1 | 3 | 3 | 1 |
| 1394. AN8857.1.NCU00417.1.MG06309.1.FG06935.1 | 0 | 0 | 0 | 1 | 3 | 0 |
| 1395. AN8859.1.NCU04118.1.MG06879.1.FG07421.1 | 4 | 0 | 1 | 2 | 6 | 0 |
| 1396. AN8862.1.NCU01440.1.MG06923.1.FG07469.1 | 16 | 0 | 1 | 3 | 2 | 0 |
| 1397. AN8863.1.NCU01438.1.MG06924.1.FG07467.1 | 4 | 0 | 0 | 0 | 5 | 1 |
| 1398. AN8868.1.NCU01444.1.MG06914.1.FG07473.1 | 4 | 0 | 2 | 2 | 1 | 0 |
| 1399. AN8869.1.NCU01446.1.MG06915.1.FG07475.1 | 8 | 1 | 0 | 0 | 1 | 0 |
| 1400. AN8870.1.NCU01452.1.MG06919.1.FG07480.1 | 12 | 0 | 0 | 0 | 2 | 0 |
| Ortholog | Totally Conserved Introns | Gained Introns | Lost Introns | Other Introns | Fail/Homology Positions | Fail/Nearby Intron Positions |
| 1401. AN8874.1.NCU09808.1.MG06361.1.FG01320.1 | 0 | 0 | 0 | 3 | 4 | 0 |
| 1402. AN8877.1.NCU03264.1.MG09944.1.FG06859.1 | 0 | 0 | 0 | 1 | 0 | 0 |
| 1403. AN8959.1.NCU02801.1.MG01585.1.FG01031.1 | 0 | 1 | 0 | 0 | 2 | 0 |
| 1404. AN8990.1.NCU05776.1.MG01385.1.FG06473.1 | 4 | 1 | 2 | 6 | 3 | 0 |
| 1405. AN9007.1.NCU06895.1.MG03375.1.FG06068.1 | 0 | 0 | 1 | 2 | 4 | 0 |
| 1406. AN9059.1.NCU02379.1.MG03552.1.FG06363.1 | 0 | 0 | 1 | 3 | 3 | 0 |
| 1407. AN9063.1.NCU00579.1.MG03047.1.FG08586.1 | 4 | 0 | 0 | 2 | 1 | 0 |
| 1408. AN9064.1.NCU00891.1.MG01176.1.FG04922.1 | 0 | 0 | 0 | 1 | 2 | 0 |
| 1409. AN9069.1.NCU00911.1.MG01191.1.FG00702.1 | 4 | 0 | 0 | 0 | 3 | 0 |
| 1410. AN9072.1.NCU00889.1.MG01179.1.FG00808.1 | 4 | 0 | 0 | 0 | 2 | 0 |
| 1411. AN9080.1.NCU09331.1.MG04426.1.FG00609.1 | 0 | 0 | 1 | 7 | 1 | 0 |
| 1412. AN9083.1.NCU02090.1.MG00657.1.FG00722.1 | 4 | 0 | 0 | 0 | 0 | 0 |
| 1413. AN9086.1.NCU05796.1.MG04694.1.FG02649.1 | 4 | 0 | 0 | 1 | 2 | 0 |
| 1414. AN9090.1.NCU00768.1.MG08741.1.FG05737.1 | 0 | 0 | 0 | 3 | 5 | 0 |
| 1415. AN9094.1.NCU02109.1.MG01320.1.FG00872.1 | 0 | 0 | 0 | 1 | 2 | 0 |
| 1416. AN9097.1.NCU07182.1.MG10680.1.FG04476.1 | 8 | 0 | 0 | 0 | 0 | 0 |
| 1417. AN9103.1.NCU05850.1.MG08262.1.FG02433.1 | 0 | 1 | 0 | 3 | 1 | 0 |
| 1418. AN9106.1.NCU03862.1.MG00773.1.FG10020.1 | 4 | 0 | 0 | 1 | 10 | 0 |
| 1419. AN9108.1.NCU01402.1.MG00755.1.FG09040.1 | 4 | 2 | 0 | 1 | 2 | 0 |
| 1420. AN9119.1.NCU05117.1.MG04717.1.FG02662.1 | 0 | 0 | 0 | 2 | 10 | 0 |
| 1421. AN9148.1.NCU02797.1.MG01631.1.FG00524.1 | 0 | 0 | 0 | 3 | 5 | 0 |
| 1422. AN9156.1.NCU09788.1.MG10669.1.FG09475.1 | 0 | 2 | 1 | 3 | 1 | 0 |
| 1423. AN9157.1.NCU07926.1.MG09393.1.FG02555.1 | 0 | 3 | 0 | 9 | 0 | 0 |
| 1424. AN9160.1.NCU03023.1.MG01605.1.FG00489.1 | 0 | 0 | 0 | 9 | 8 | 0 |
| 1425. AN9286.1.NCU07351.1.MG07646.1.FG03629.1 | 0 | 1 | 0 | 0 | 0 | 0 |
| Ortholog | Totally Conserved Introns | Gained Introns | Lost Introns | Other Introns | Fail/Homology Positions | Fail/Nearby Intron Positions |
| 1426. AN9339.1.NCU00355.1.MG06442.1.FG06733.1 | 4 | 3 | 1 | 6 | 4 | 0 |
| 1427. AN9362.1.NCU03261.1.MG09948.1.FG06860.1 | 0 | 2 | 1 | 3 | 0 | 0 |
| 1428. AN9403.1.NCU03004.1.MG10569.1.FG02782.1 | 0 | 0 | 0 | 2 | 1 | 0 |
| 1429. AN9408.1.NCU07307.1.MG04118.1.FG05322.1 | 0 | 0 | 0 | 4 | 2 | 0 |
| 1430. AN9411.1.NCU01765.1.MG03155.1.FG05198.1 | 0 | 0 | 0 | 1 | 5 | 0 |
| 1431. AN9425.1.NCU03086.1.MG01270.1.FG01221.1 | 0 | 1 | 0 | 0 | 12 | 0 |
| 1432. AN9436.1.NCU01625.1.MG03132.1.FG04295.1 | 0 | 0 | 3 | 7 | 4 | 0 |
| 1433. AN9438.1.NCU07993.1.MG01758.1.FG05090.1 | 0 | 0 | 1 | 3 | 1 | 0 |
| 1434. AN9459.1.NCU01381.1.MG09526.1.FG06088.1 | 0 | 1 | 0 | 0 | 5 | 0 |
| 1435. AN9460.1.NCU06677.1.MG07324.1.FG06800.1 | 0 | 0 | 0 | 1 | 2 | 0 |
| 1436. AN9461.1.NCU03796.1.MG07467.1.FG07381.1 | 0 | 0 | 1 | 5 | 5 | 0 |
| 1437. AN9465.1.NCU02744.1.MG04829.1.FG01154.1 | 8 | 0 | 3 | 5 | 0 | 0 |
| 1438. AN9469.1.NCU07852.1.MG05282.1.FG04125.1 | 4 | 0 | 0 | 1 | 0 | 0 |
| 1439. AN9470.1.NCU07853.1.MG05283.1.FG04126.1 | 4 | 0 | 0 | 0 | 1 | 0 |
| 1440. AN9490.1.NCU02195.1.MG06616.1.FG00161.1 | 0 | 0 | 3 | 6 | 6 | 0 |
| 1441. AN9496.1.NCU03752.1.MG00712.1.FG07151.1 | 0 | 2 | 0 | 5 | 2 | 0 |
| 1442. AN9498.1.NCU08664.1.MG01886.1.FG04190.1 | 0 | 0 | 2 | 2 | 3 | 0 |
| 1443. AN9504.1.NCU03187.1.MG03026.1.FG09408.1 | 4 | 0 | 0 | 4 | 1 | 0 |
| 1444. AN9522.1.NCU04099.1.MG09516.1.FG07173.1 | 0 | 0 | 1 | 3 | 0 | 0 |
| 1445. AN9526.1.NCU01907.1.MG02920.1.FG08940.1 | 0 | 2 | 1 | 5 | 0 | 2 |
| 1446. AN9527.1.NCU00147.1.MG06443.1.FG02600.1 | 4 | 0 | 0 | 0 | 2 | 0 |
| 1447. AN9538.1.NCU06503.1.MG00373.1.FG05399.1 | 4 | 0 | 0 | 1 | 3 | 0 |
| **Total** | **3912** | **618** | **604** | **3322** | **3855** | **81** |
